# The Effect of School Closure on Hand, Foot, and Mouth Disease Transmission in Singapore: A Modeling Approach

**DOI:** 10.4269/ajtmh.18-0099

**Published:** 2018-10-22

**Authors:** Yirong Chen, Hishamuddin Badaruddin, Vernon J. Lee, Jeffery Cutter, Alex R. Cook

**Affiliations:** 1 Saw Swee Hock School of Public Health, National University of Singapore, National University Health System, Singapore;; 2 Ministry of Health, Singapore

## Abstract

Singapore implements a school closure policy for institutional hand, foot, and mouth disease (HFMD) outbreaks, but there is a lack of empirical evidence on the effect of closure on HFMD transmission. We conducted a retrospective analysis of 197,207 cases of HFMD over the period 2003–2012 at the national level and of 57,502 cases in 10,080 institutional outbreaks over the period 2011–2016 in Singapore. The effects of school closure due to 1) institutional outbreaks, 2) public holidays, and 3) school vacations were assessed using a Bayesian time series modeling approach. School closure was associated with a reduction in HFMD transmission rate. During public holidays, average numbers of secondary cases having onset the week after dropped by 53% (95% credible interval 44–62%), and during school vacations, the number of secondary cases dropped by 7% (95% credible interval 3–10%). Schools being temporarily closed in response to an institutional outbreak reduced the average number of new cases by 1,204 (95% credible interval 1,140–1,297). Despite the positive effect in reducing transmission, the effect of school closure is relatively small and may not justify the routine use of this measure.

## Introduction

Hand, foot, and mouth disease (HFMD) is a common pediatric disease that is endemic in East and Southeast Asia^1,2^ and increasingly found in North America^3,4^ and Europe.^5^ When caused by Coxsackieviruses, HFMD usually leads to relatively mild infections with symptoms that are self-limiting.^6^ However, HFMD caused by human enterovirus 71 may lead to complications involving the nervous system and result in reduced cognitive function, delayed neurodevelopment, and motor impairment, or death^7–9^; in China, an estimated 350–900 children die of HFMD annually.^10^

Because of this potential severity, the Ministry of Health in Singapore imposes strict control policies, especially in preschools. In preschools, daily routine health checks for all children and isolation of suspected cases are implemented for early detection and control of outbreaks.^11^ Should transmission continue within an outbreak, school closure is enforced.^12^ This policy was implemented following an outbreak in 2000 in which several children died from enterovirus 71 complications^13,14^; the details have evolved over time, but until recently, if a school has more than 16 cases or an attack rate more than 23% with a transmission period more than 24 days, the school will be required to close for a period of 10 days.^15^ In the most updated guideline, the policy has been relaxed somewhat, and Ministry of Health will consider the predominant circulating strain when assessing closure in addition to the trigger. This change provides an avenue to assess the impact of this policy.

School closure as a form of social distancing intervention to mitigate transmission during an infectious disease outbreak^16^ is often found in countries’ pandemic preparedness plans.^17^ The influenza literature shows that school closure may effectively reduce the spread,^18–20^ and school vacations have a significant impact in limiting transmission.^21,22^ Analyses also suggest that school closure combined with the use of antiviral agents is cost-effective and is a justifiable strategy for mitigating influenza pandemics.^23,24^ There is, however, a lack of empirical evidence on the effect of school closure on HFMD transmission: one exception is a review of HFMD in Hong Kong that revealed fewer HFMD consultations than expected during the 2003 severe acute respiratory syndrome and the 2009 influenza pandemic, which was attributed to various control measures including school closure.^25,26^

This article aimed to assess the effect of school closure on HFMD transmission. In Singapore, HFMD is endemic with year-round transmission and is legally notifiable by physicians and childcare teachers, as well as actively screened for in preschool-aged children. These policies provide data that enable us to obtain three sources of information on the effect of school closure: 1) the reduction in the numbers of cases after a public holiday, when childcare centers and schools close; 2) the reduction during school vacations; and 3) the impact within childcare centers of school closure in response to an ongoing outbreak. Singapore’s school closure policy for HFMD, which has been implemented for over a decade, in tandem with a comprehensive HFMD surveillance system, therefore, provides a unique opportunity to assess the impact of this important method of outbreak control.

## Materials and Methods

### Source of data.

Ministry of Health, Singapore, actively monitors and publishes the incidence of HFMD, which was made a notifiable disease in the year 2000.^27^ Two sets of data on HFMD were extracted from the Ministry’s records for this study. The first dataset contains aggregate reported HFMD cases from 2003 to 2012, with the number of daily cases with onset of symptoms, stratified by age. The second dataset contains information on all HFMD outbreaks in childcare centers and kindergartens in Singapore, during the period 2011–2016. This provides the cumulative number of cases per day in each preschool with an outbreak, together with the school type (childcare centers or kindergarten), enrollment size, whether the schools were closed because of the outbreak, and, if so, dates of closure and reopening. Data were retained at a daily resolution for two analyses but aggregated to weekly for the analysis of vacations.

Data were collected under Singapore’s Infectious Disease Act, and because aggregate non-identifiable data were used, institutional review board approval was not deemed necessary for this study.

### Statistical analysis.

Separate statistical analyses were performed to investigate the 1) public holiday effect, 2) school vacation effect, and 3) school closure effect on HFMD transmission, as described in the following paragraphs.

### Public holiday effect.

There are typically 11 public holidays in Singapore each year, as detailed in Supplemental Table 1; these are a mix of secular and religious holidays, some of which rotate around the year following the lunar calendar. If a public holiday falls on Sunday, the following Monday will be a public holiday. We derived the dates of all public holidays from 2003 to 2012 from the official listing of the Ministry of Manpower.

The effect of public holiday on HFMD transmission was measured by quantifying the reduction attributable to the public holiday. Because time points not immediately preceding or following a public holiday contribute little information to the effect of the public holiday, rather than considering a time series model, we developed a Bayesian model of the time points surrounding public holidays. The number of cases in the week before the holiday i is modeled as $x_{i}∼\text{Poisson}\left({\text{α}}_{i}\times 7\text{μ}\right)$, and the number the week after as $y_{i}∼\text{Poisson}\left({\text{α}}_{i}\times \left[6\text{μ}+\text{θμ}\right]\right)$, or $y_{i}∼\text{Poisson}\left({\text{α}}_{i}\times \left[5\text{μ}+2\text{θμ}\right]\right)$ for two-day-long holidays. Here, μ represents the average number of cases on a typical day; estimates of θ and a 95% credible interval provide a measure of the public holiday effect (i.e., 1−θ is the reduction in the number of infections on a public holiday compared with that on a normal day); and ${\text{α}}_{i}∼\text{Γ}\left(a,a\right)$ is an individual week effect which has an expected value of 1 and allows for autocorrelation in the time series. The window length of 1 week was selected to ensure balance in the number of weekdays in each window and to correspond roughly to the assumed incubation period of HFMD of around 3–7 days.^2^ This model assumes that the difference between the number of cases prior or after the public holidays is purely because of the difference in the number of infections happening on the public holiday, which is unobservable but indirectly represented by the change in the number of symptomatic cases in the following weeks.

To assess whether there was any longer term impact, we repeated the analysis comparing incidence in the second week (days 8–14) following the holiday with the week before it, as well as in the third week (days 15–21).

The main analysis does not treat public holidays that were less than 7 days apart specially. In sensitivity analysis, we removed data corresponding to both holidays from the fitting procedure if they were less than 7 days apart. Sensitivity analysis was also conducted by applying different forms of the non-informative prior distributions for the parameters to test the robustness of the inference.

### School vacation effect.

In Singapore, kindergartens, primary schools, and secondary schools have school vacations in March for 1 week, in June for 4 weeks, in September for 1 week, and in November and December for 5–6 weeks. To measure the effect of school vacations on HFMD transmission, we built time series models, fit Bayesianly, for the weekly number of children with HFMD aged 12 years and younger. This has some similarities with an autoregressive time series, but the Bayesian approach afforded greater flexibility in the model specification.

We assumed a negative binomial distribution for the number of HFMD cases, $Y_{t}$, observed in week t, with mean

$${\text{μ}}_{t}=\left(a+b\times Y_{t-1}\right)\times d^{H_{t-1}}$$

and shape parameter p, where $H_{t}=1$ if week t is a school holiday and 0 otherwise, and *a*, *b*, *d*, *p*, are model parameters that needed to be estimated: b accounts for autocorrelation, whereas d determines the effect of school vacation. A negative binomial distribution is used to allow more flexibility to capture the observed variability in the data than that obtained using the Poisson model originally considered. A constant b that does not vary with time was used because Singapore has very little seasonality that would add forcing to the timing of epidemics of HFMD,^28^ and because it would hamper identifiability of the vacation effect. The adequacy of using a constant b was assessed and confirmed by examining the distribution of the residual of 1-week-ahead model forecasts. The model was fit using Bayesian methods (Markov chain Monte Carlo) with non-informative prior distributions, as described in the Supplemental File.

For each draw from the posterior distribution, we iteratively derived the median number of cases for the following week based on the number of cases in the current week using the formula in the aforementioned model. The number of cases in the first week of the simulations was taken to be the observed average weekly number of cases across the time horizon. This was done for 104 weeks (2 years) and only the latter 52 weeks were kept as the number of weekly cases for a typical year. For each set of posterior values, 1,000 such simulations were performed. Based on these 1,000 simulations of a typical year scenario using each of the 10,000 sets of posterior values, 95% prediction and credible intervals were derived for each of the 52 time points. Median values for all 1,000 simulated numbers for each set of posterior values (10,000 median values in total) were calculated.

### School closure during outbreaks effect.

For each preschool that had an outbreak during the time horizon 2011–2016, a period of 50 days from the first day of case onset was considered; this was enough to span the duration of most outbreaks. The number of incident cases on day j in outbreak i, $x_{i,j}$, was modeled by

$$x_{i,j}∼\text{Poisson }\left(\lambda_{c_{i,j}}\times s_{i,j-1}\times p^{w_{i,j-1}}\right)\text{,}$$

where $c_{i,j}$ is the cumulative number of cases within 1 week before day j in outbreak i, $s_{i,j}$ is the number of children not yet symptomatically infected during the current outbreak by day j of outbreak i, and $w_{i,j}$ is the indicator for school closure on day j in outbreak i ($w_{i,j}=1$ if the school was closed on day j in outbreak i, and 0 otherwise). We smoothed the effect ${\text{λ}}_{k}$ of the current outbreak size k using the formula

$$\log\left({\text{λ}}_{k}\right)∼N\left(\log\left({\text{λ}}_{k-1}\right),{\text{σ}}^{2}\right).$$

The posterior samples were used to project the number of cases that were avoided by forcing schools to close when they hit the trigger. For each draw from the posterior, 100 simulations were performed to each of the schools with closures in our study period. In each simulation, the number of new incident cases on each day after the closure day was calculated using the model formulation and $w_{i,j}$ was set to be 0. The total number of cases was then compared with the observed number of cases when schools close and the total number of additional cases due to not closing schools for each simulation/set of posterior values was recorded. Median values for all 100 simulated numbers for each set of posterior values (10,000 median values in total) were calculated.

We use Bayesian methods to fit the models as the Bayesian paradigm was considered more flexible than its frequentist analog. Throughout, non-informative prior distributions were selected (detailed in Supplemental File 1) and models were fit, using Markov chain Monte Carlo algorithms with burn-in periods of 1,000 and 10,000 iterations, in the R statistical environment.^29^ The posterior samples were then used to obtain posterior distributions of derived quantities. Throughout, equal-tailed 95% credible intervals are used. Model convergence was evaluated by using the Gelman diagnostic tests^30^ and model validity assessed by comparing posterior predictions or simulations with the actual observed data.

## Results

A total of 197,207 HFMD cases were notified to the Ministry of Health, Singapore, from 2003 to 2012. Table 1 shows the demographics of all notified cases. Children aged less than 12 years accounted for about 90% of all HFMD cases, and about 70% of all children with HFMD were aged 5 years and younger; 57% of the cases were male, whereas 74% were ethnic Chinese, 15% Malay, 3% Indian, and 8% others: the Indian ethnic group is substantially underrepresented among cases compared with the general population (11% of Singapore residents aged less than 12 years have Indian ethnicity).

**Table 1:** Demographics of all hand, foot, and mouth disease cases during 2003–2012

Demographic	No. (%)
Age (years)	
0–2	69,500 (35)
3–5	70,353 (36)
6–11	35,888 (18)
12–17	6,979 (4)
18 and older	14,487 (7)
Gender	
Female	85,784 (43)
Male	111,423 (57)
Ethnicity	
Chinese	145,012 (74)
Malay	29,308 (15)
Indian	6,559 (3)
Other	16,193 (8)

Table 2 shows the summary statistics for the Bayesian models for the public holiday effect: the average of 50 HFMD cases a day was reduced by 53% (95% confidence interval [CI]: [44%, 62%]) in the 1 week following a public holiday, but only by 34% (95% CI: [25%, 43%)]) in the second week, whereas there was no reduction in the third week (0%, 95% CI: [−10%, 9%]). Alternative forms of non-informative prior distribution did not affect the results (not shown).

**Table 2:** Observed number of cases 1 week before and 1/2/3 weeks after the public holidays and posterior mean and 95% confidence interval derived from posterior distribution of the public holiday effect for models based on 1 week before and the first (days 1–7), second (days 8–14), and third weeks (days 15–21) after the public holiday

Estimates	First-week model	Second-week model	Third-week model
Cases 1 week before (median, IQR)	41 (21, 66)	41 (21, 66)	41 (21, 66)
Cases 1/2/3 weeks after (median, IQR)	36 (22, 63)	37 (21, 60)	39 (24, 62)
Ratio: number of infections on PH to number of infections on a typical day (posterior mean and 95% credible interval)	0.47 (0.38, 0.56)	0.66 (0.57, 0.75)	1.0 (0.91, 1.1)

IQR = inter-quartile range; PH = public holiday.

**Figure 1.**
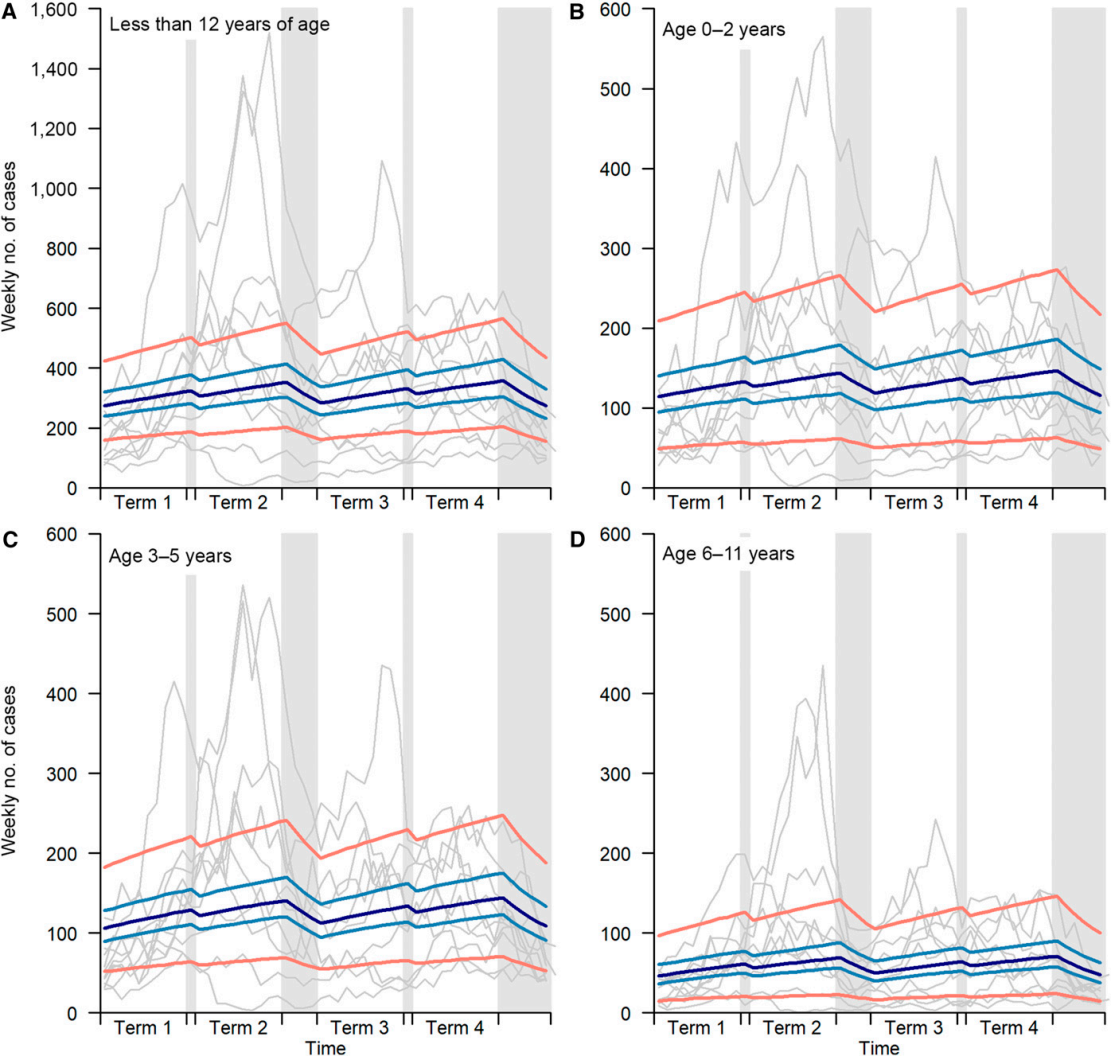
Regular school holiday effect. Effect of school vacations on hand, foot, and mouth disease transmission for school-age children is shown in the figure. Panel **A** shows the overall effect for all children less than 12 years of age. Panels **B**–**D** show the effect for infants (aged 0–2 years), preschool children (aged 3–5 years), and primary schoolchildren (aged 6–11 years), respectively. Gray bars indicate typical school vacations in a year. Gray lines are observed incident cases for years from 2003 to 2012. Dark blue lines are simulated average weekly incidents in a typical year and light blue lines and orange lines are 95% confidence interval and 95% prediction interval, respectively. This figure appears in color at www.ajtmh.org.

During school vacations, weekly number of cases were modeled to be reduced to 93% (95% CI: [0.90, 0.97]) relative to nonschool holidays for all children less than 12 years of age: 94% (95% CI: [0.90, 0.98]) for age 0–2 years, 93% (95% CI: [0.88, 0.97]) for age 3–5 years, and 90% (95% CI: [0.84, 0.96]) for age 6–11 years. Figure 1 shows the simulations for a typical year based on the modeled effects of school vacations on HFMD transmission, using typical timing of school holidays. Both overall analysis and separate models for different age groups showed similar results and are consistent with the patterns of the data for the 10-year period. The temporally structured distribution of residuals in the 1-week-ahead model forecasts is shown in Supplemental Figure 1. No trends were observed, suggesting that the model with a constant b is adequate.

**Figure 2.**
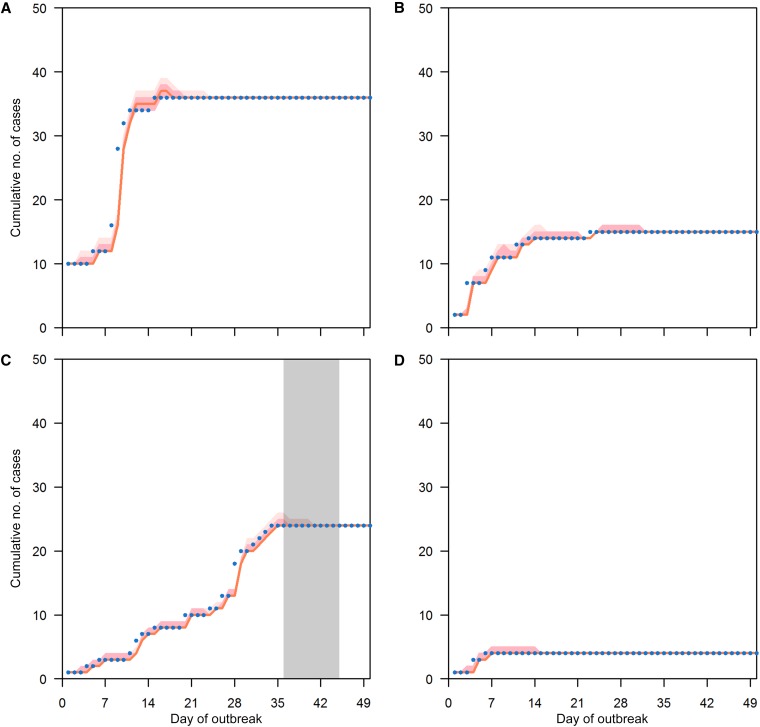
School closure during outbreaks effect. Panels **A**–**D** show one-day-ahead prediction on the cumulative number of cases for four randomly selected outbreaks (one with closure) based on the model. The gray bar means the school is closed during that period. Blue dots are observed cumulative number of cases. The orange line shows model fitting and light and dark pink shades show 95% and 70% credible intervals, respectively. This figure appears in color at www.ajtmh.org.

**Figure 3.**
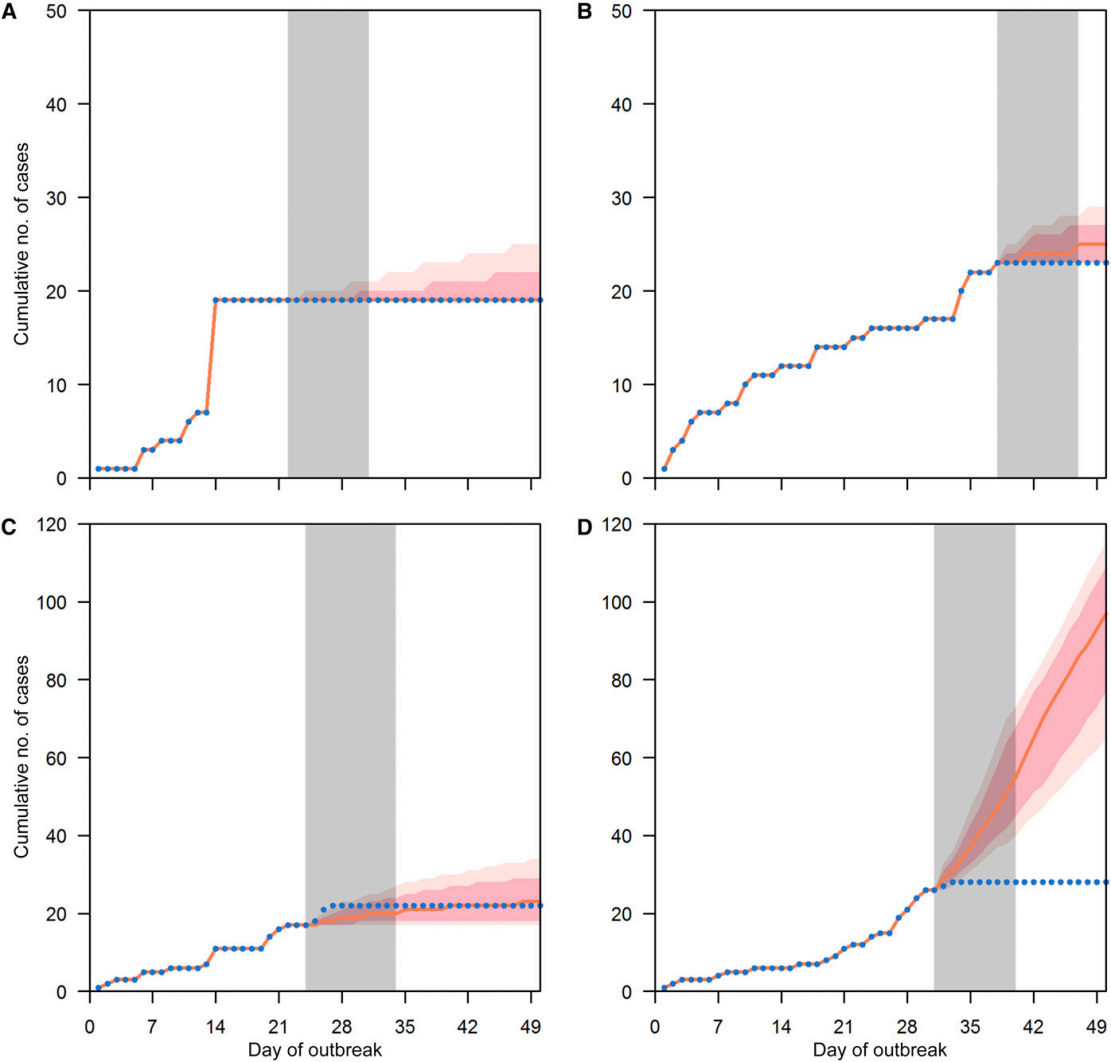
School closure during outbreaks effect. For outbreaks with school closure, panels **A**–**D** present the modeled cumulative number of cases if there were no school closure. Gray bars show the period when the school closes. Blue dots are observed cumulative number of cases. Orange line shows the cumulative number of cases if the school was not closed during the closure period. Light pink and dark pink shades are 95% and 70% confidence intervals, respectively. This figure appears in color at www.ajtmh.org.

**Figure 4.**
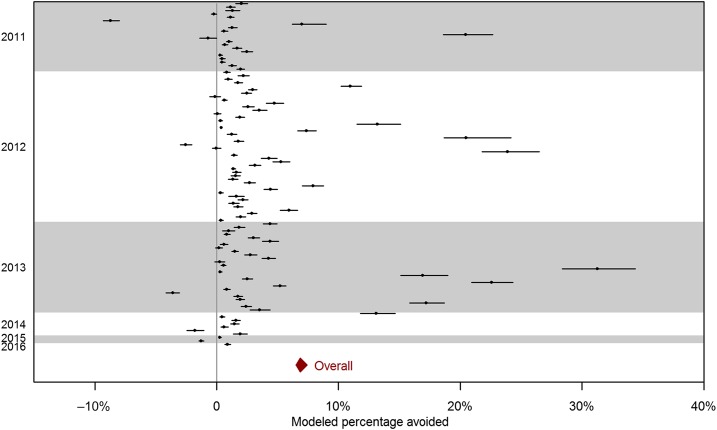
Modeled percentage of cases avoided from school closure for all outbreaks with closures. Black dots indicate the percentage of absolute number of cases avoided compared with school size and the lines are 95% confidence intervals. The red diamond indicates the overall number of cases avoided compared with the total enrolment size of schools with closures and its confidence interval. This figure appears in color at www.ajtmh.org.

From 2011 to 2016, there were totally 10,080 school-level outbreaks involving a total of 57,502 HFMD cases in childcare centers and kindergartens, of which 105 led to closure (Table 3). In all, 9,903 outbreaks including 100 closures were included in our model: 177 schools without accurate enrolment sizes were removed and four schools with closure falling beyond 50 days after the first day of outbreak were categorized as no closure (detailed in Supplemental File 1). The Bayesian Poisson model shows that the expected number of new cases decreases to 26% (95% CI: 21–32%) if a school was closed on that day compared with a normal school day, after adjusting for size and duration of the outbreak. One-day-ahead predictions based on the previous day’s observed number of cases, shown in Figure 2 for arbitrarily selected outbreaks (more outbreaks are presented in Supplemental File 2), both with and without short-term school closure, demonstrate that the fitted model adequately captures the observed outbreak patterns. Figure 3 show the effect of school closure by showing the cumulative number of cases if there were no school closure for four arbitrarily selected outbreaks with closure (more outbreaks are presented in Supplemental File 3). The percentage of cases avoided from school closure for all closures is shown in Figure 4. The majority of school closure events (> 80%) were associated with a less than 5% difference between observed and modeled cases, regardless of outbreak size at closure. Closures that prevented more than 10% of total school size were generally in bigger schools (of size more than 200). Overall, the modeled number of infections prevented through the school closure policy from 2011 to 2016 was 1,204 (95% CI: [1,140, 1,297]), that is, an average 2% reduction compared with the total number of cases during that period.

**Table 3:** Number of hand, foot, and mouth disease outbreaks and number of school closures each year from 2011 to 2016

Year	No. of outbreaks	No. of closures
2011	1,084	21
2012	2,033	47
2013	1,705	27
2014	1,220	7
2015	1,536	2
2016	2,502	1
Total	10,080	105

Convergence of all Bayesian models used was achieved according to the Gelman diagnostic test. Model validity assessed by comparing posterior predictions or simulations with the actual observed data is shown in Supplemental Figures 2– 5 and Supplemental File 2.

## Discussion

School closure is a common control measure in pandemic preparedness plans and in response to actual outbreaks, which may possibly be a high impact method of controlling an outbreak of a severe or potentially severe infectious disease. Its effectiveness has been assessed in modeling studies,^19,20,22^ which posit that because children have closer social networks and high contact rates in schools,^31^ closing schools may substantially reduce transmission, if they do not compensate by having greater contact outside of school during a closure. The burden of school closure on families which may need to make alternative childcare arrangements means that it is imperative to have real-world evidence supporting its effectiveness. Relatively little such evidence is available, though some studies in Japan, France and the United States have assessed school closures in response to influenza outbreaks.^18,32^ In light of this, the evidence on the effect of closure from Singapore’s long-standing routine school closures to control HFMD may prove valuable.

This study demonstrated a consistent reduction in average numbers of HFMD cases when schools were closed, regardless of the reason for the closure. There was an estimated decrease of 7% in the number of cases in the week after a public holiday than the week before it, corresponding to a reduction in the number of infections that occurs on the holiday itself of 53%, although the effect of the holiday declines in the weeks following as infections return to baseline levels. A similar reduction in the number of infections during school vacations was also estimated (also about 7%). This may be an underestimate of the actual effect size because many preschool-aged children still attend preschools during school holidays. This hypothesis is supported by the slightly accentuated effect in children who have started formal schooling, with a reduction in risk around 10% for children aged 6 years and older. Although the reduction was statistically significant for all age groups, the difference in effect between age groups was not statistically significant.

The data on school closure in response to outbreaks allowed a quasi-experimental analysis because recently the school closure policy has been reinterpreted to allow more discretion by the Ministry of Health whether to close the affected school or not. This led to a decrease in the number of school closures in the later periods of the study (Table 3). Although not randomized, this change allowed some overlap in the exposure (closure) and response (outbreak growth). The analysis showed that as with holidays and public holidays, school closure during an outbreak had the intended effect of mitigating transmission, but the effect was relatively small, and we estimated that only ∼1,200 cases were averted over the 6 years analyzed. The high asymptomatic rate^33,34^ and seroprevalence of the main causative viruses^35^ may mean that a substantial fraction of children are no longer susceptible by the time of closures in response to large outbreaks, which is currently triggered when more than 16 cases or 23% of children are symptomatic. The fraction of cases prevented by outbreak-induced closure was modeled to be small in general, but larger for the larger preschools, which we attribute to the structure of the thresholds for closure: this is effected when either the fraction or the number of children in the school is notified, meaning that closure of larger schools occurs when the threshold for the number of cases is hit but the fraction is still low. Given the disruptions to parents/families from unplanned closures,^11,36^ outbreak-driven closures may, therefore, cause more problems than closure because of holidays. In light of the limited effect, it is not clear that this policy should be continued to be used routinely for HFMD outbreaks, but the effect of closures because of holidays suggests that a school closure policy may still be valuable for pandemic preparedness plans and needs to be carried out when there are serious outbreaks or when the outbreak is due to novel pathogens of unknown severity.

Although this was a retrospective study, with HFMD being a legally notifiable disease in Singapore, incident data were collected in a standard way through preschools, clinics, and hospitals, and as such, the data used for this study are largely reliable indicators of symptomatic cases. However, some other limitations exist. Several assumptions were made in the modeling process: the public holiday effect analysis assumed that the observed difference in the number of cases before and after the public holiday was solely attributable to the public holiday. In temperate countries, this assumption may not be tenable because of seasonal changes that drive transmission. Singapore, however, lies close to the equator and has almost no seasonality—having year-round high temperature and humidity—with the effect that some virus transmission is effectively aseasonal.^28^ For the same reason, the assumption that there are no other temporal effects in the school vacation analysis may be more robust than it would in a temperate setting.

These three analyses provide evidence that school closure—whether in response to an outbreak, or because of a public holiday or longer school vacation—reduces the transmission of HFMD. Although we found that school closure in response to outbreaks does have a positive effect in reducing transmission, the effect is small, and given the high incidence and the fact that most of the cases of HFMD are mild and self-limiting, the evidence from this study suggests that policy-makers in Singapore should evaluate whether routine use of school closure outside of public health emergencies justifies the impact on families.

## Supplementary Material

Supplemental figure
