# Supplementary material for: The Effect of School Closure on Hand, Foot, and Mouth Disease Transmission in Singapore: A Modeling Approach
Source: Am J Trop Med Hyg. 2018 Oct 22;99(6):1625–32. doi: 10.4269/ajtmh.18-0099 (PMC6283473; doi:10.4269/ajtmh.18-0099)
Supplement: Supplementary file 1 [file tpmd180099.SD1.pdf]

Supplemental Material

In this document, we detail the schools that were removed from our school closure analysis and the details of the statistical models used.

1 Preschools Removed From the Analysis

From 2011 to 2016, there were total 10 080 school-level outbreaks, of which 105 led to closure. 9903 outbreaks including 100 closures were included in our model. A total of 177 schools were removed from the complete list, of which 175 did not contain information on school size, while 2 had inaccurate school size information (size 5 and 7 respectively, both smaller than outbreak size). Out of these 177 schools, one school was closed due to outbreak in 2012. Among all schools with closure, four schools had closures falling beyond 50 days after the first day of outbreak (51, 69, 56, and 52 days specifically). These four schools were categorized as having no closure in our analyses.

2 Statistical Models

Statistical models used in our analyses and the prior distributions used are presented in detail here.

2.1 Public Holiday Effect Model

7 days before and after model.

Let x_i and y_i be the number of cases before and after the PH, respectively, with

$$x_i \sim Poisson(\alpha_i \times 7\mu) \text{ and}$$

$$y_i \sim Poisson(\alpha_i \times [6\mu + \theta\mu]) \text{ or}$$

$$y_i \sim Poisson(\alpha_i \times [5\mu + 2\theta\mu]) \text{ for two day long PHs.}$$

Here μ represents the average number of cases on a typical day, θ is the public holiday effect, and $\alpha_i \sim \Gamma(a, a)$ is an individual week effect which has an expected value of 1 and allows for autocorrelation in the time series.

7-day window before and second week after PHs model.

Let x_i and y_i be the number of cases one week before and in the second week after the PH (i.e. days 8 to 14), respectively. Notations and model are otherwise the same as the 7 days before and after model.

7-day window before and third week after PHs model.

Again, let x_i and y_i be the number of cases one week before and in the third week after the PH (i.e. days 15 to 21), respectively. Same model and notations are used as previous models.

Non-informative priors were adopted and summarized in the table below.

Parameter	Notation	Prior Density
Individual week parameter	α_i	$\Gamma(a, a)$

Individual week dispersion	a	$U(0,100)$
Daily cases parameter	μ	$U(0,100)$
Public holiday parameter	θ	$U(0,100)$

2.2 School Vacation Effect Model

Let Y_t be the number of HFMD cases observed in week t. We assume Y_t follows a negative binomial distribution with mean

$$\mu_t = (a + b \times Y_{t-1}) \times d^{H_{t-1}}$$

and shape parameter p, where $H_t = 1$ if week t is a school vacation and 0 otherwise, and a, b, d, p are model parameters that needed to be estimated: a accounts for background number, b accounts for autocorrelation while d determines the effect of school vacation.

Non-informative priors were adopted and summarized in the table below.

Parameter	Notation	Prior Density
Baseline parameter	a	$U(0,100)$
Autocorrelation parameter	b	$U\left(0,100\right)$
School vacation parameter	d	$U\left(0,100\right)$
Shape parameter	p	$U(0,1)$

2.3 School Closure during Outbreak Effect Model

Let $x_{i,j}$ be the number of incident cases on day j in outbreak i, which is assumed to follow a Poisson distribution:

$$x_{i,j} \sim Poisson(\lambda_{c_{i,j}} \times s_{i,j-1} \times p^{w_{i,j-1}})$$

where $c_{i,j}$ is the cumulative number of cases within 1 week prior to day j in outbreak i; $s_{i,j}$ is the number of susceptible children in the centre on day j in outbreak i; and $w_{i,j}$ is the indicator for school closure on day j in outbreak i ($w_{i,j}=1$ if the school were closed on day j in outbreak i, and 0 otherwise).

The effect of outbreak size was smoothed using the formula

$$\log(\lambda_k) \sim N\left(\log(\lambda_{k-1}),\sigma^2\right).$$

Non-informative priors were adopted and summarized in the table below.

Parameter	Notation	Prior density
School closure parameter	p	$U(0,100)$
Smoothing parameter	τ	$\Gamma(0.01,0.01)$

2.4 Estimation of Model Parameters

The posterior distribution was estimated using Markov chain Monte Carlo simulations, which was implemented using Just Another Gibbs Sampler (JAGS) within the R statistical environment using 10 000 iterations with a burn-in period of 1000 iterations using the *rjags* package.

Trace plots and density plots of the posterior samples were plotted to confirm adequate convergence of the posterior samples.

Posterior mean and 95% credible interval were calculated for our public holiday effect parameters.

All posterior samples were used in the simulations in school vacation and school closure during outbreak analysis.

Table S1. Dates of public holidays in Singapore. If a public holiday falls on Sunday, the following Monday will be a public holiday.

**Public holiday**	**Date**
New Year's Day	1 January
Chinese New Year	2 days in January or February
Good Friday	1 day in March or April
Labour Day	1 May
Vesak Day	1 day in May or June
Hari Raya Puasa	1 day all year round
National Day	9 August
Hari Raya Haji	1 day about three months after Hari Raya Puasa
Deepavali	1 day in October or November
Christmas Day	25 December

Figure S1. Temporally structured distribution of residuals in the one-week ahead model forecast with loess curve overlaid. Blue line shows temporally structured residuals and orange line shows the loess curve.

Figure S2. Total number of cases one week before public holidays and total number of cases the first week after the public holidays. The left panel shows actual data points; the right panel shows simulated number of cases based on model posterior parameter distribution.

Figure S3. Total number of cases one week before public holidays and total number of cases the second week after the public holidays. The left panel shows actual data points; the right panel shows simulated number of cases based on model posterior parameter distribution.

Figure S4. Total number of cases one week before public holidays and total number of cases the third week after the public holidays. The left panel shows actual data points; the right panel shows simulated number of cases based on model posterior parameter distribution.

Figure S5. Actual observed time series of HFMD cases and one week ahead forecast based on school vacation effect model posterior parameter distribution. Blue lines show the one week ahead forecasts based on posterior parameter distribution; orange line shows actual observed time series.
