# Supplementary material for: The Effect of School Closure on Hand, Foot, and Mouth Disease Transmission in Singapore: A Modeling Approach
Source: Am J Trop Med Hyg. 2018 Oct 22;99(6):1625–32. doi: 10.4269/ajtmh.18-0099 (PMC6283473; doi:10.4269/ajtmh.18-0099)
Supplement: Supplementary file 2 [file tpmd180099.SD2.pdf]

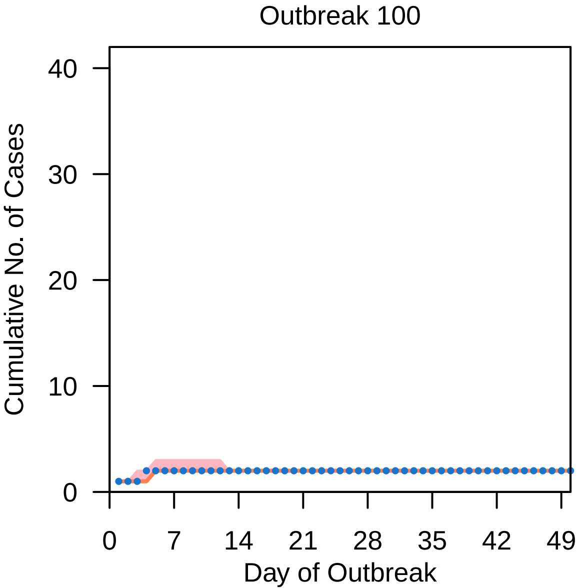

Outbreak 100
Cumulative No. of Cases
0
10
20
30
40
0
7
14
21
28
35
42
49
Day of Outbreak

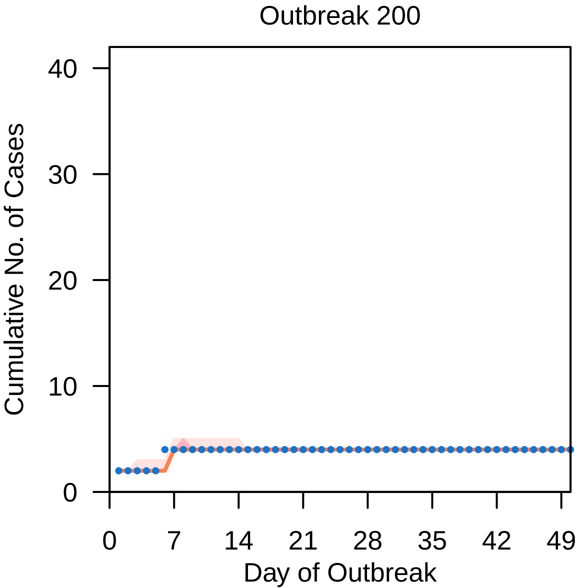

Outbreak 200
Cumulative No. of Cases
0
10
20
30
40
0
7
14
21
28
35
42
49
Day of Outbreak

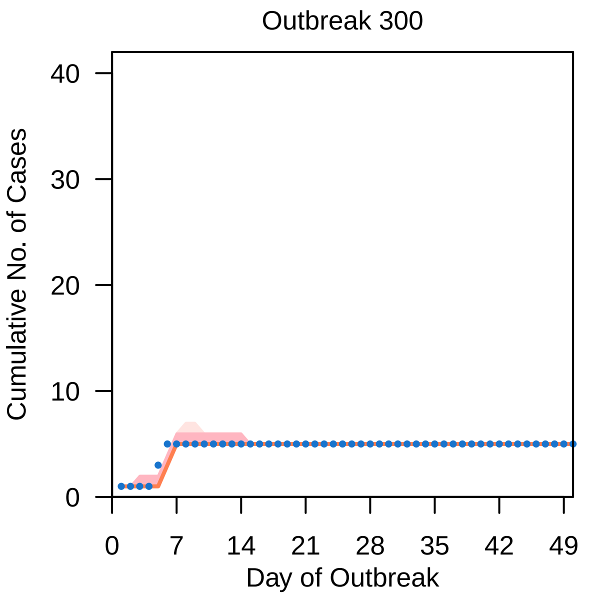

Outbreak 300
Cumulative No. of Cases
0
10
20
30
40
0
7
14
21
28
35
42
49
Day of Outbreak

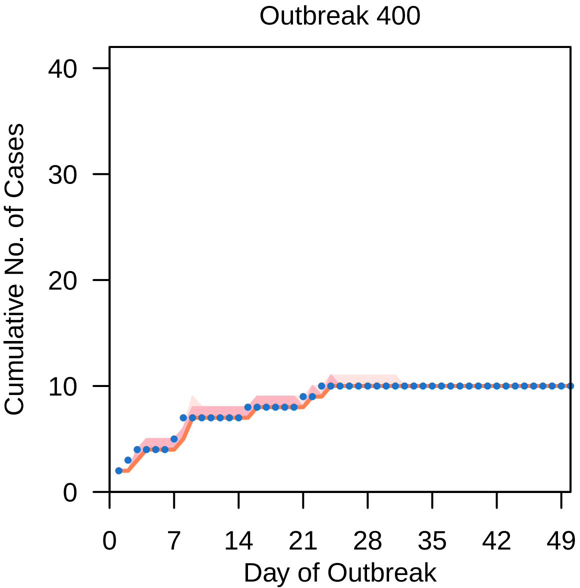
Outbreak 400
Cumulative No. of Cases
Day of Outbreak
0
10
20
30
40
0
7
14
21
28
35
42
49

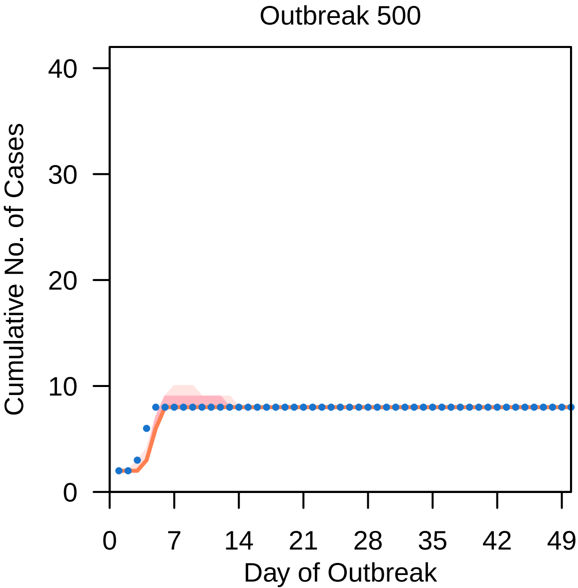

Outbreak 500
Cumulative No. of Cases
0
10
20
30
40
0
7
14
21
28
35
42
49
Day of Outbreak

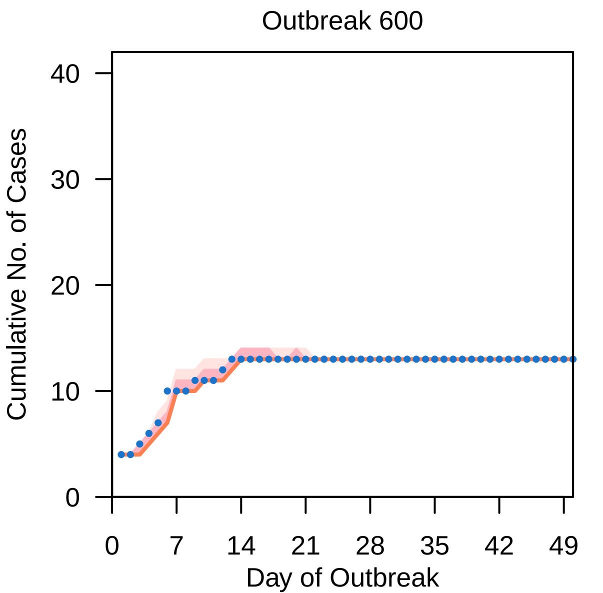
Outbreak 600
Cumulative No. of Cases
0
10
20
30
40
0
7
14
21
28
35
42
49
Day of Outbreak

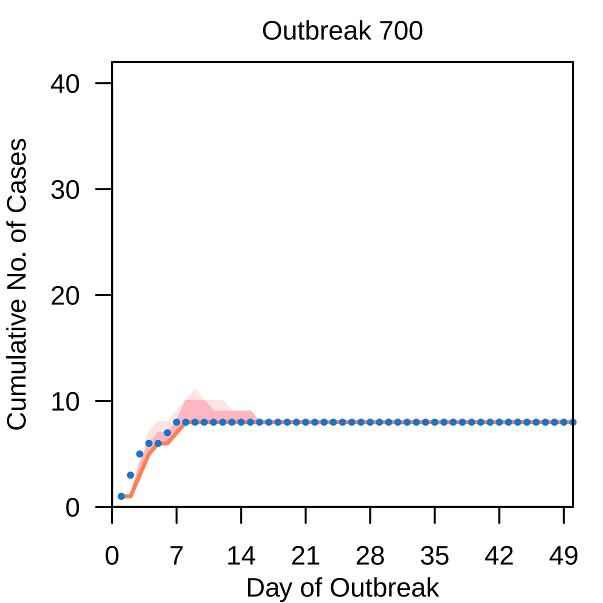

Outbreak 700
Cumulative No. of Cases
Day of Outbreak
0
10
20
30
40
0
7
14
21
28
35
42
49

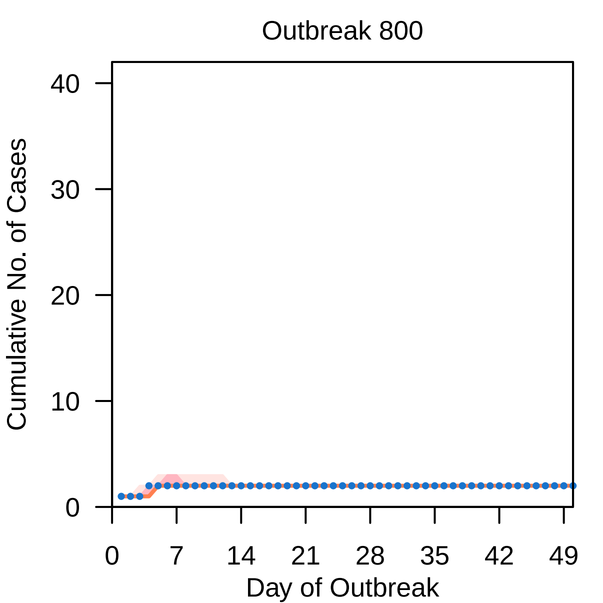
Outbreak 800
Cumulative No. of Cases
0
10
20
30
40
0
7
14
21
28
35
42
49
Day of Outbreak

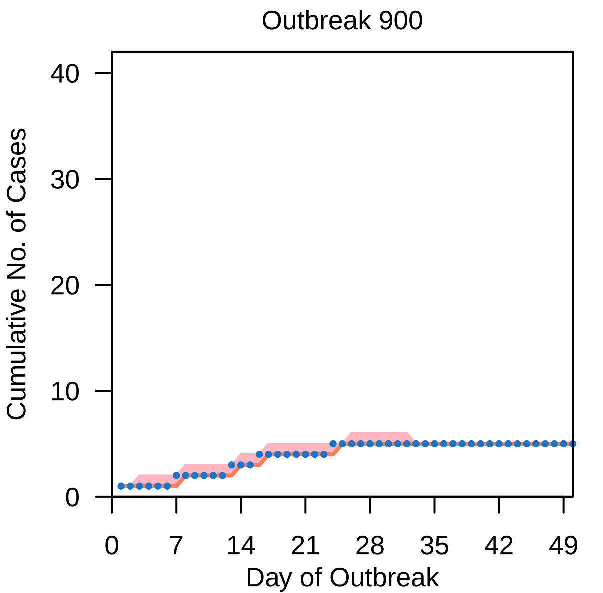

Outbreak 900
Cumulative No. of Cases
Day of Outbreak
0
10
20
30
40
0
7
14
21
28
35
42
49

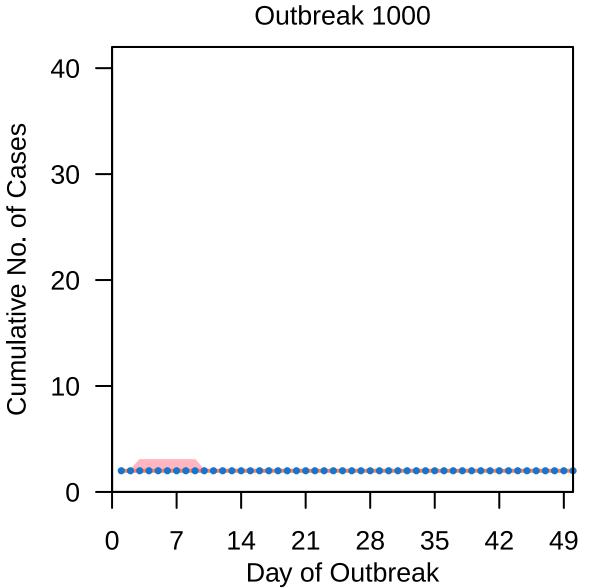

Outbreak 1000
Cumulative No. of Cases
0
10
20
30
40
0
7
14
21
28
35
42
49
Day of Outbreak

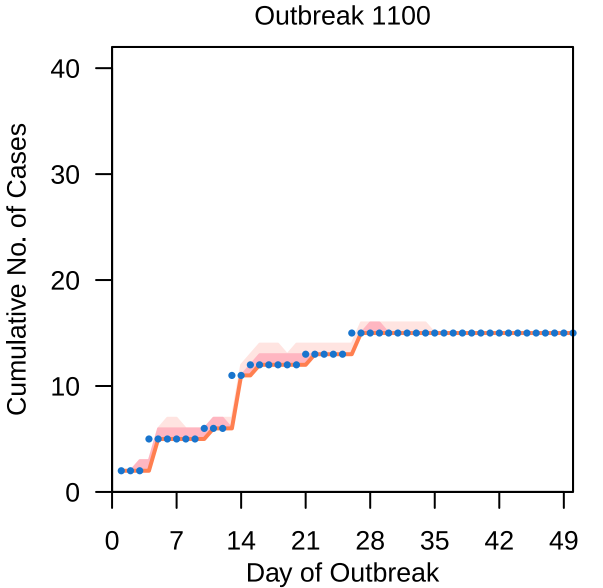

Outbreak 1100
Cumulative No. of Cases
0
10
20
30
40
0
7
14
21
28
35
42
49
Day of Outbreak

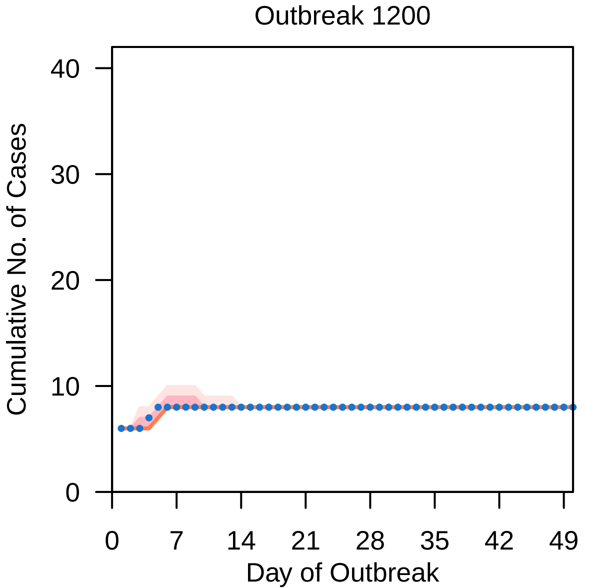
Outbreak 1200
Cumulative No. of Cases
0
10
20
30
40
0
7
14
21
28
35
42
49
Day of Outbreak

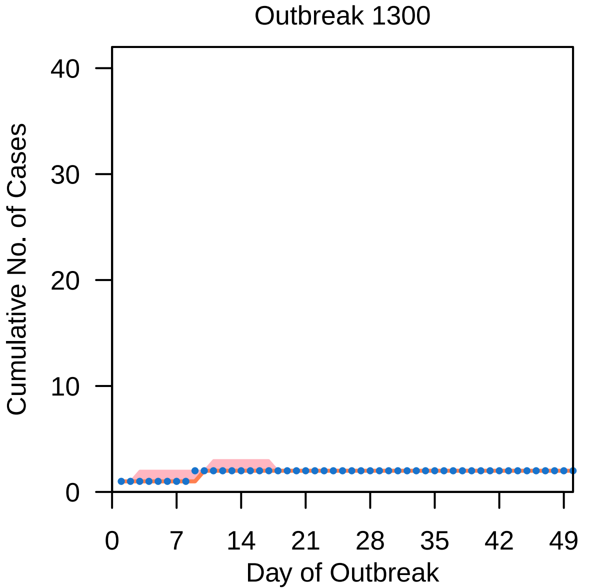

Outbreak 1300
Cumulative No. of Cases
0
10
20
30
40
0
7
14
21
28
35
42
49
Day of Outbreak

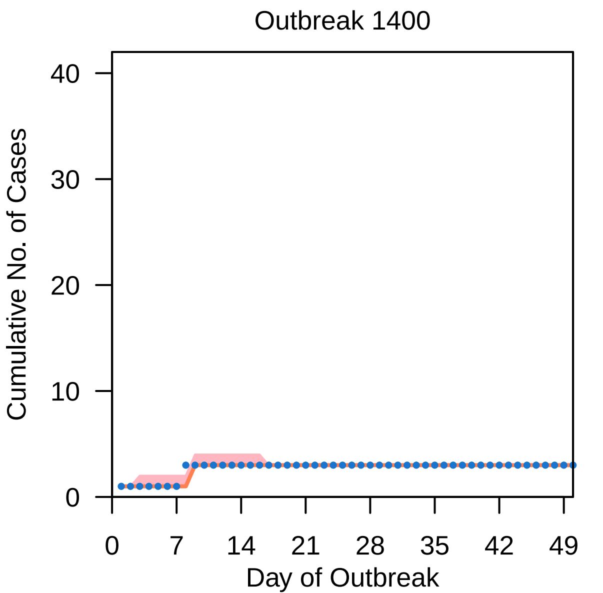

Outbreak 1400
Cumulative No. of Cases
0
10
20
30
40
0
7
14
21
28
35
42
49
Day of Outbreak

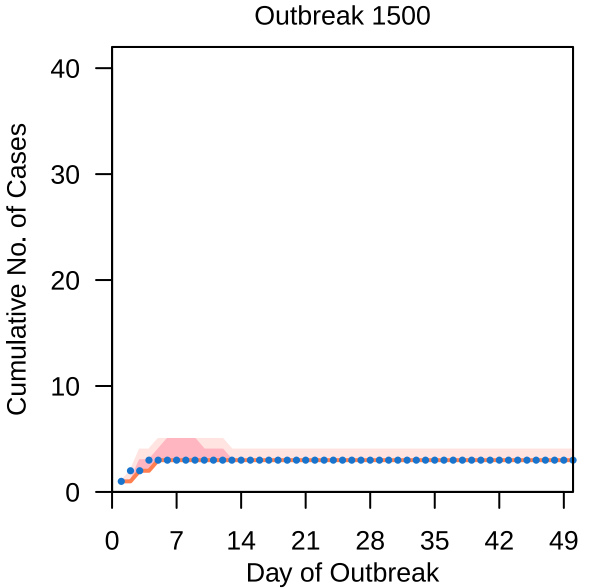

Outbreak 1500
Cumulative No. of Cases
0
10
20
30
40
Day of Outbreak
0
7
14
21
28
35
42
49

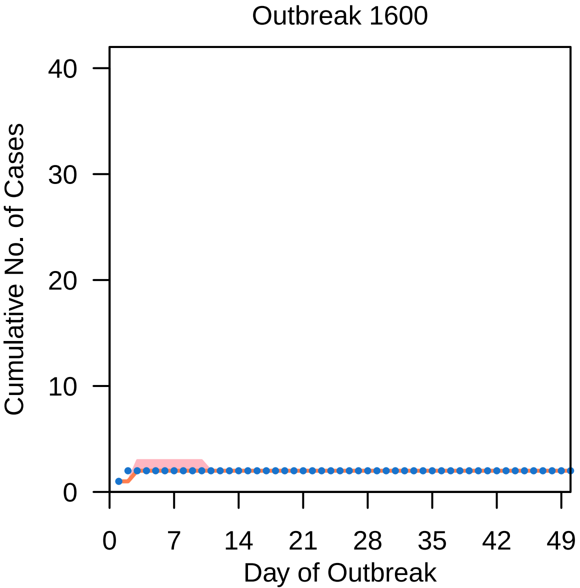

Outbreak 1600
Cumulative No. of Cases
0
10
20
30
40
0
7
14
21
28
35
42
49
Day of Outbreak

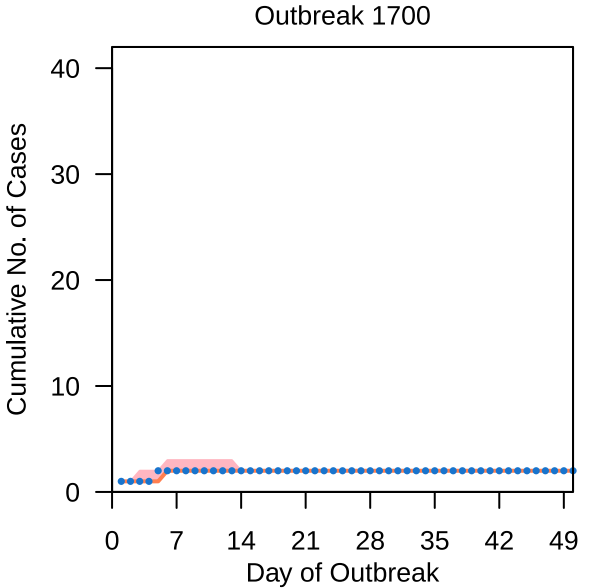

Outbreak 1700
Cumulative No. of Cases
Day of Outbreak
0
10
20
30
40
0
7
14
21
28
35
42
49

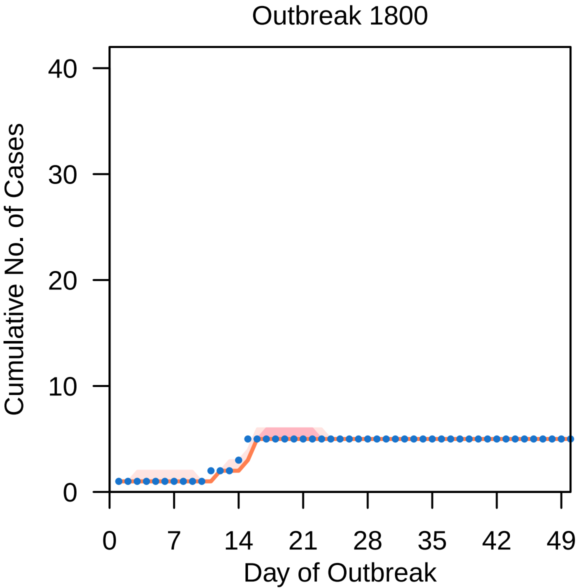

Outbreak 1800
Cumulative No. of Cases
Day of Outbreak
0
10
20
30
40
0
7
14
21
28
35
42
49

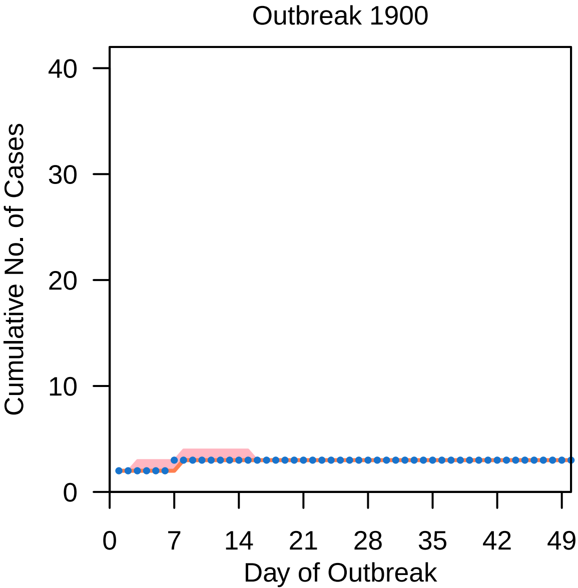
Outbreak 1900
Cumulative No. of Cases
0
10
20
30
40
0
7
14
21
28
35
42
49
Day of Outbreak

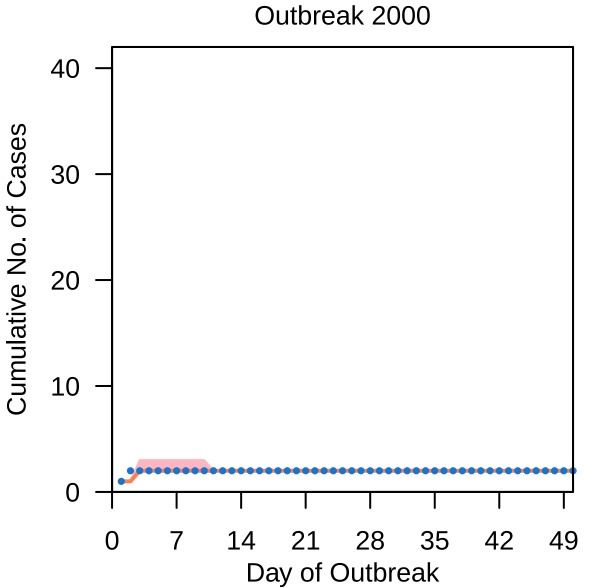

Outbreak 2000
Cumulative No. of Cases
0
10
20
30
40
0
7
14
21
28
35
42
49
Day of Outbreak

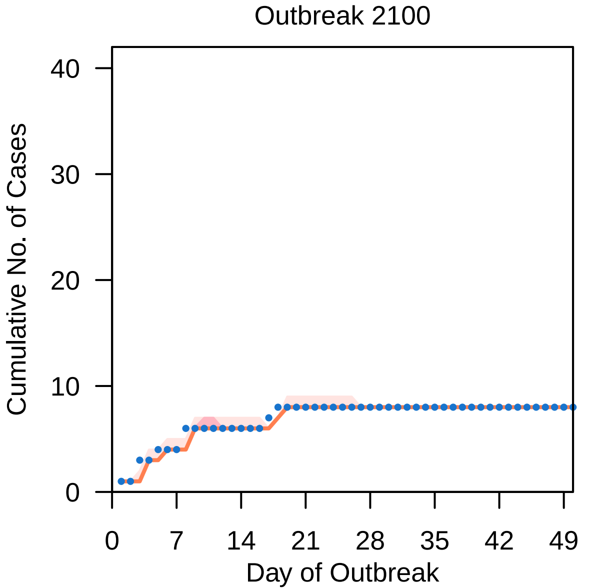
Outbreak 2100
Cumulative No. of Cases
Day of Outbreak
0
10
20
30
40
0
7
14
21
28
35
42
49

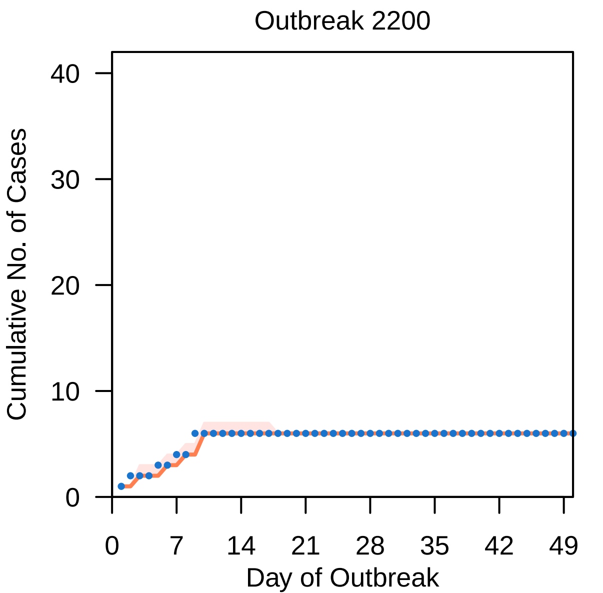

Outbreak 2200
Cumulative No. of Cases
0
10
20
30
40
0
7
14
21
28
35
42
49
Day of Outbreak

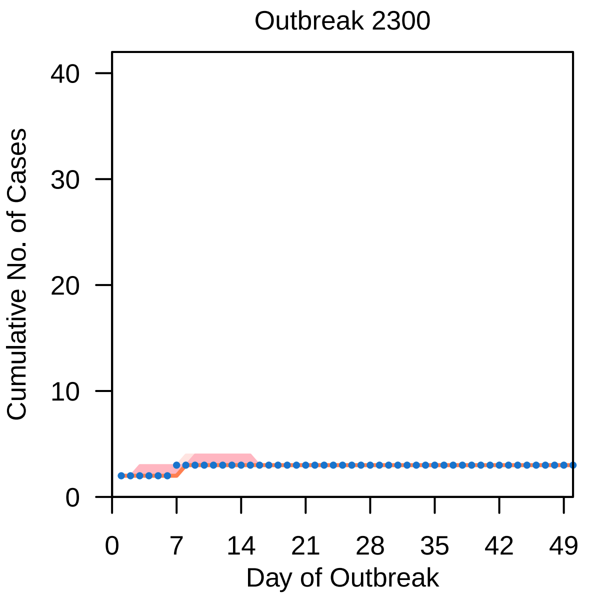

Outbreak 2300
Cumulative No. of Cases
0
10
20
30
40
0
7
14
21
28
35
42
49
Day of Outbreak

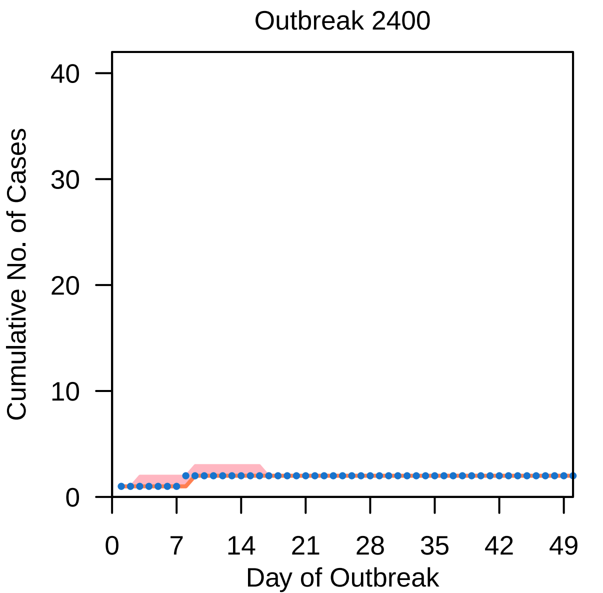

Outbreak 2400
Cumulative No. of Cases
40
30
20
10
0
0
7
14
21
28
35
42
49
Day of Outbreak

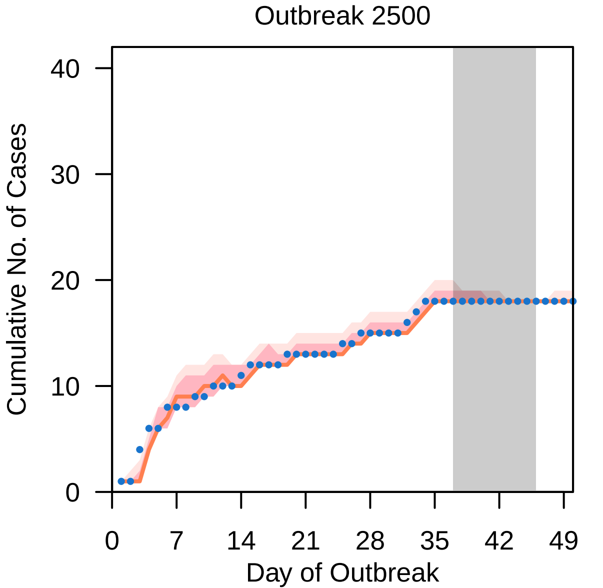

Outbreak 2500
Cumulative No. of Cases
Day of Outbreak
0
10
20
30
40
0
7
14
21
28
35
42
49

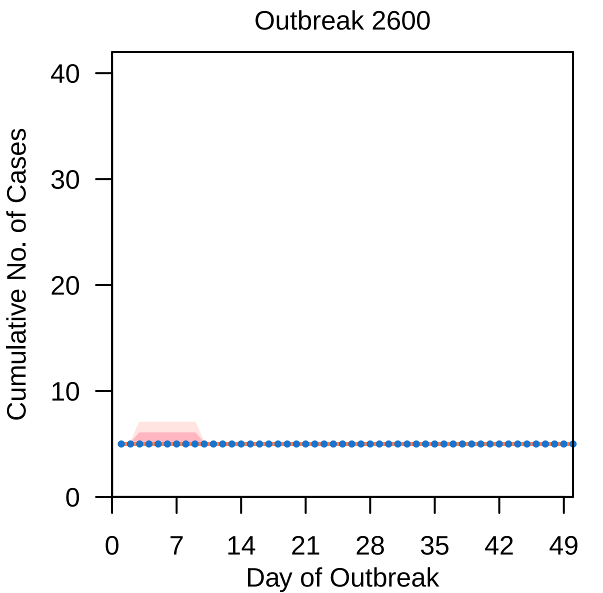
Outbreak 2600
Cumulative No. of Cases
0
10
20
30
40
0
7
14
21
28
35
42
49
Day of Outbreak

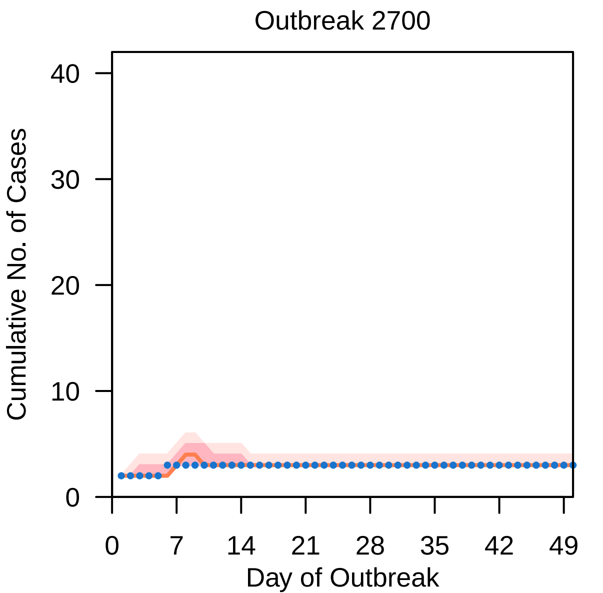
Outbreak 2700
Cumulative No. of Cases
0
10
20
30
40
0
7
14
21
28
35
42
49
Day of Outbreak

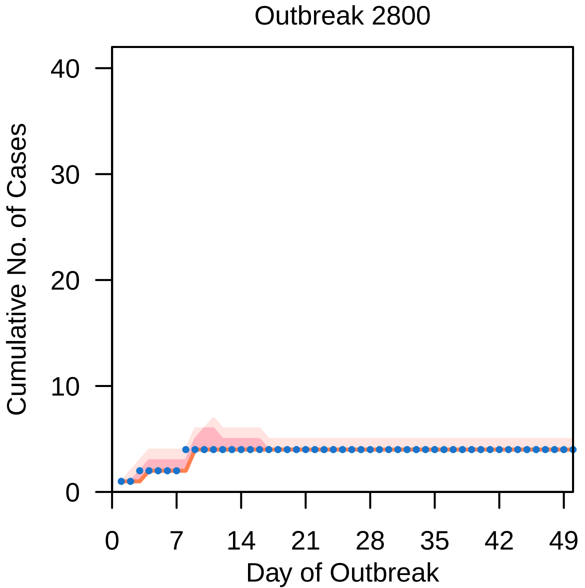
Outbreak 2800
Cumulative No. of Cases
Day of Outbreak
0
10
20
30
40
0
7
14
21
28
35
42
49

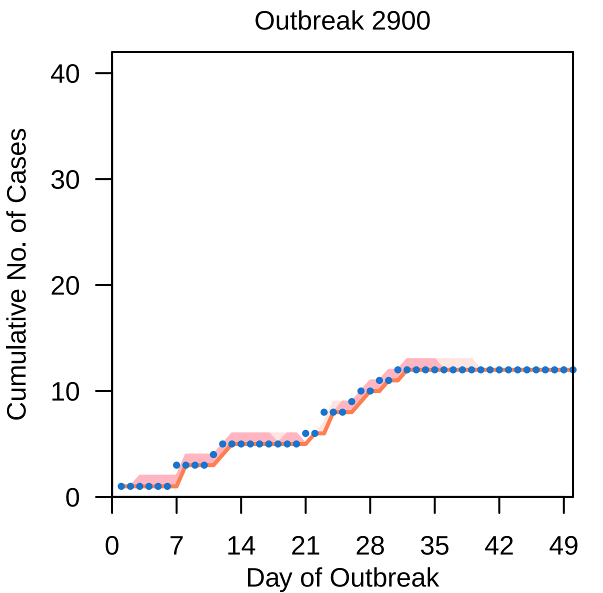

Outbreak 2900
Cumulative No. of Cases
40
30
20
10
0
0
7
14
21
28
35
42
49
Day of Outbreak

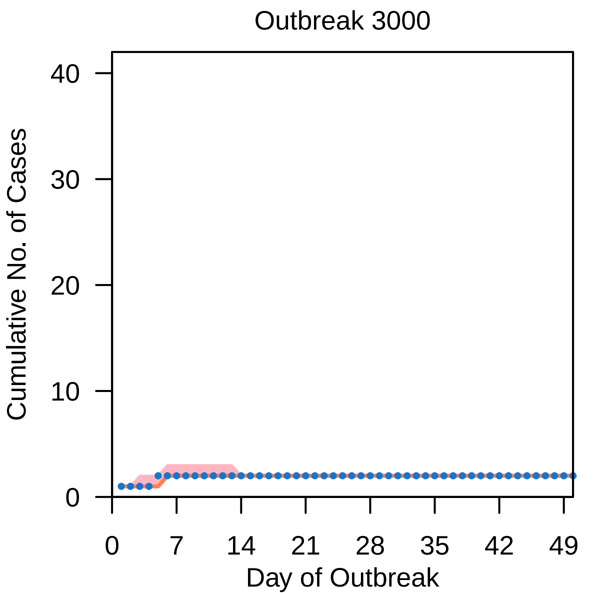
Outbreak 3000
Cumulative No. of Cases
0
10
20
30
40
0
7
14
21
28
35
42
49
Day of Outbreak

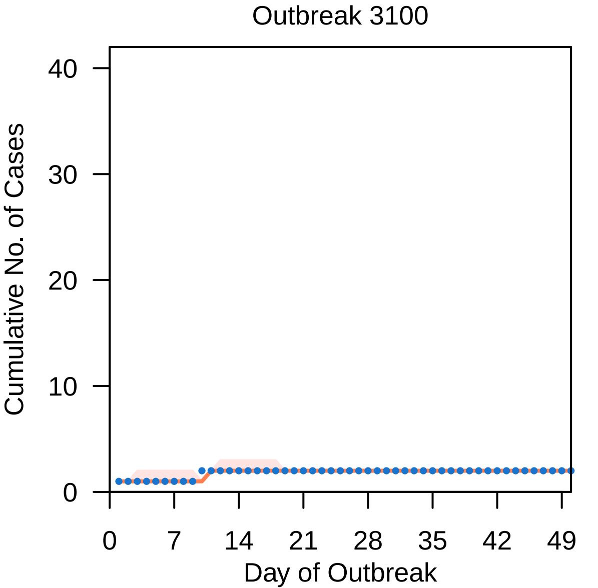

Outbreak 3100
Cumulative No. of Cases
Day of Outbreak
0
10
20
30
40
0
7
14
21
28
35
42
49

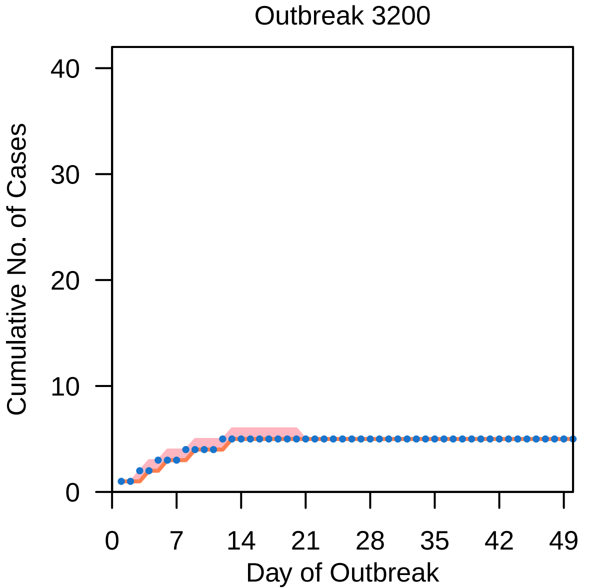
Outbreak 3200
Cumulative No. of Cases
0
10
20
30
40
0
7
14
21
28
35
42
49
Day of Outbreak

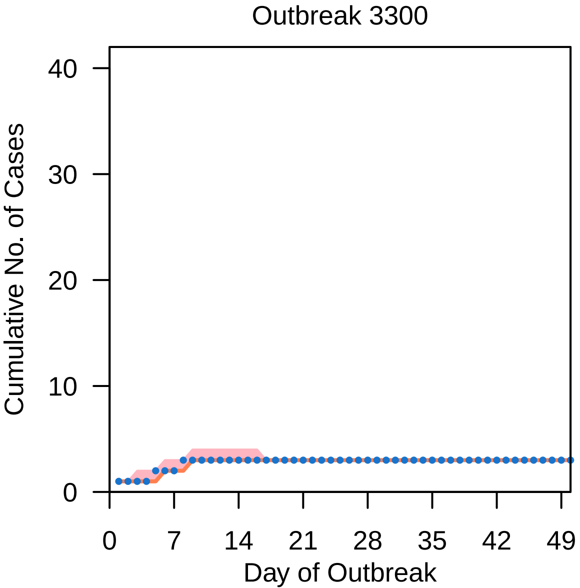
Outbreak 3300
Cumulative No. of Cases
0
10
20
30
40
0
7
14
21
28
35
42
49
Day of Outbreak

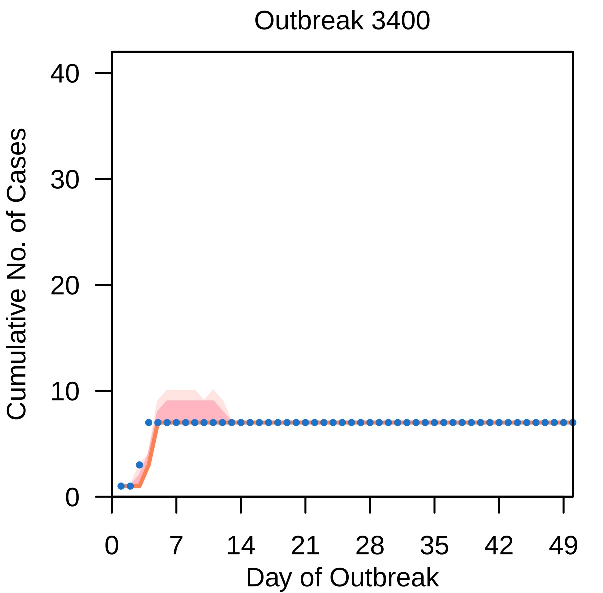

Outbreak 3400
Cumulative No. of Cases
Day of Outbreak
0
10
20
30
40
0
7
14
21
28
35
42
49

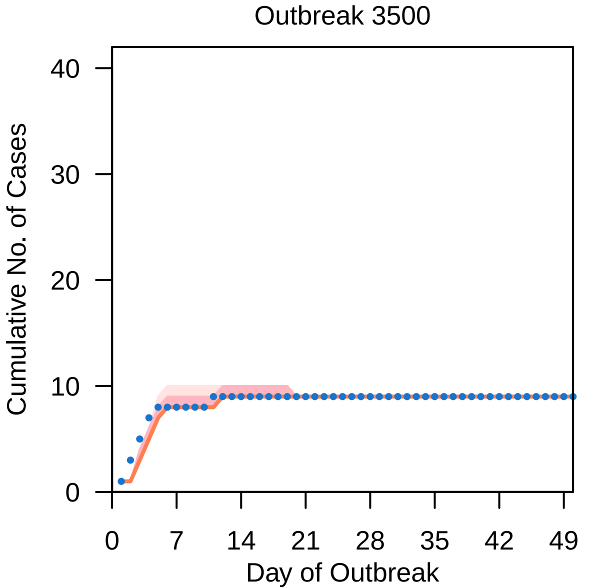

Outbreak 3500
Cumulative No. of Cases
0
10
20
30
40
0
7
14
21
28
35
42
49
Day of Outbreak

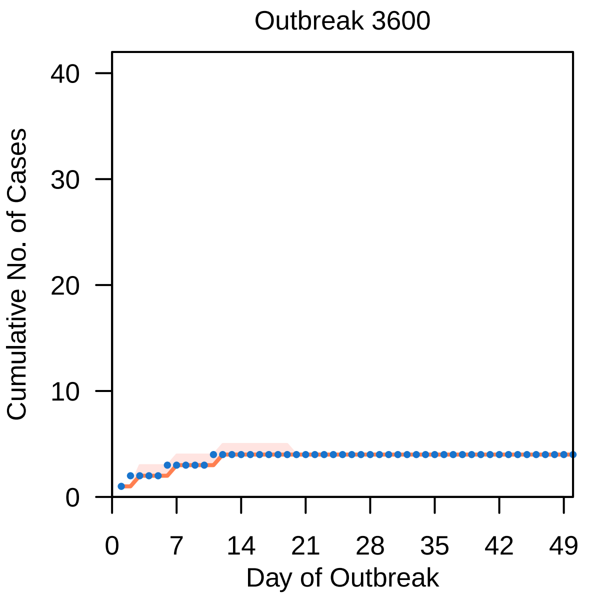

Outbreak 3600
Cumulative No. of Cases
Day of Outbreak
0
10
20
30
40
0
7
14
21
28
35
42
49

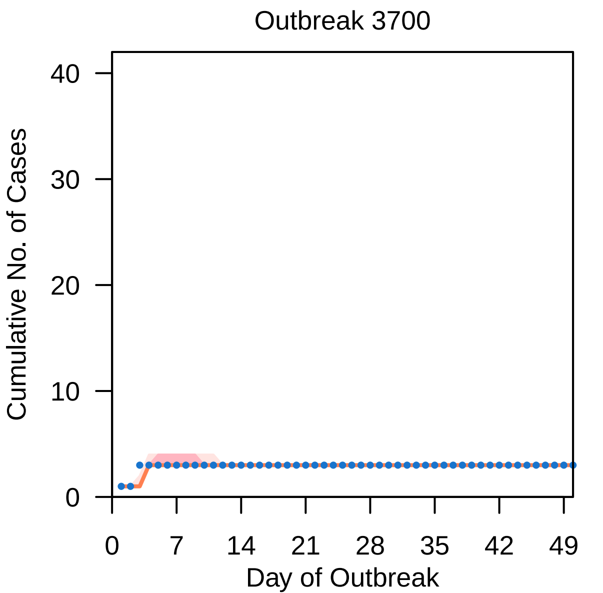

Outbreak 3700
Cumulative No. of Cases
0
10
20
30
40
0
7
14
21
28
35
42
49
Day of Outbreak

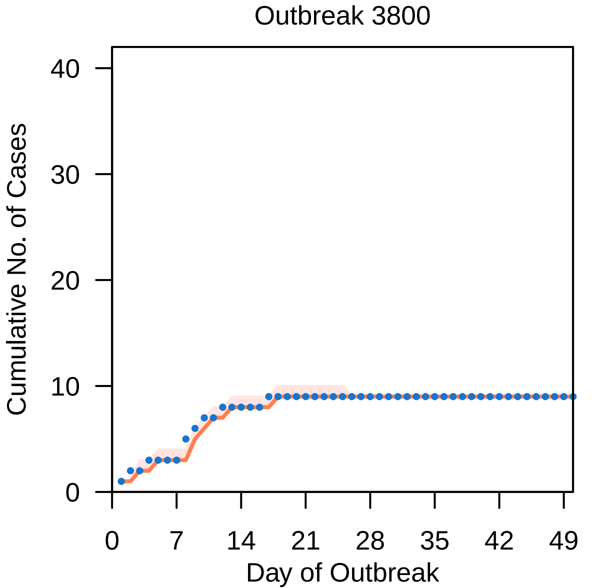

Outbreak 3800
Cumulative No. of Cases
0
10
20
30
40
0
7
14
21
28
35
42
49
Day of Outbreak

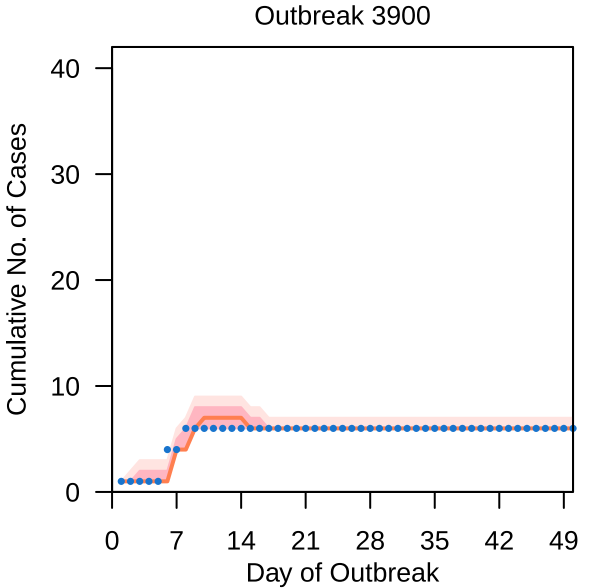

Outbreak 3900
Cumulative No. of Cases
0
10
20
30
40
0
7
14
21
28
35
42
49
Day of Outbreak

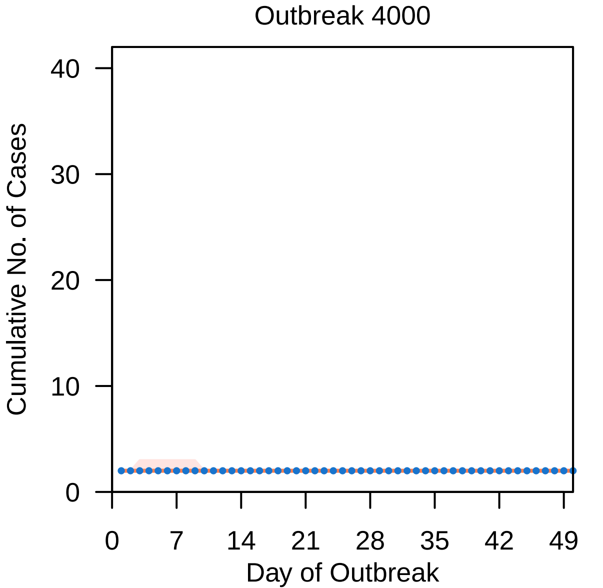

Outbreak 4000
Cumulative No. of Cases
0
10
20
30
40
0
7
14
21
28
35
42
49
Day of Outbreak

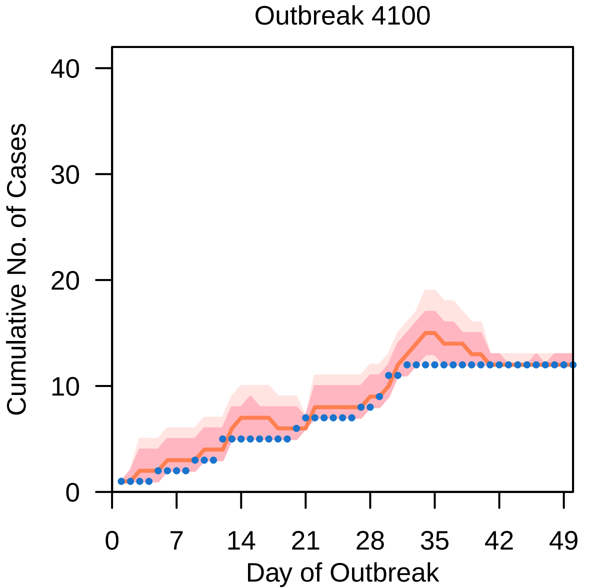
Outbreak 4100
Cumulative No. of Cases
40
30
20
10
0
0
7
14
21
28
35
42
49
Day of Outbreak

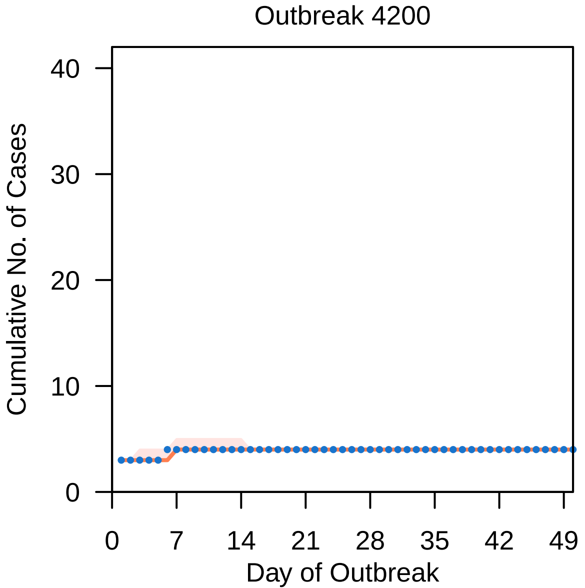
Outbreak 4200
Cumulative No. of Cases
Day of Outbreak
0
10
20
30
40
0
7
14
21
28
35
42
49

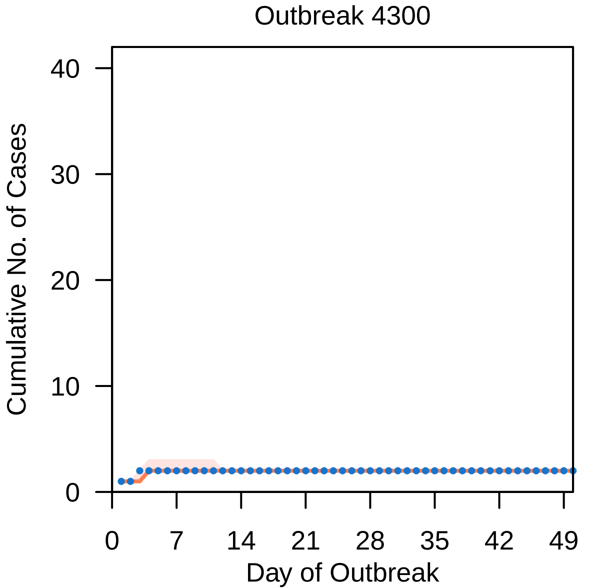
Outbreak 4300
Cumulative No. of Cases
0
10
20
30
40
0
7
14
21
28
35
42
49
Day of Outbreak

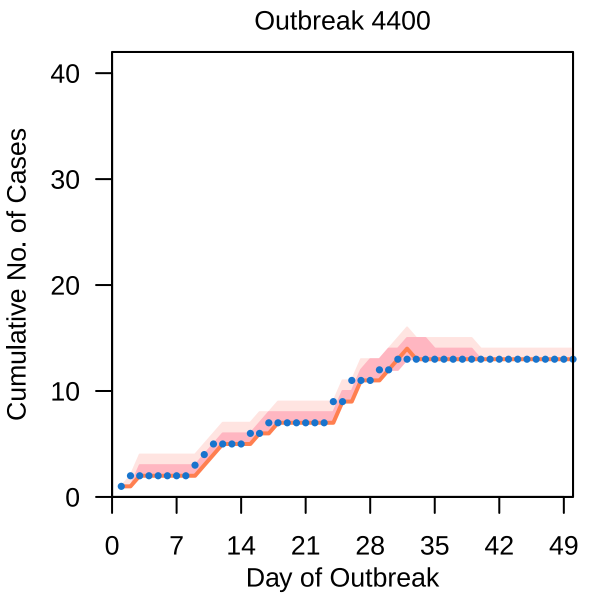

Outbreak 4400
Cumulative No. of Cases
0
10
20
30
40
0
7
14
21
28
35
42
49
Day of Outbreak

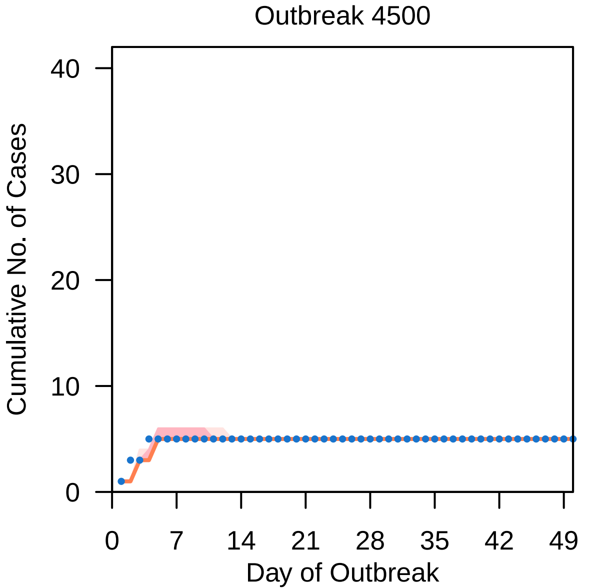
Outbreak 4500
Cumulative No. of Cases
0
10
20
30
40
0
7
14
21
28
35
42
49
Day of Outbreak

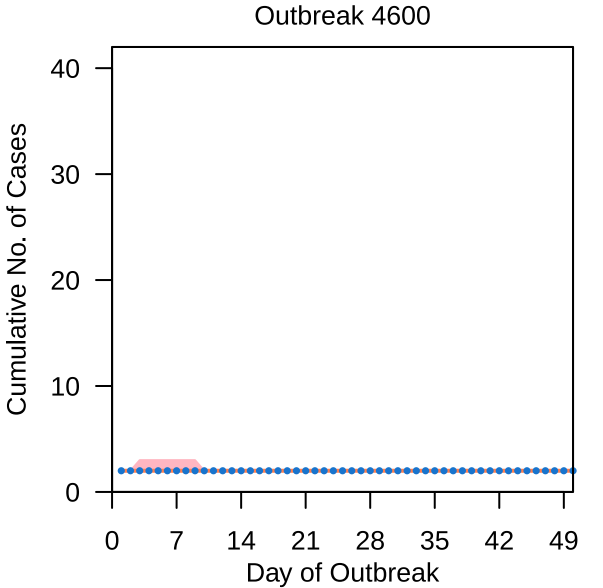

Outbreak 4600
Cumulative No. of Cases
0
10
20
30
40
0
7
14
21
28
35
42
49
Day of Outbreak

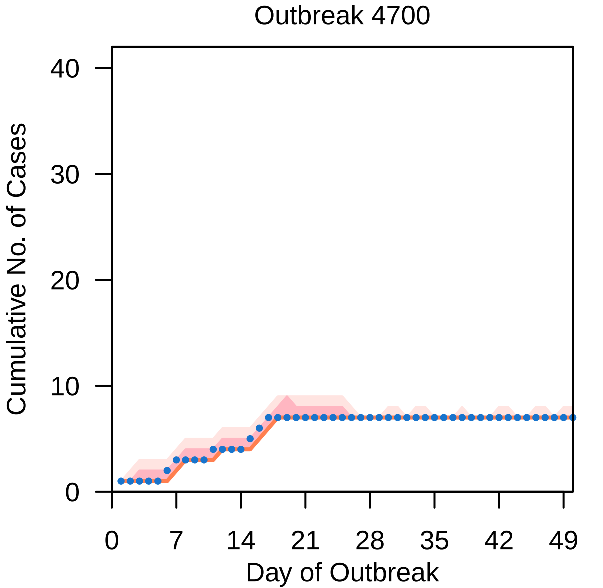

Outbreak 4700
Cumulative No. of Cases
0
10
20
30
40
0
7
14
21
28
35
42
49
Day of Outbreak

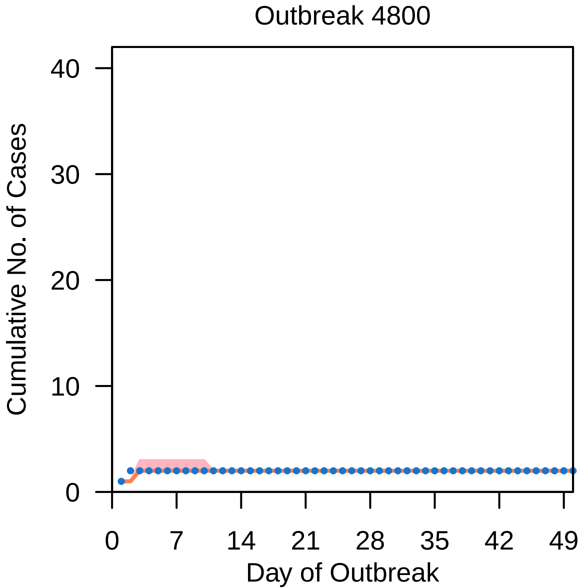

Outbreak 4800
Cumulative No. of Cases
0
10
20
30
40
0
7
14
21
28
35
42
49
Day of Outbreak

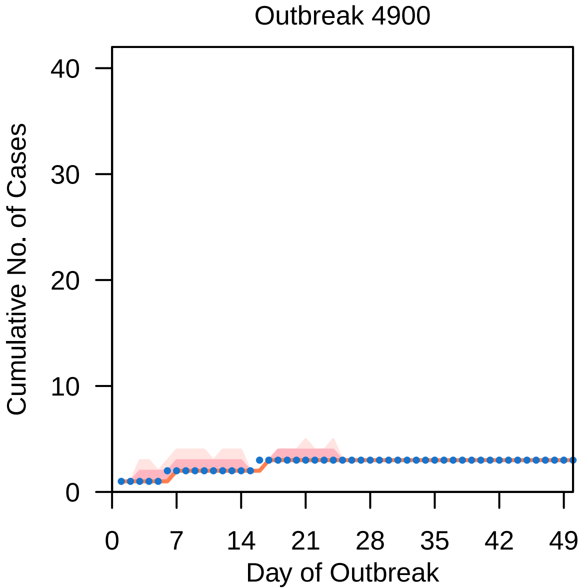
Outbreak 4900
Cumulative No. of Cases
Day of Outbreak
0
10
20
30
40
0
7
14
21
28
35
42
49

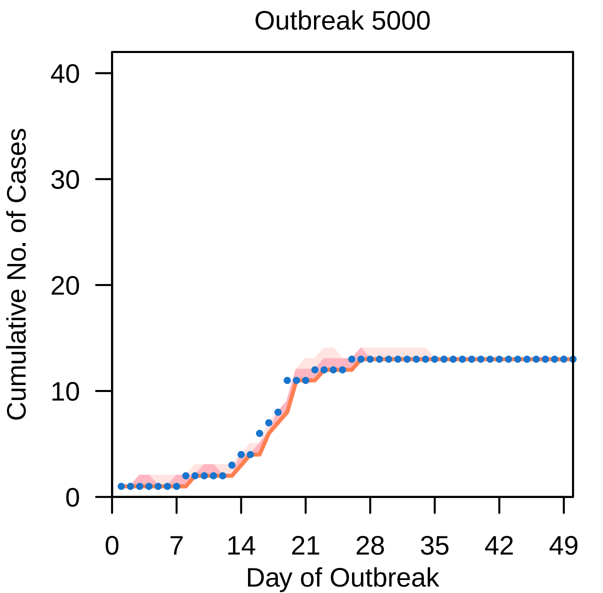
Outbreak 5000
Cumulative No. of Cases
0
10
20
30
40
0
7
14
21
28
35
42
49
Day of Outbreak

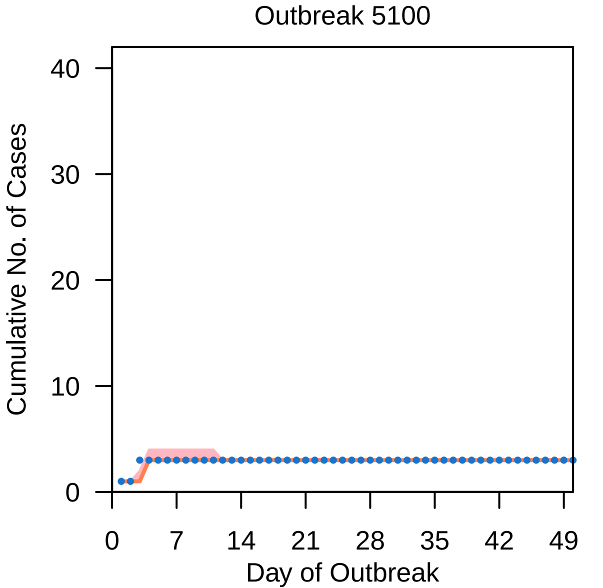

Outbreak 5100
Cumulative No. of Cases
Day of Outbreak
0
10
20
30
40
0
7
14
21
28
35
42
49

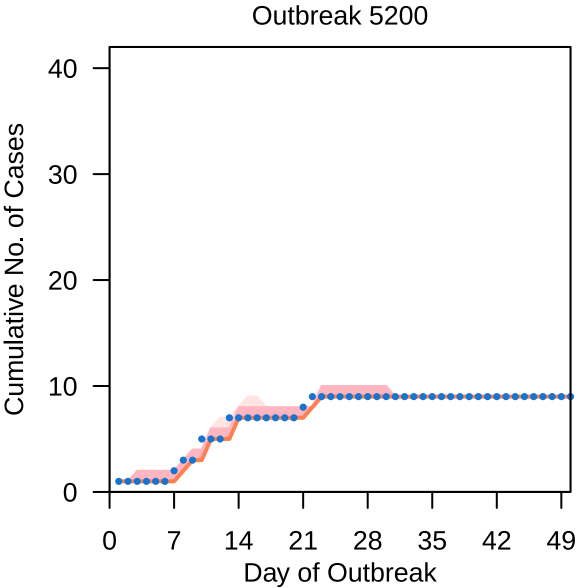

Outbreak 5200
Cumulative No. of Cases
0
10
20
30
40
0
7
14
21
28
35
42
49
Day of Outbreak

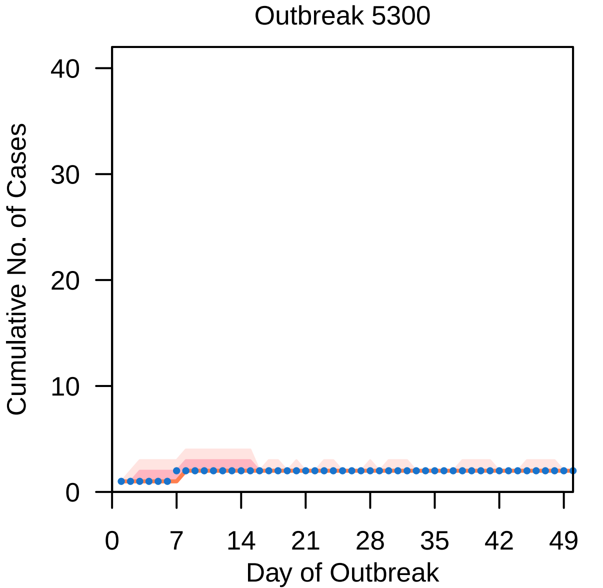
Outbreak 5300
Cumulative No. of Cases
Day of Outbreak
0
10
20
30
40
0
7
14
21
28
35
42
49

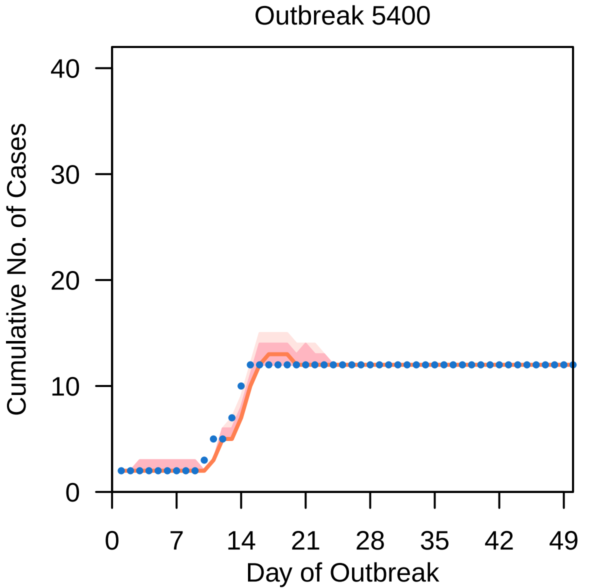
Outbreak 5400
Cumulative No. of Cases
0
10
20
30
40
0
7
14
21
28
35
42
49
Day of Outbreak

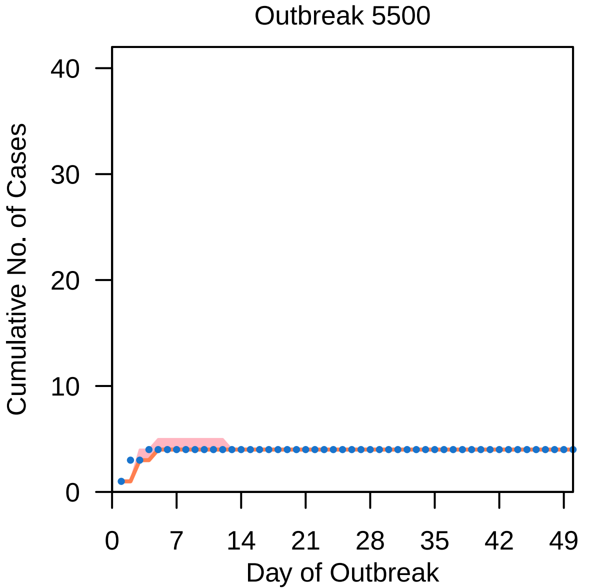
Outbreak 5500
Cumulative No. of Cases
0
10
20
30
40
0
7
14
21
28
35
42
49
Day of Outbreak

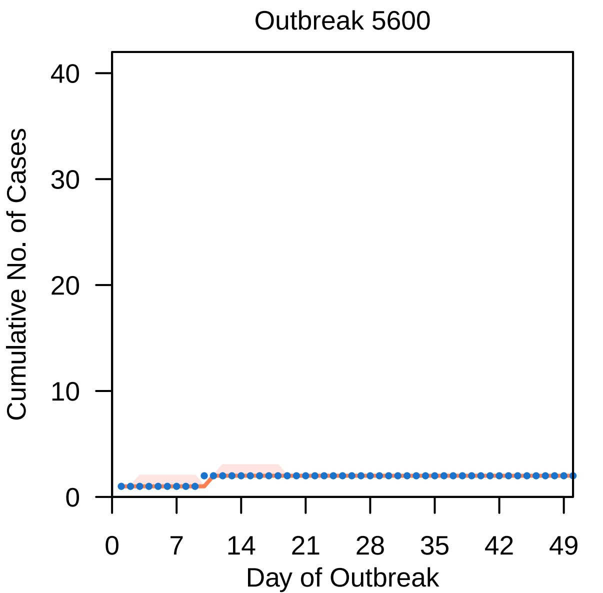
Outbreak 5600
Cumulative No. of Cases
0
10
20
30
40
0
7
14
21
28
35
42
49
Day of Outbreak

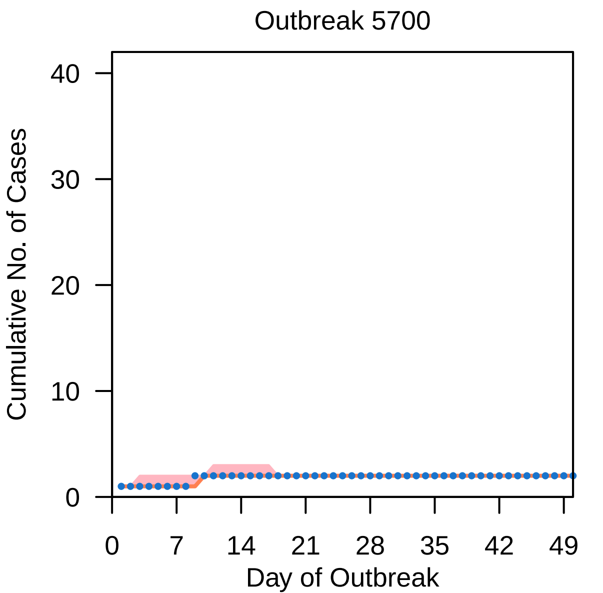

Outbreak 5700
Cumulative No. of Cases
0
10
20
30
40
0
7
14
21
28
35
42
49
Day of Outbreak

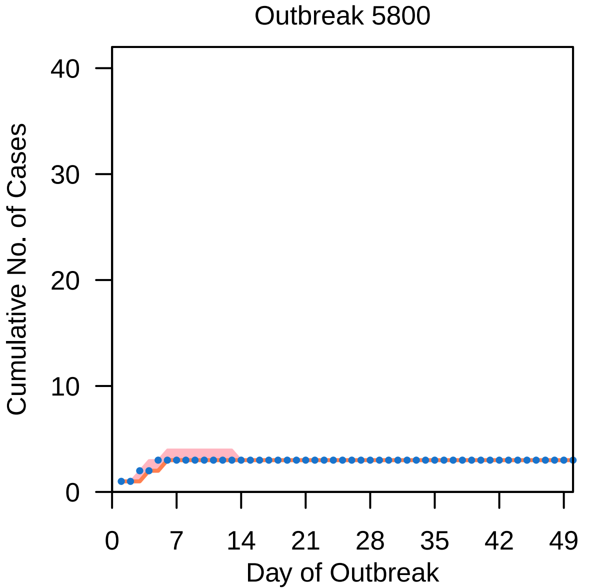

Outbreak 5800
Cumulative No. of Cases
40
30
20
10
0
0
7
14
21
28
35
42
49
Day of Outbreak

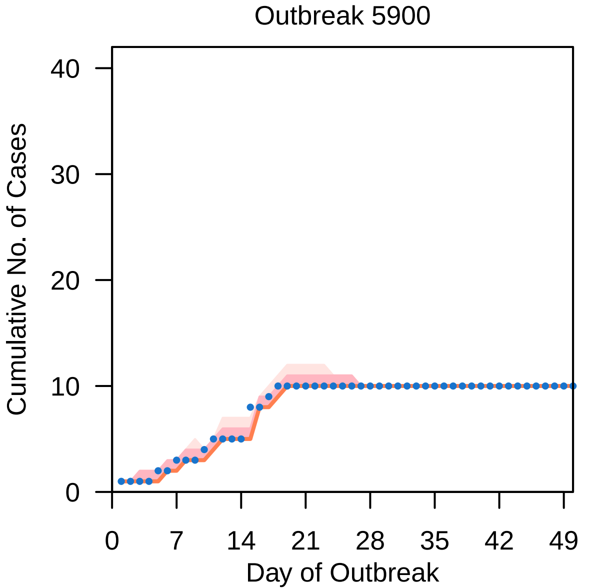

Outbreak 5900
Cumulative No. of Cases
0
10
20
30
40
0
7
14
21
28
35
42
49
Day of Outbreak

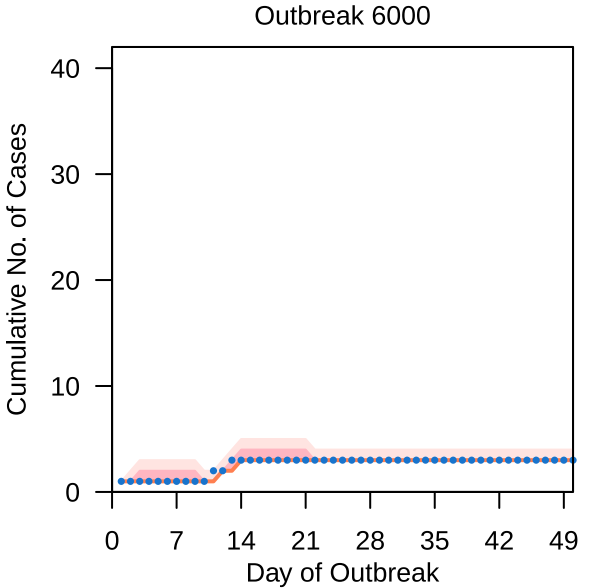

Outbreak 6000
Cumulative No. of Cases
0
10
20
30
40
0
7
14
21
28
35
42
49
Day of Outbreak

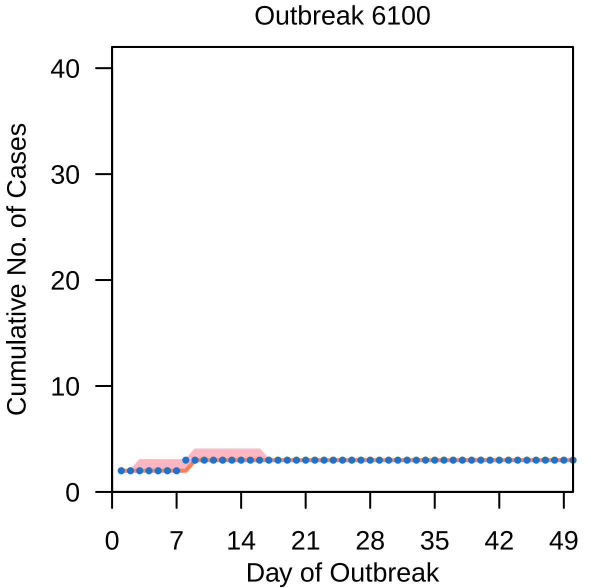

Outbreak 6100
Cumulative No. of Cases
0
10
20
30
40
0
7
14
21
28
35
42
49
Day of Outbreak

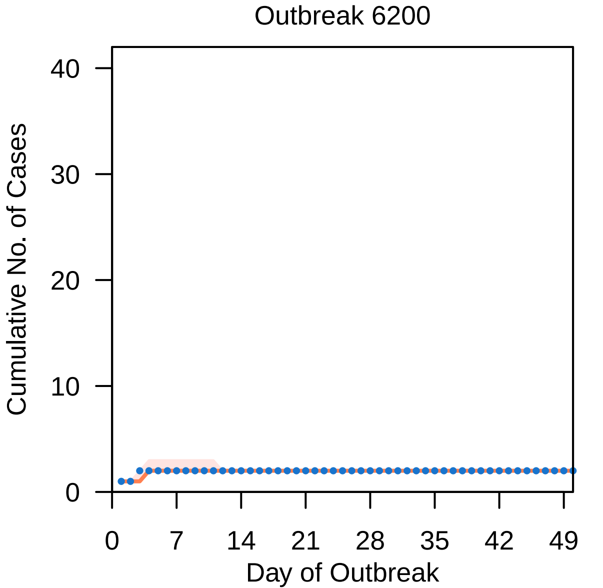

Outbreak 6200
Cumulative No. of Cases
0
10
20
30
40
0
7
14
21
28
35
42
49
Day of Outbreak

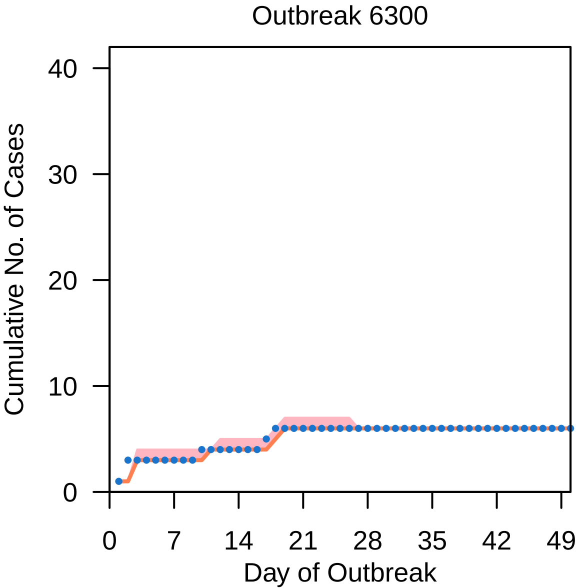
Outbreak 6300
Cumulative No. of Cases
Day of Outbreak
0
10
20
30
40
0
7
14
21
28
35
42
49

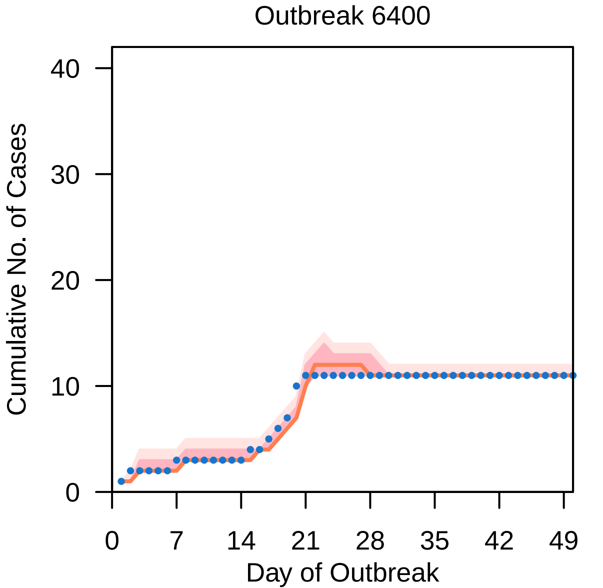

Outbreak 6400
Cumulative No. of Cases
0
10
20
30
40
0
7
14
21
28
35
42
49
Day of Outbreak

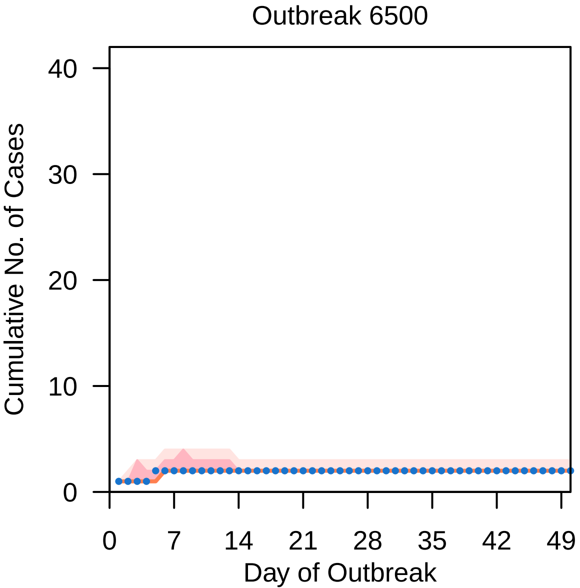
Outbreak 6500
Cumulative No. of Cases
Day of Outbreak
0
10
20
30
40
0
7
14
21
28
35
42
49

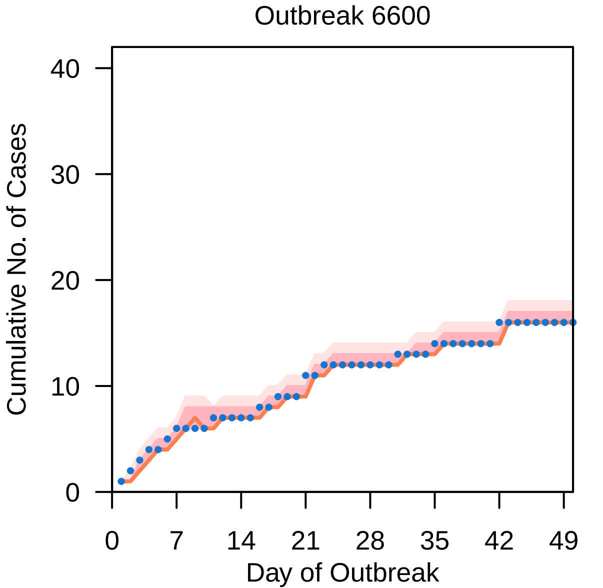
Outbreak 6600
Cumulative No. of Cases
0
10
20
30
40
Day of Outbreak
0
7
14
21
28
35
42
49

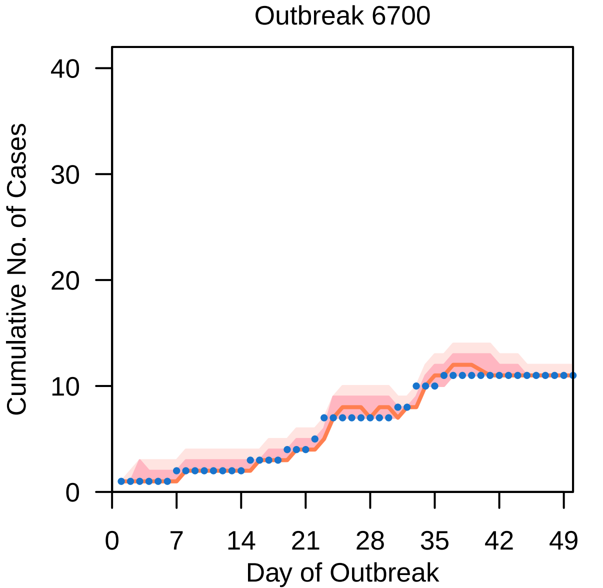

Outbreak 6700
Cumulative No. of Cases
40
30
20
10
0
0
7
14
21
28
35
42
49
Day of Outbreak

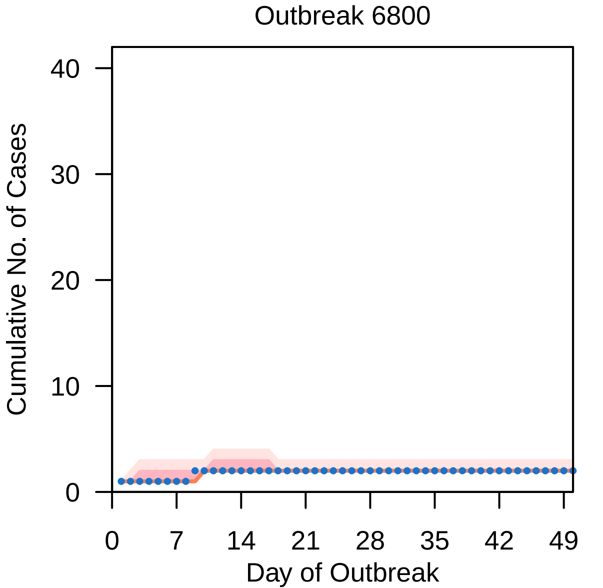
Outbreak 6800
Cumulative No. of Cases
0
10
20
30
40
0
7
14
21
28
35
42
49
Day of Outbreak

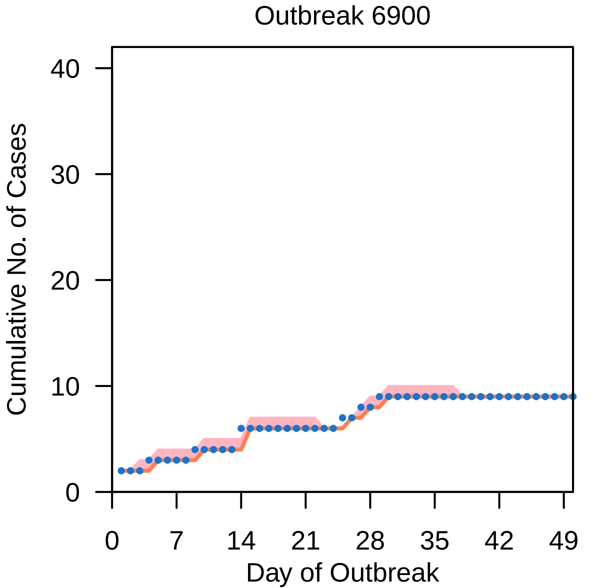
Outbreak 6900
Cumulative No. of Cases
0
10
20
30
40
0
7
14
21
28
35
42
49
Day of Outbreak

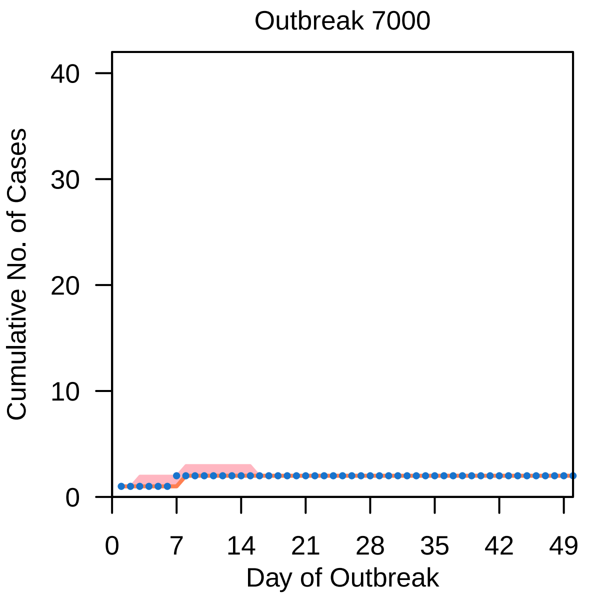

Outbreak 7000
Cumulative No. of Cases
0
10
20
30
40
0
7
14
21
28
35
42
49
Day of Outbreak

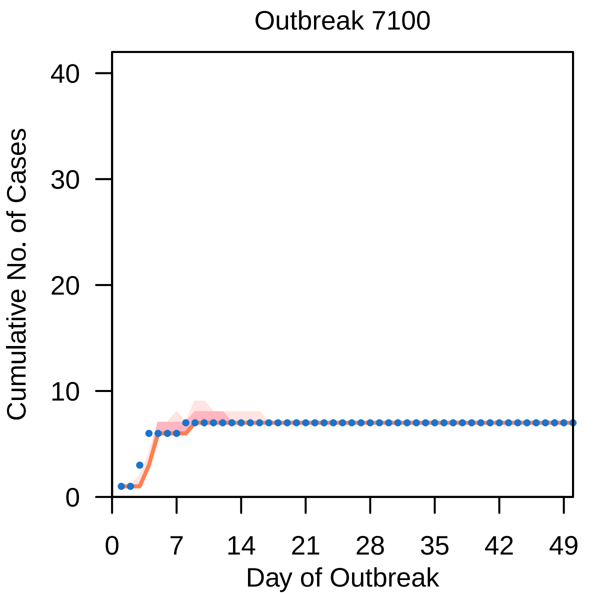
Outbreak 7100
Cumulative No. of Cases
0
10
20
30
40
0
7
14
21
28
35
42
49
Day of Outbreak

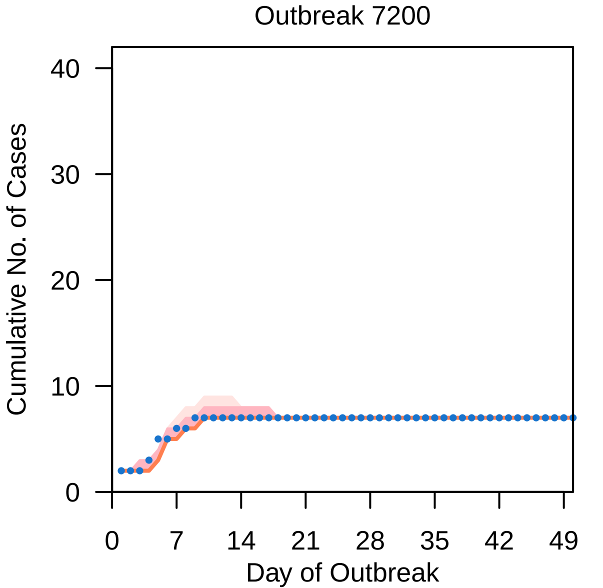
Outbreak 7200
Cumulative No. of Cases
Day of Outbreak
0
10
20
30
40
0
7
14
21
28
35
42
49

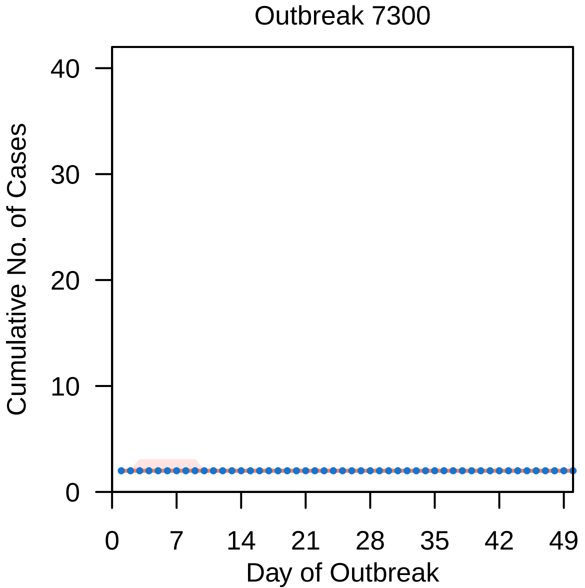

Outbreak 7300
Cumulative No. of Cases
0
10
20
30
40
0
7
14
21
28
35
42
49
Day of Outbreak

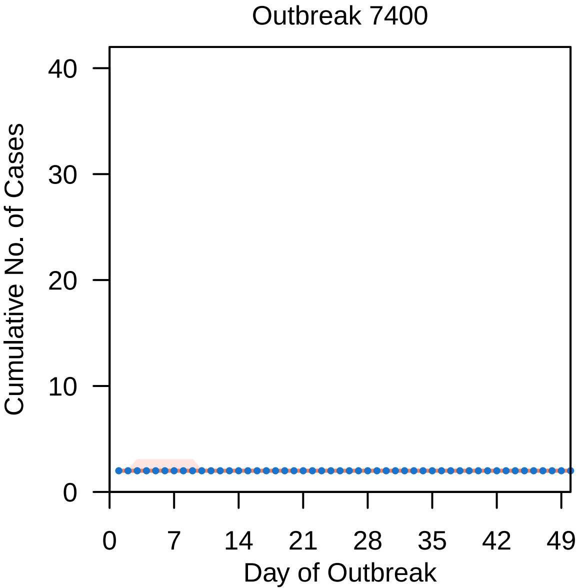

Outbreak 7400
Cumulative No. of Cases
Day of Outbreak
0
10
20
30
40
0
7
14
21
28
35
42
49

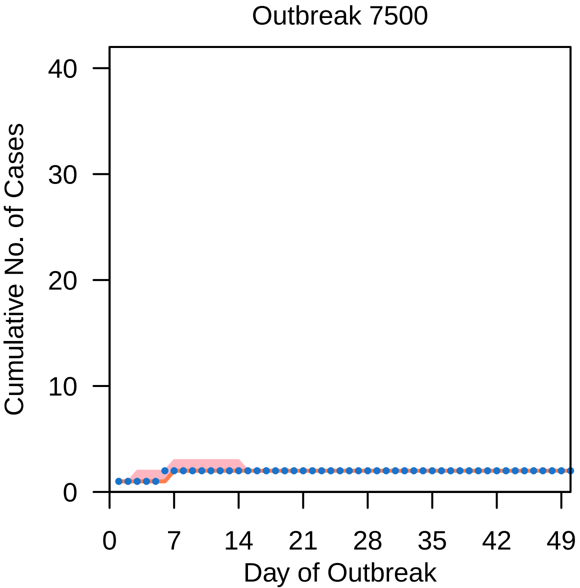

Outbreak 7500
Cumulative No. of Cases
Day of Outbreak
0
10
20
30
40
0
7
14
21
28
35
42
49

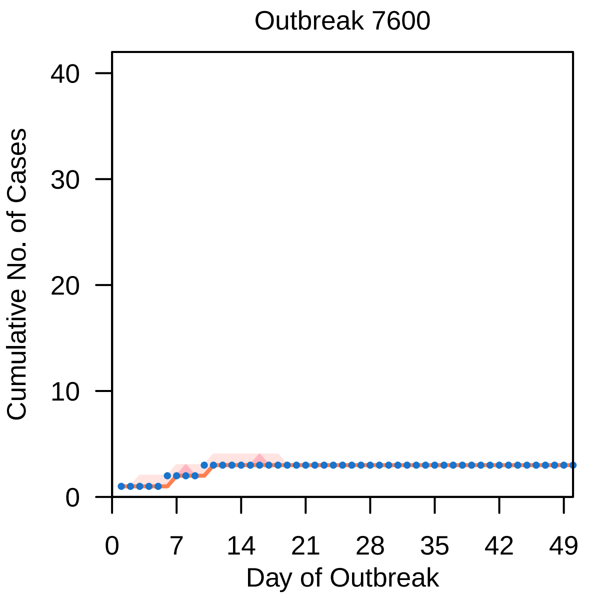

Outbreak 7600
Cumulative No. of Cases
Day of Outbreak
0
10
20
30
40
0
7
14
21
28
35
42
49

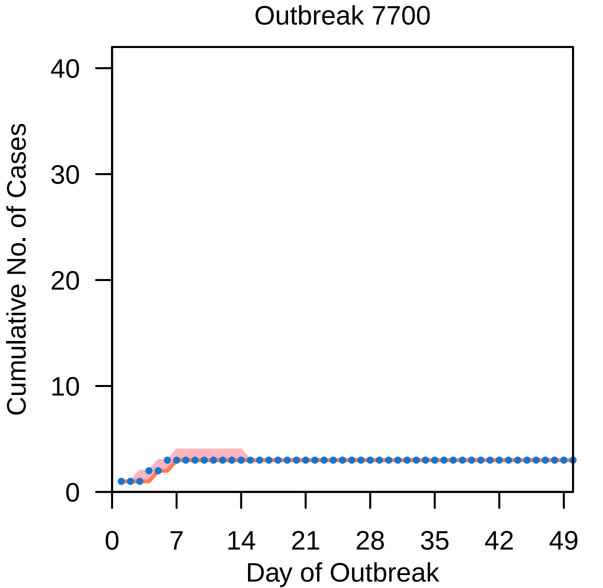
Outbreak 7700
Cumulative No. of Cases
0
10
20
30
40
0
7
14
21
28
35
42
49
Day of Outbreak

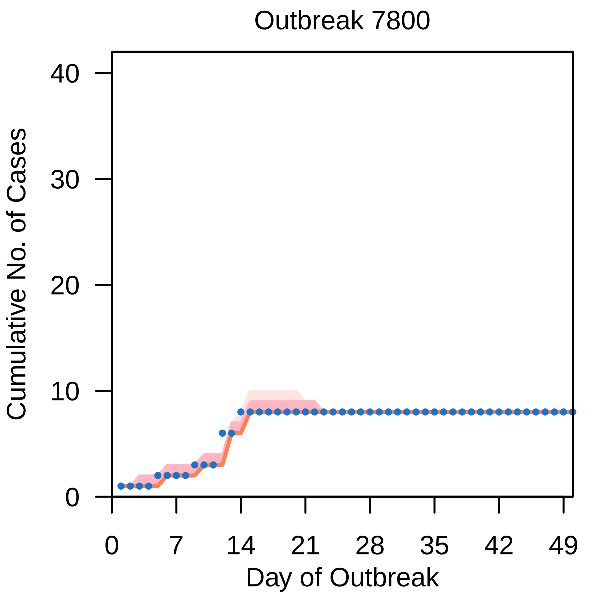

Outbreak 7800
Cumulative No. of Cases
Day of Outbreak
0
10
20
30
40
0
7
14
21
28
35
42
49

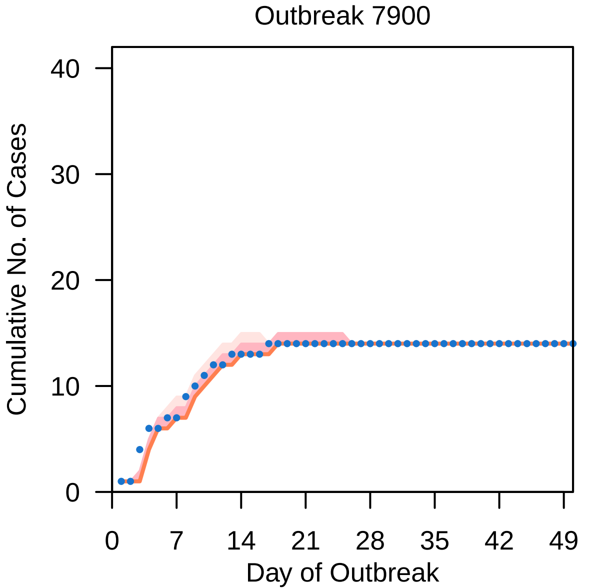

Outbreak 7900
Cumulative No. of Cases
0
10
20
30
40
0
7
14
21
28
35
42
49
Day of Outbreak

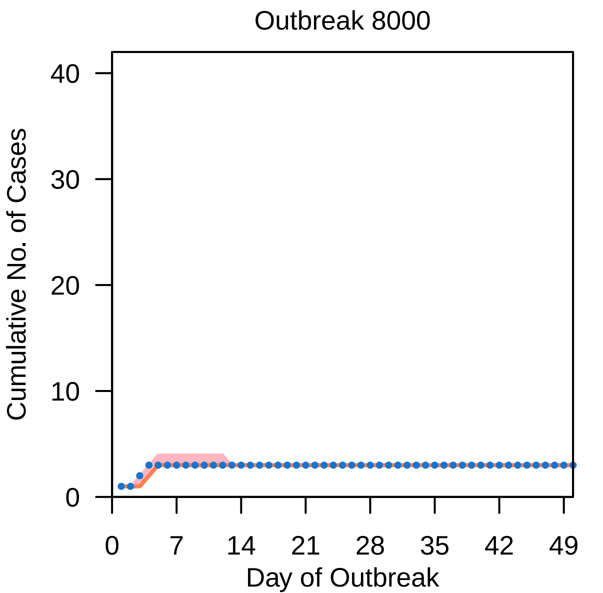
Outbreak 8000
Cumulative No. of Cases
0
10
20
30
40
0
7
14
21
28
35
42
49
Day of Outbreak

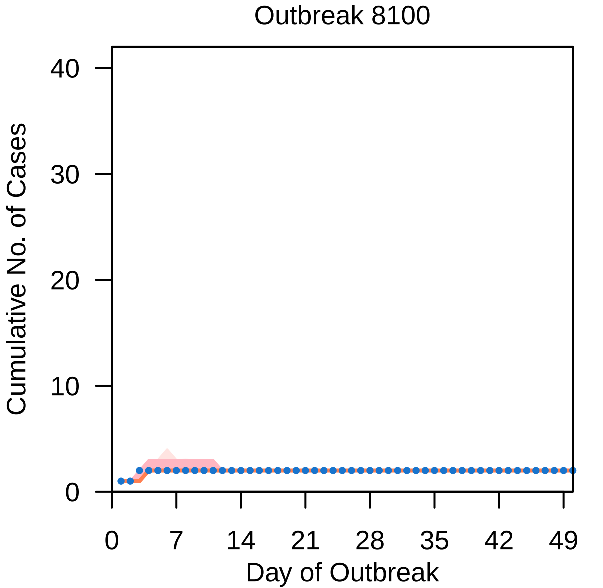
Outbreak 8100
Cumulative No. of Cases
Day of Outbreak
0
10
20
30
40
0
7
14
21
28
35
42
49

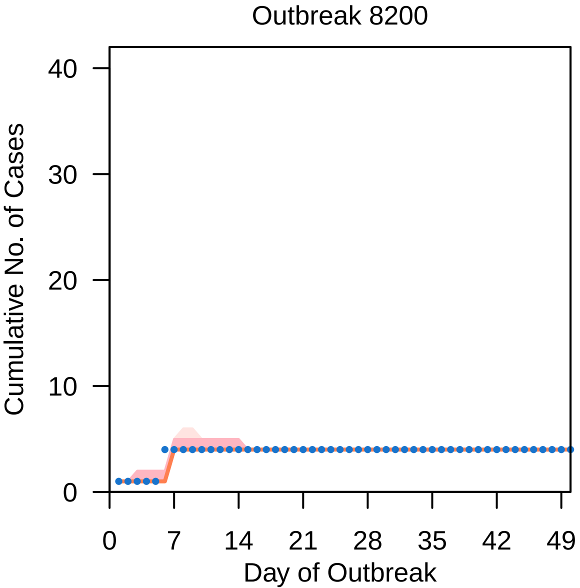

Outbreak 8200
Cumulative No. of Cases
0
10
20
30
40
0
7
14
21
28
35
42
49
Day of Outbreak

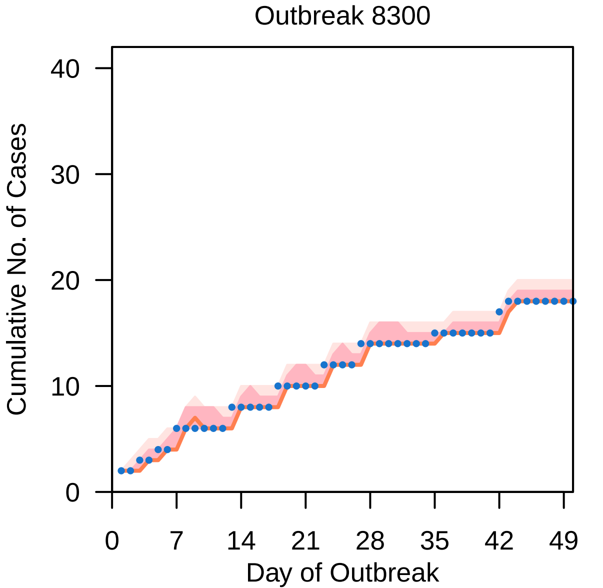
Outbreak 8300
Cumulative No. of Cases
Day of Outbreak
0
10
20
30
40
0
7
14
21
28
35
42
49

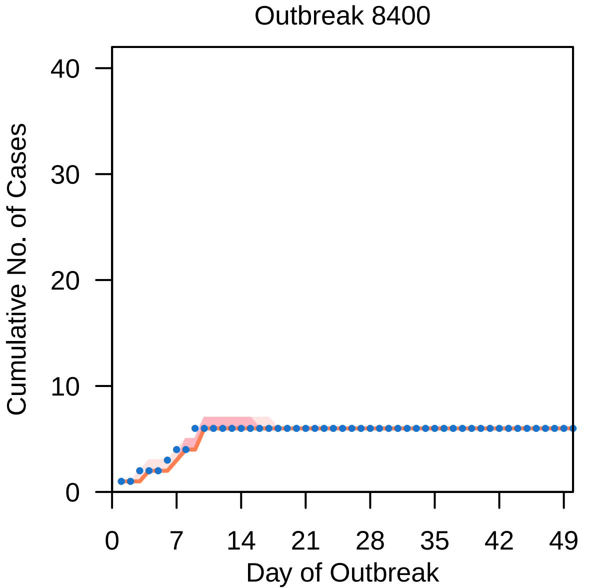

Outbreak 8400
Cumulative No. of Cases
Day of Outbreak
0
10
20
30
40
0
7
14
21
28
35
42
49

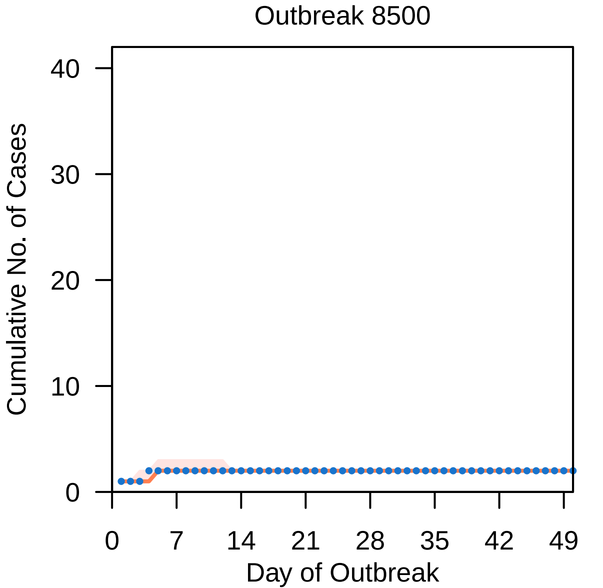
Outbreak 8500
Cumulative No. of Cases
0
10
20
30
40
0
7
14
21
28
35
42
49
Day of Outbreak

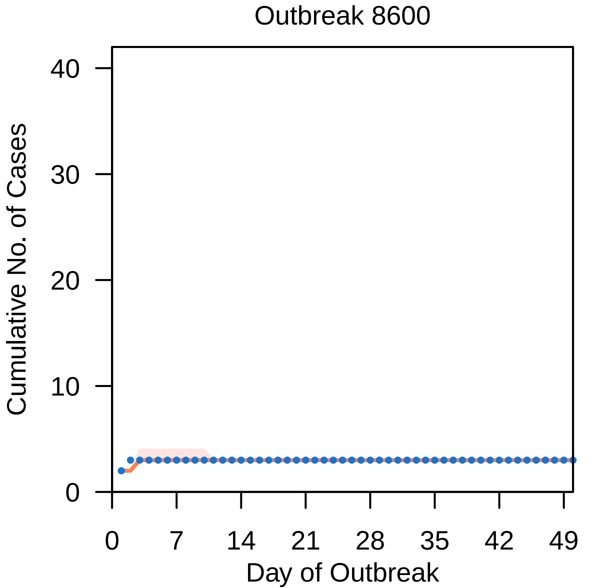

Outbreak 8600
Cumulative No. of Cases
0
10
20
30
40
0
7
14
21
28
35
42
49
Day of Outbreak

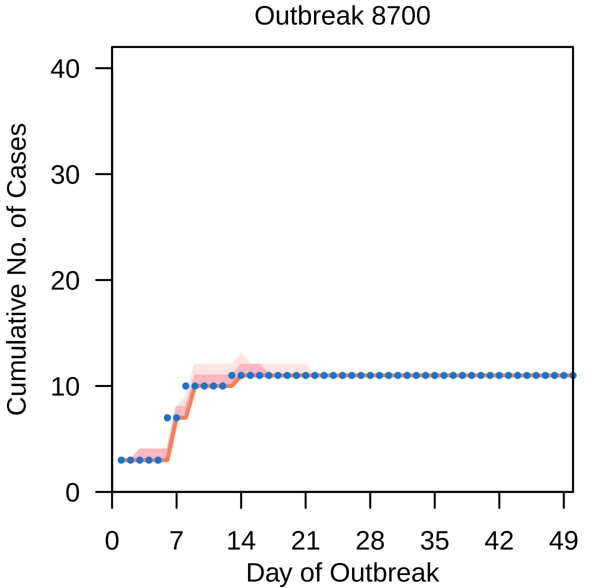

Outbreak 8700
Cumulative No. of Cases
0
10
20
30
40
0
7
14
21
28
35
42
49
Day of Outbreak

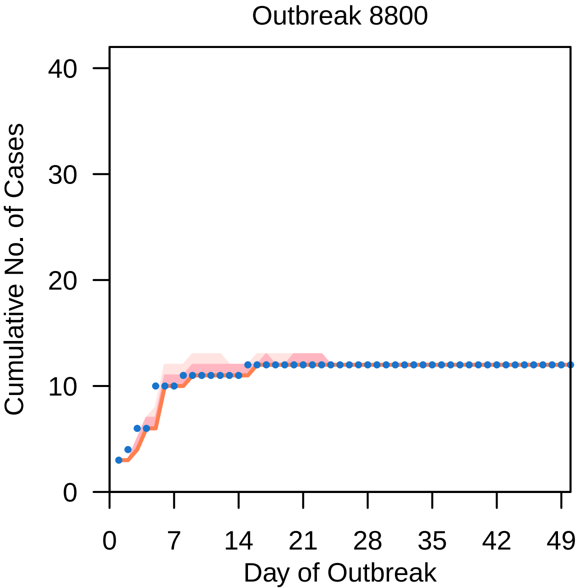

Outbreak 8800
Cumulative No. of Cases
0
10
20
30
40
0
7
14
21
28
35
42
49
Day of Outbreak

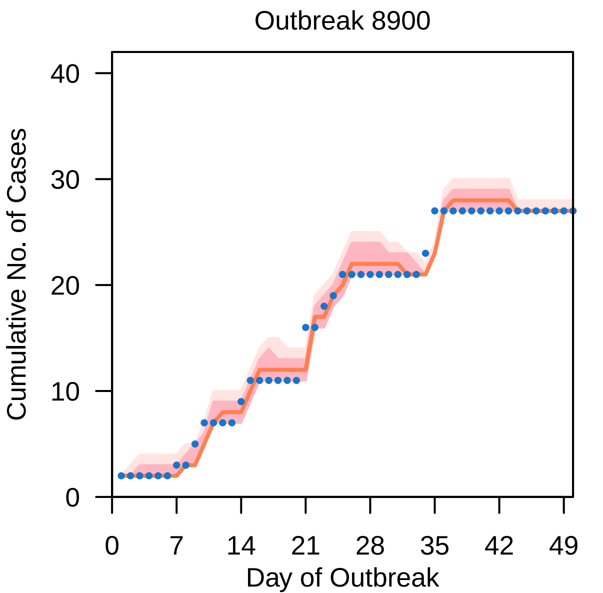

Outbreak 8900
Cumulative No. of Cases
Day of Outbreak
0
10
20
30
40
0
7
14
21
28
35
42
49

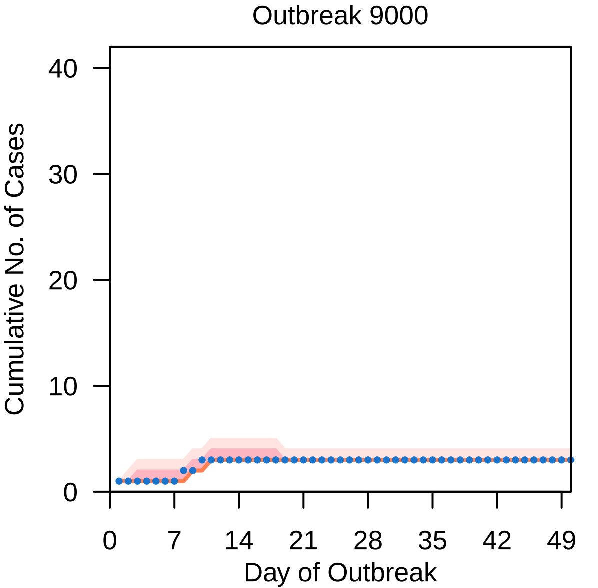

Outbreak 9000
Cumulative No. of Cases
0
10
20
30
40
0
7
14
21
28
35
42
49
Day of Outbreak

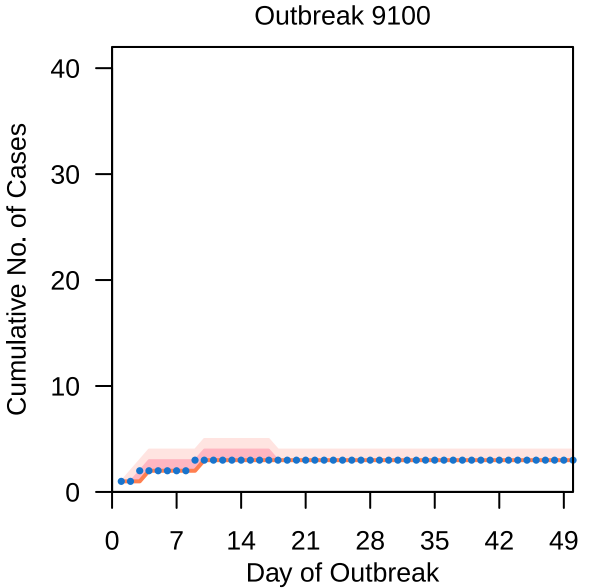

Outbreak 9100
Cumulative No. of Cases
Day of Outbreak
0
10
20
30
40
0
7
14
21
28
35
42
49

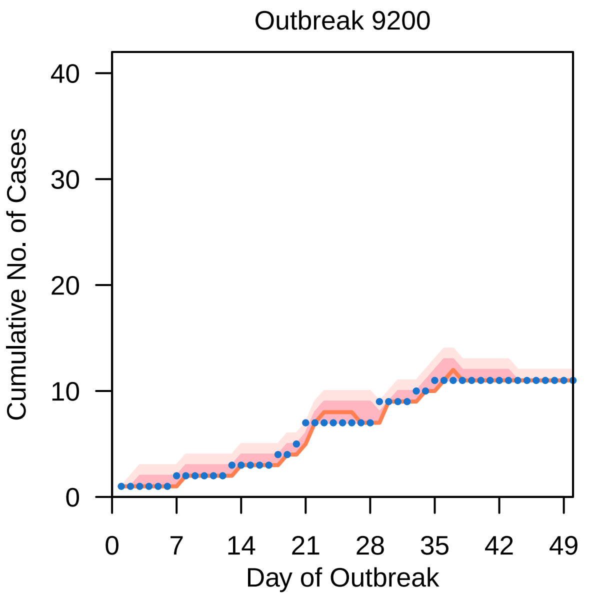

Outbreak 9200
Cumulative No. of Cases
0
10
20
30
40
0
7
14
21
28
35
42
49
Day of Outbreak

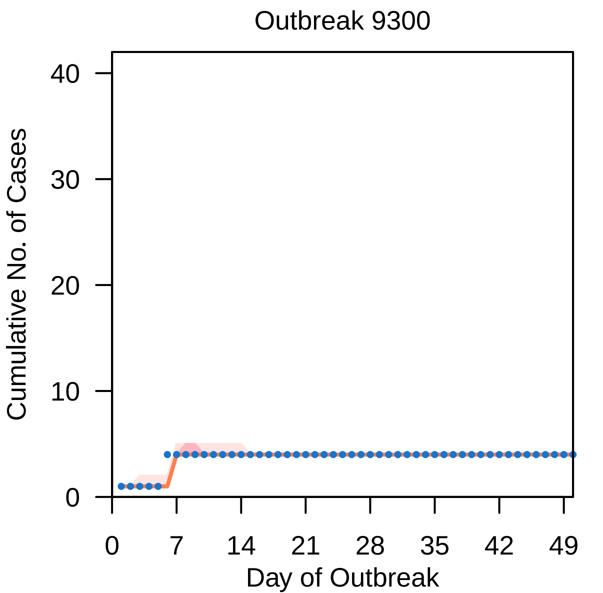
Outbreak 9300
Cumulative No. of Cases
0
10
20
30
40
0
7
14
21
28
35
42
49
Day of Outbreak

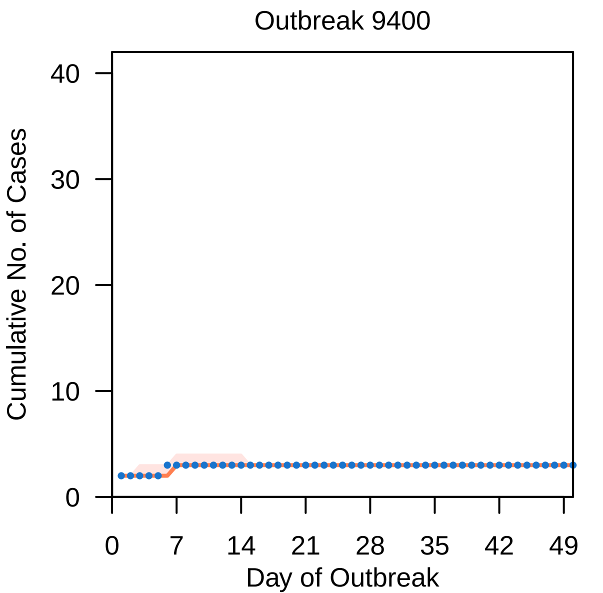
Outbreak 9400
Cumulative No. of Cases
0
10
20
30
40
Day of Outbreak
0
7
14
21
28
35
42
49

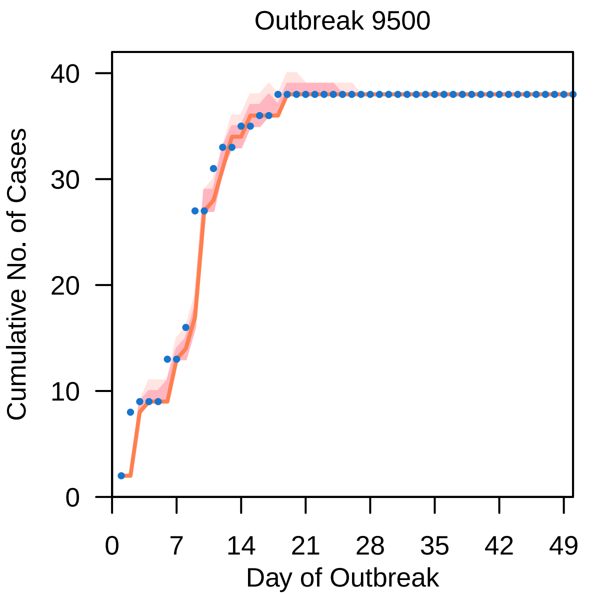

Outbreak 9500
Cumulative No. of Cases
Day of Outbreak
0
10
20
30
40
0
7
14
21
28
35
42
49

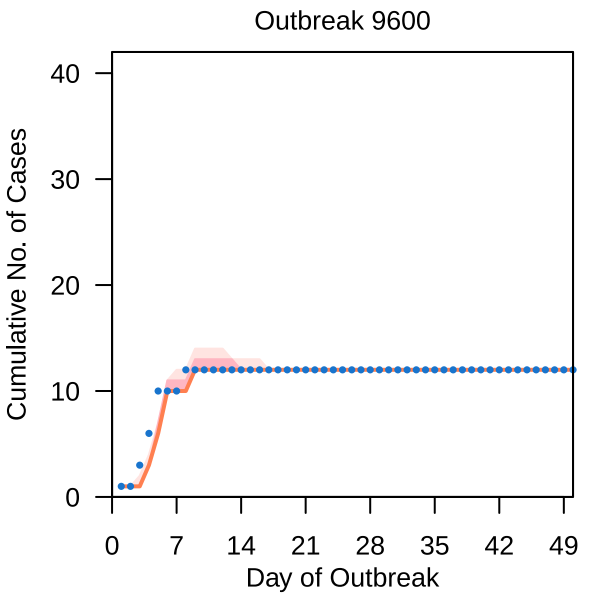
Outbreak 9600
Cumulative No. of Cases
0
10
20
30
40
0
7
14
21
28
35
42
49
Day of Outbreak

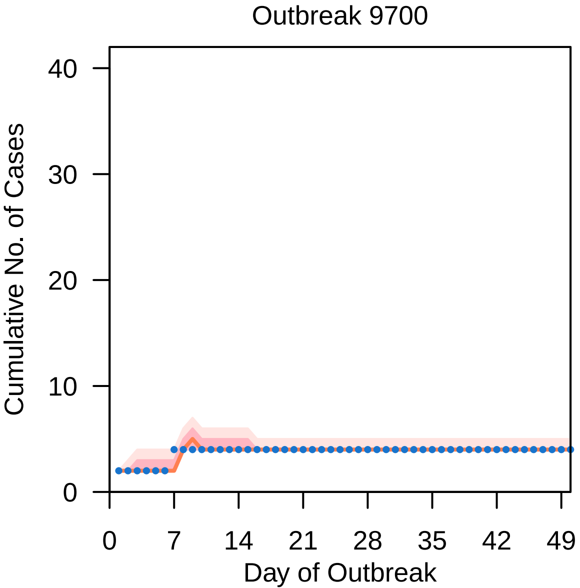

Outbreak 9700
Cumulative No. of Cases
0
10
20
30
40
0
7
14
21
28
35
42
49
Day of Outbreak

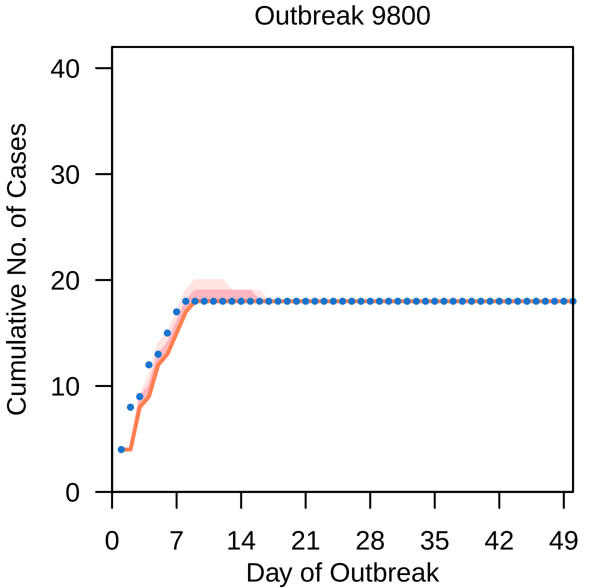
Outbreak 9800
Cumulative No. of Cases
0
10
20
30
40
0
7
14
21
28
35
42
49
Day of Outbreak

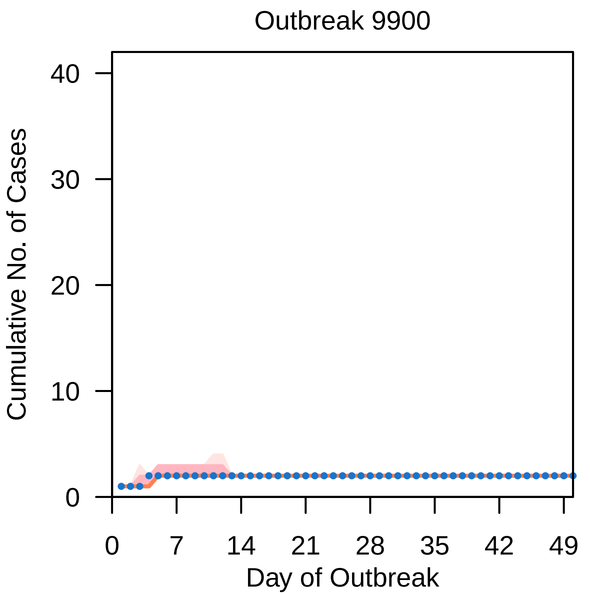

Outbreak 9900
Cumulative No. of Cases
0
10
20
30
40
0
7
14
21
28
35
42
49
Day of Outbreak
