# Supplementary material for: The Effect of School Closure on Hand, Foot, and Mouth Disease Transmission in Singapore: A Modeling Approach
Source: Am J Trop Med Hyg. 2018 Oct 22;99(6):1625–32. doi: 10.4269/ajtmh.18-0099 (PMC6283473; doi:10.4269/ajtmh.18-0099)
Supplement: Supplementary file 3 [file tpmd180099.SD3.pdf]

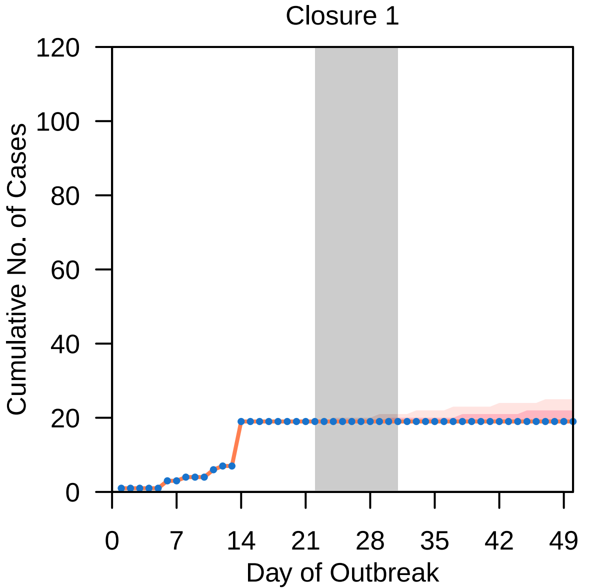

Closure 1
120
100
80
60
40
20
0
Cumulative No. of Cases
0
7
14
21
28
35
42
49
Day of Outbreak

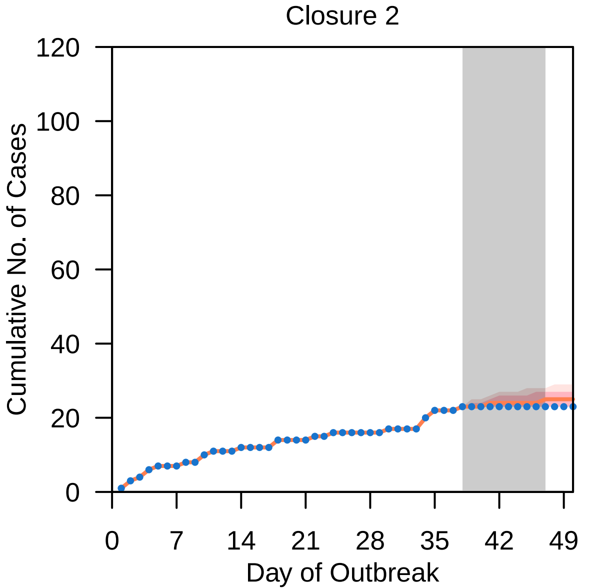

Closure 2
Cumulative No. of Cases
0
20
40
60
80
100
120
0
7
14
21
28
35
42
49
Day of Outbreak

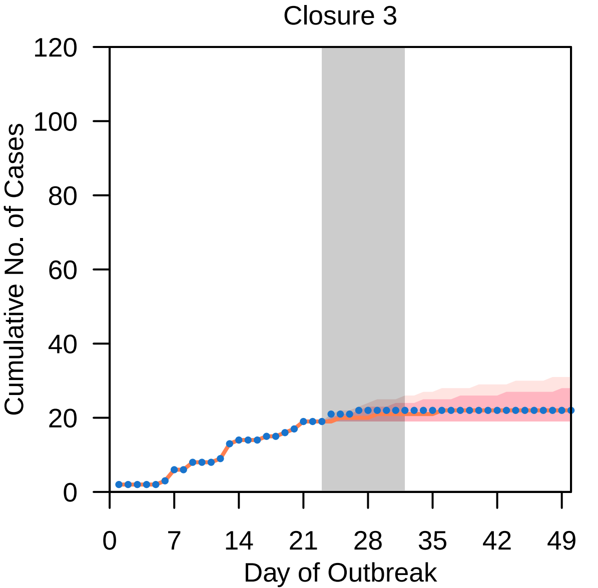

Closure 3
Cumulative No. of Cases
0
20
40
60
80
100
120
0
7
14
21
28
35
42
49
Day of Outbreak

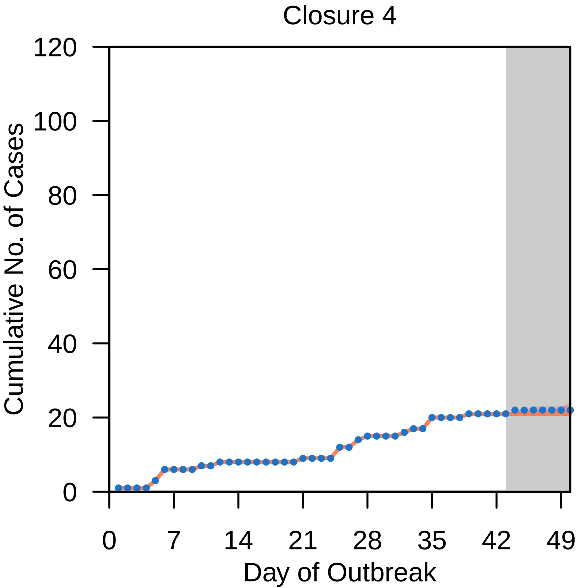

Closure 4
Cumulative No. of Cases
120
100
80
60
40
20
0
0
7
14
21
28
35
42
49
Day of Outbreak

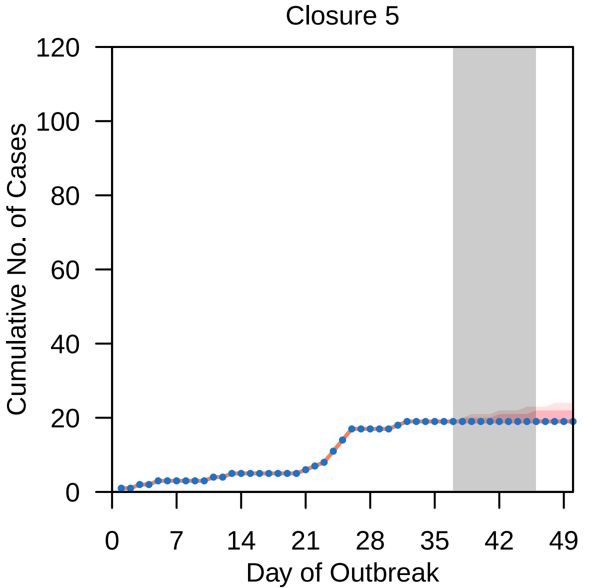
Closure 5
Cumulative No. of Cases
0
20
40
60
80
100
120
0
7
14
21
28
35
42
49
Day of Outbreak

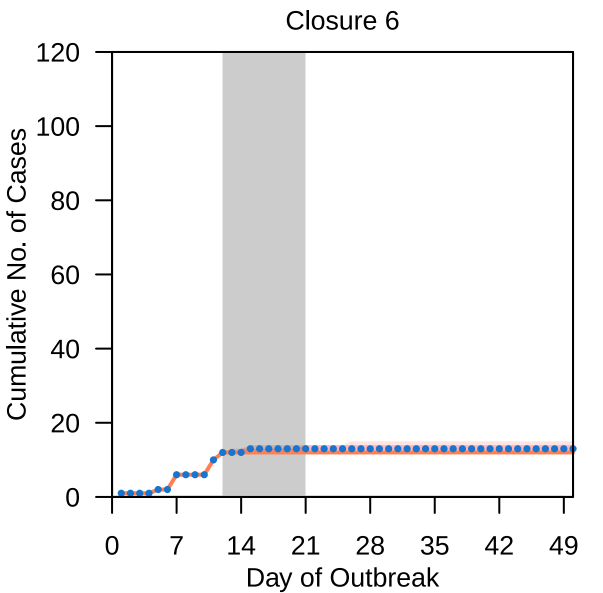
Closure 6
Cumulative No. of Cases
120
100
80
60
40
20
0
0
7
14
21
28
35
42
49
Day of Outbreak

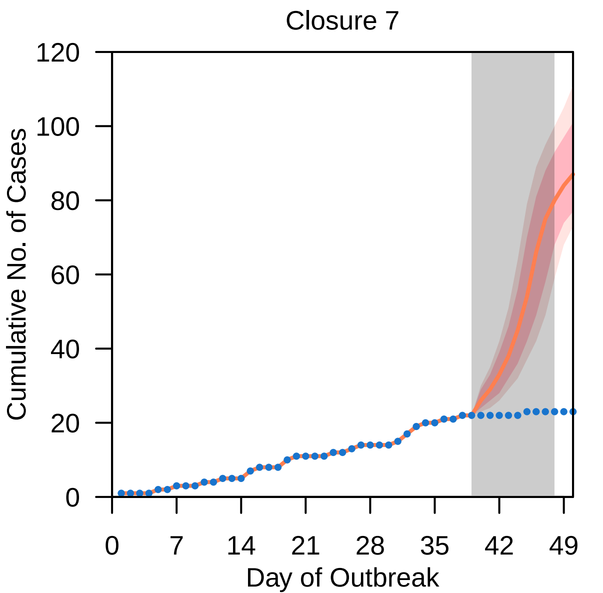
Closure 7
Cumulative No. of Cases
120
100
80
60
40
20
0
0
7
14
21
28
35
42
49
Day of Outbreak

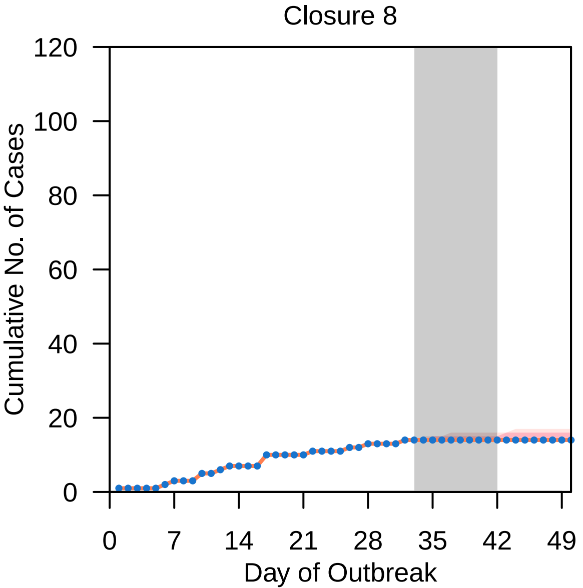

Closure 8
Cumulative No. of Cases
0
20
40
60
80
100
120
0
7
14
21
28
35
42
49
Day of Outbreak

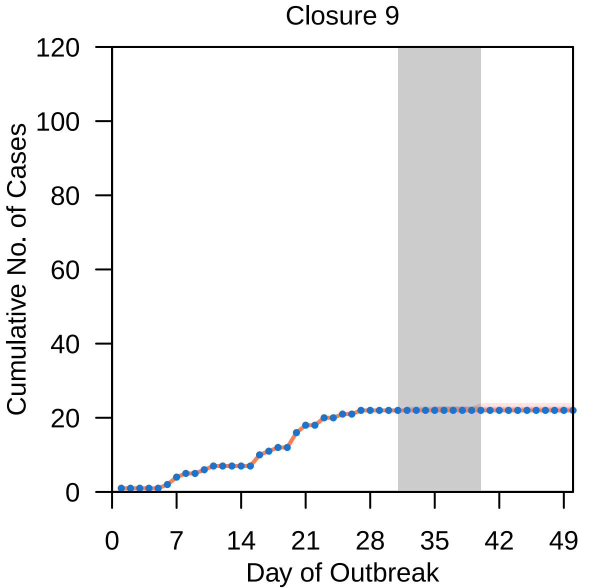
Closure 9
Cumulative No. of Cases
0
20
40
60
80
100
120
0
7
14
21
28
35
42
49
Day of Outbreak

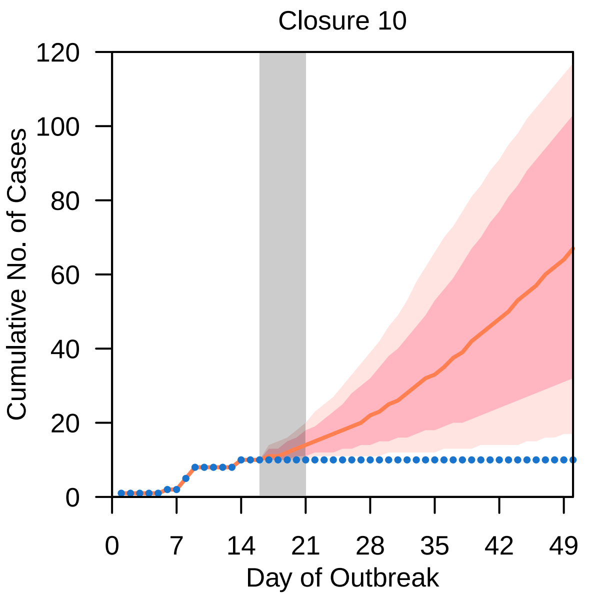

Closure 10
Cumulative No. of Cases
0
20
40
60
80
100
120
0
7
14
21
28
35
42
49
Day of Outbreak

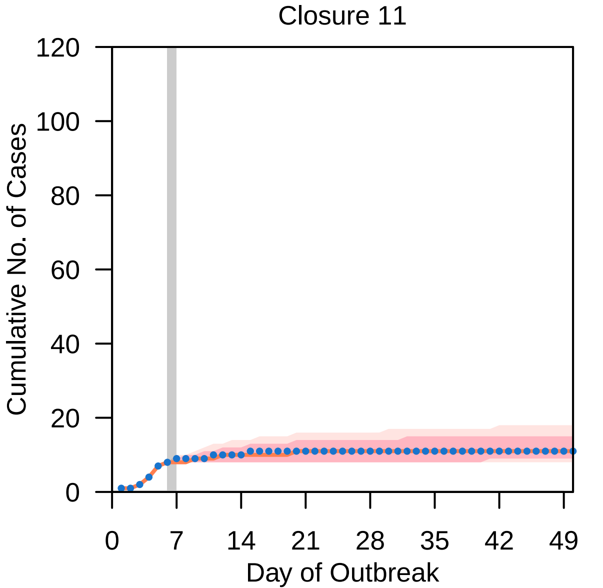

Closure 11
120
100
80
60
40
20
0
Cumulative No. of Cases
0
7
14
21
28
35
42
49
Day of Outbreak

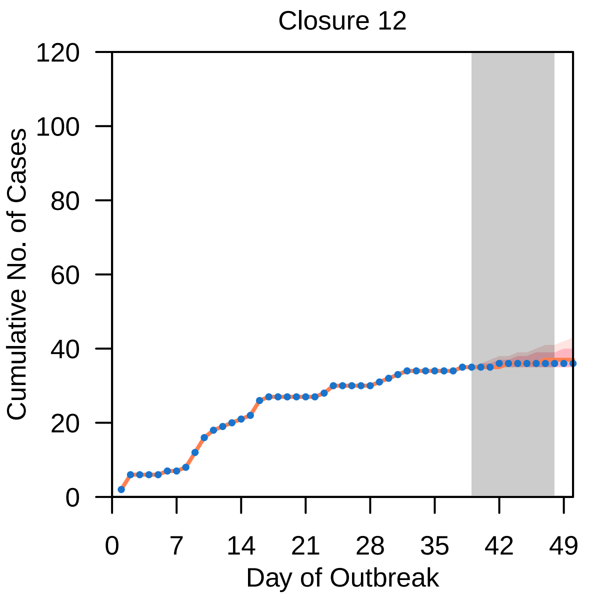
Closure 12
Cumulative No. of Cases
0
20
40
60
80
100
120
0
7
14
21
28
35
42
49
Day of Outbreak

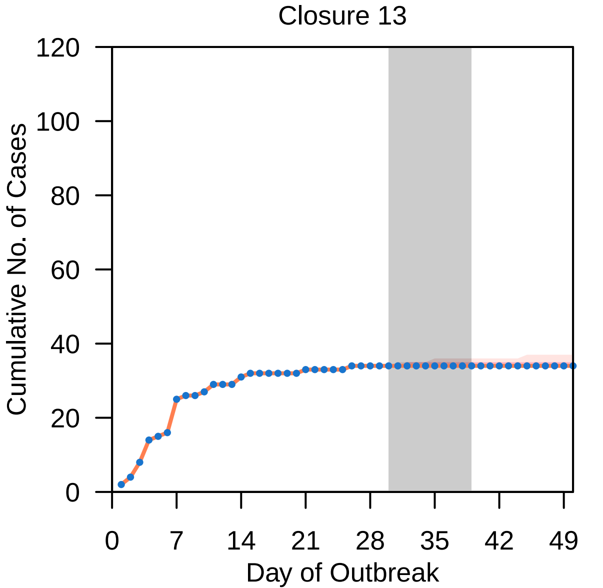

Closure 13
Cumulative No. of Cases
0
20
40
60
80
100
120
0
7
14
21
28
35
42
49
Day of Outbreak

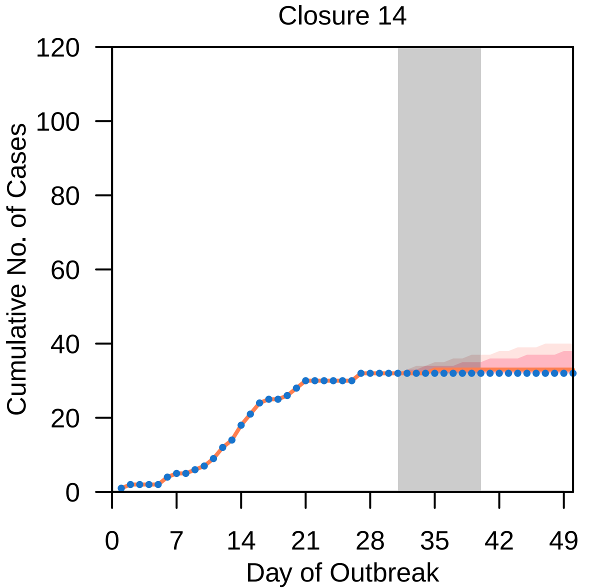

Closure 14
Cumulative No. of Cases
Day of Outbreak
0
20
40
60
80
100
120
0
7
14
21
28
35
42
49

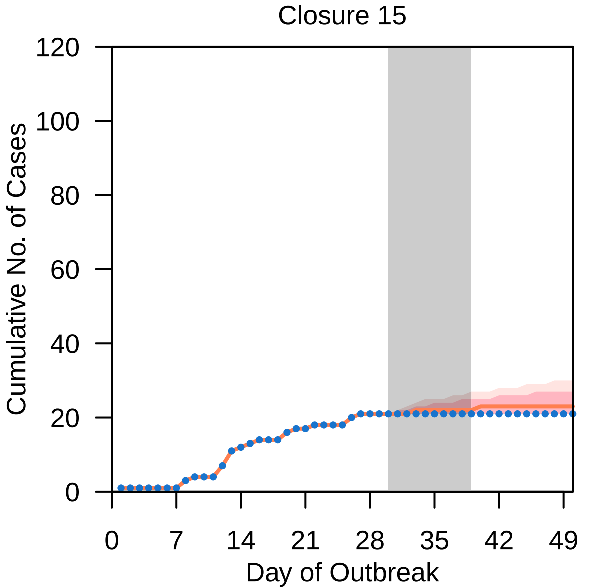

Closure 15
Cumulative No. of Cases
0
20
40
60
80
100
120
0
7
14
21
28
35
42
49
Day of Outbreak

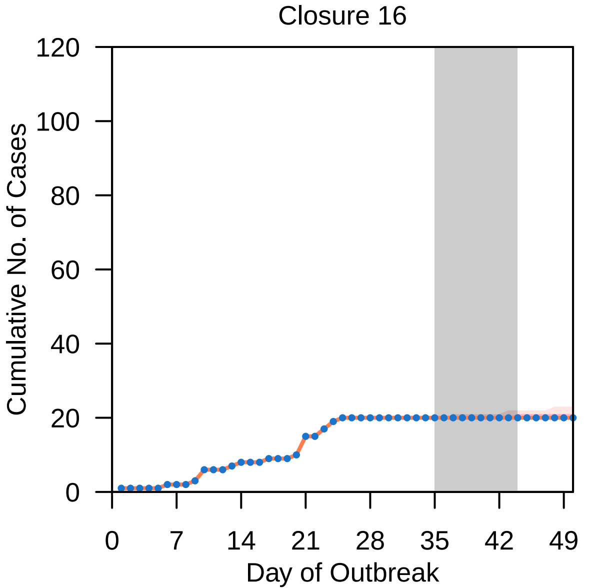

Closure 16
Cumulative No. of Cases
0
20
40
60
80
100
120
0
7
14
21
28
35
42
49
Day of Outbreak

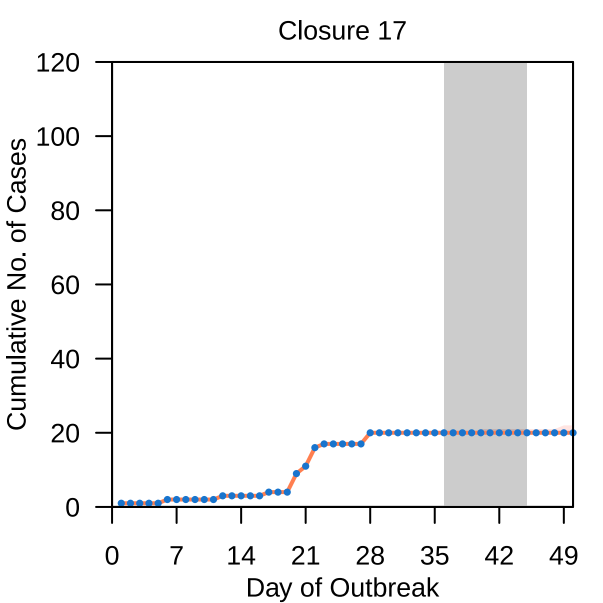
Closure 17
Cumulative No. of Cases
0
20
40
60
80
100
120
0
7
14
21
28
35
42
49
Day of Outbreak

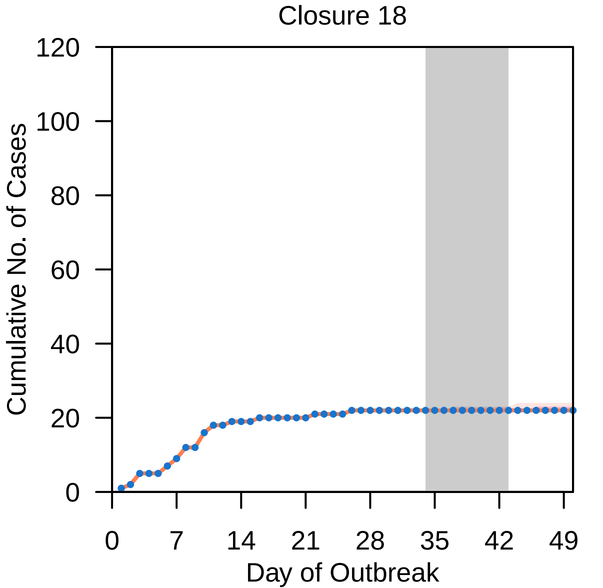

Closure 18
Cumulative No. of Cases
Day of Outbreak
0
20
40
60
80
100
120
0
7
14
21
28
35
42
49

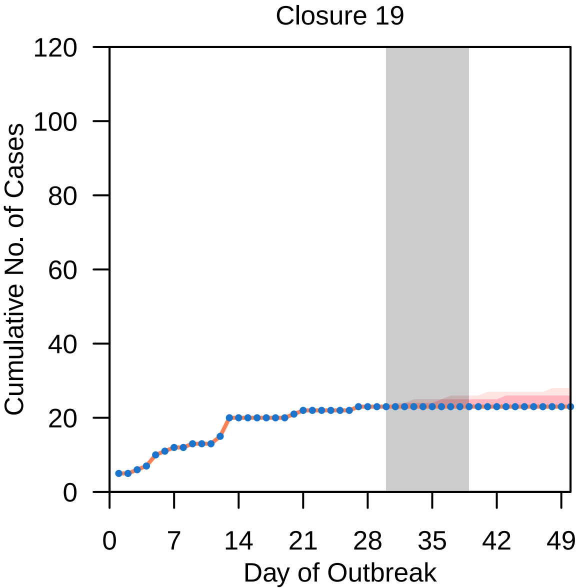

Closure 19
Cumulative No. of Cases
0
20
40
60
80
100
120
0
7
14
21
28
35
42
49
Day of Outbreak

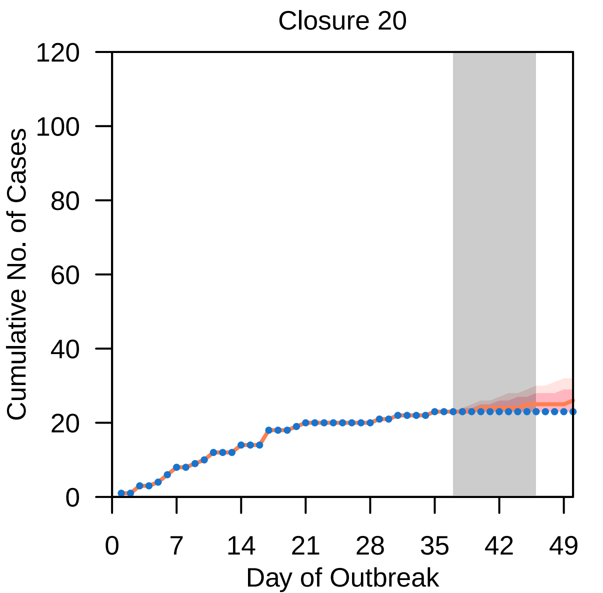

Closure 20
Cumulative No. of Cases
0
20
40
60
80
100
120
0
7
14
21
28
35
42
49
Day of Outbreak

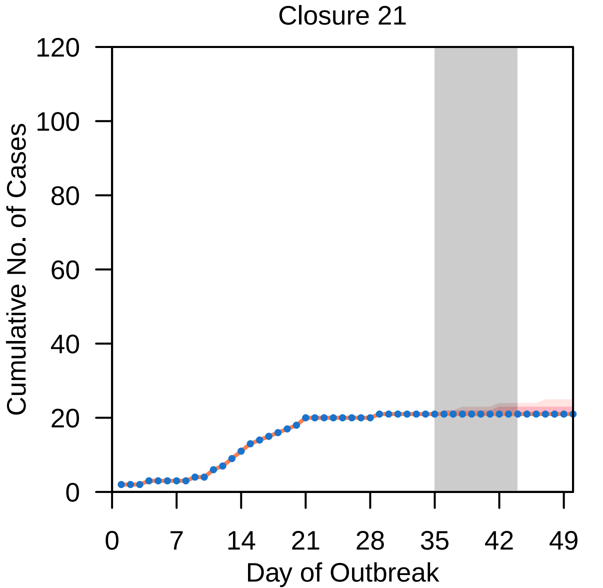

Closure 21
Cumulative No. of Cases
Day of Outbreak
0
20
40
60
80
100
120
0
7
14
21
28
35
42
49

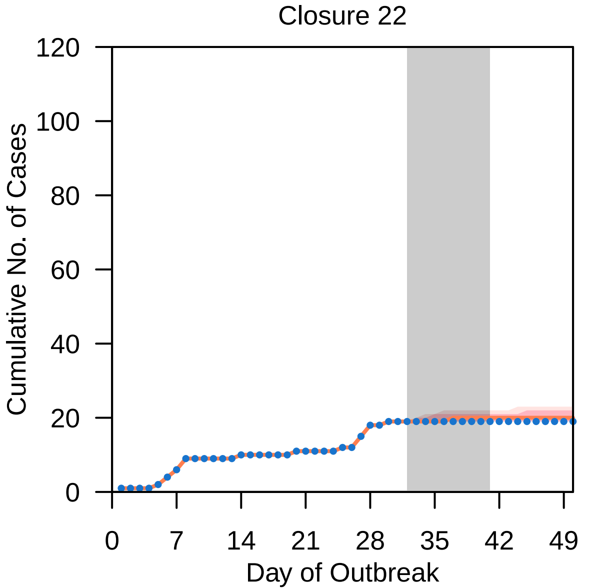

Closure 22
Cumulative No. of Cases
Day of Outbreak
0
20
40
60
80
100
120
0
7
14
21
28
35
42
49

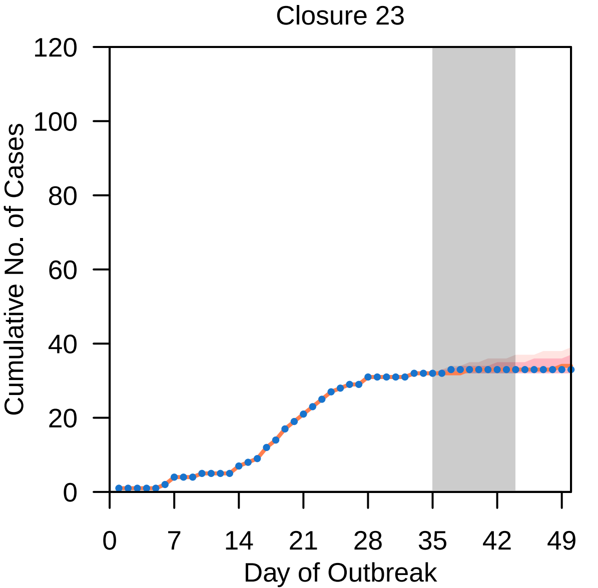
Closure 23
Cumulative No. of Cases
0
20
40
60
80
100
120
0
7
14
21
28
35
42
49
Day of Outbreak

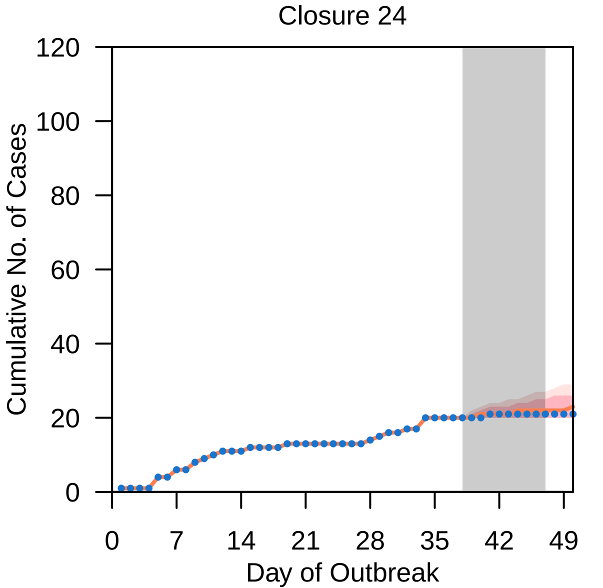

Closure 24
Cumulative No. of Cases
0
20
40
60
80
100
120
0
7
14
21
28
35
42
49
Day of Outbreak

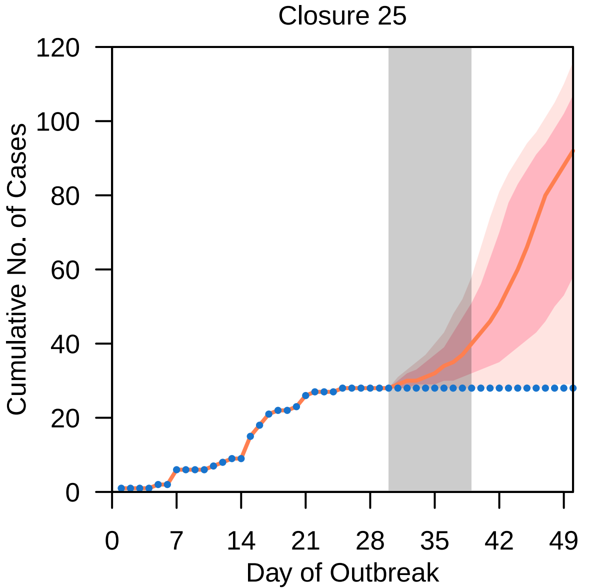

Closure 25
Cumulative No. of Cases
0
20
40
60
80
100
120
0
7
14
21
28
35
42
49
Day of Outbreak

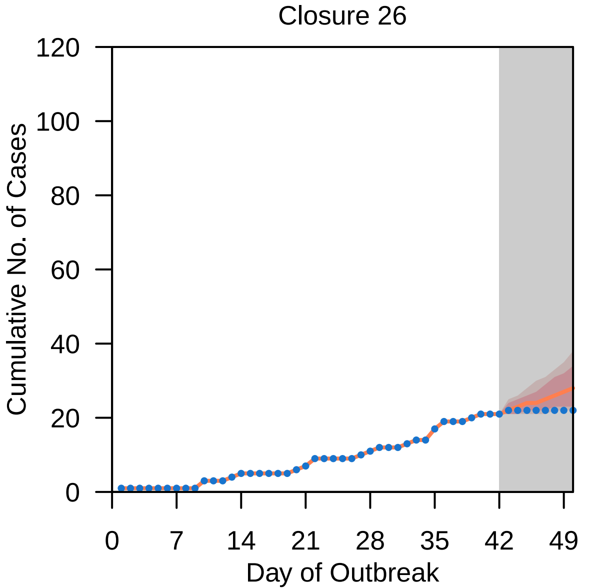

Closure 26
Cumulative No. of Cases
Day of Outbreak
0
20
40
60
80
100
120
0
7
14
21
28
35
42
49

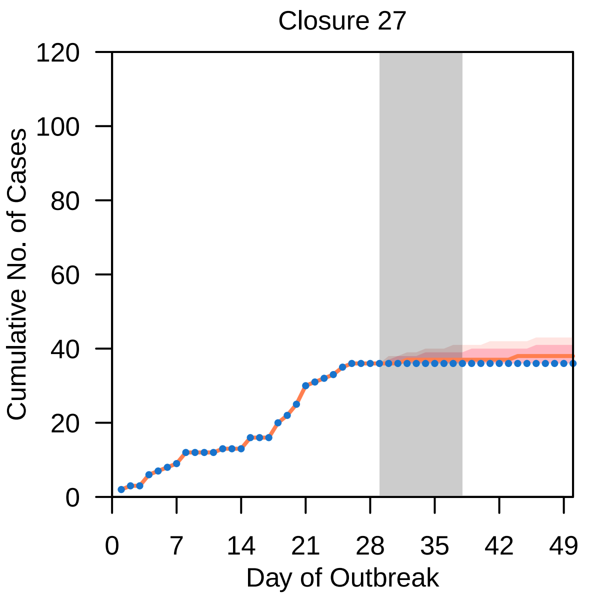

Closure 27
Cumulative No. of Cases
0
20
40
60
80
100
120
0
7
14
21
28
35
42
49
Day of Outbreak

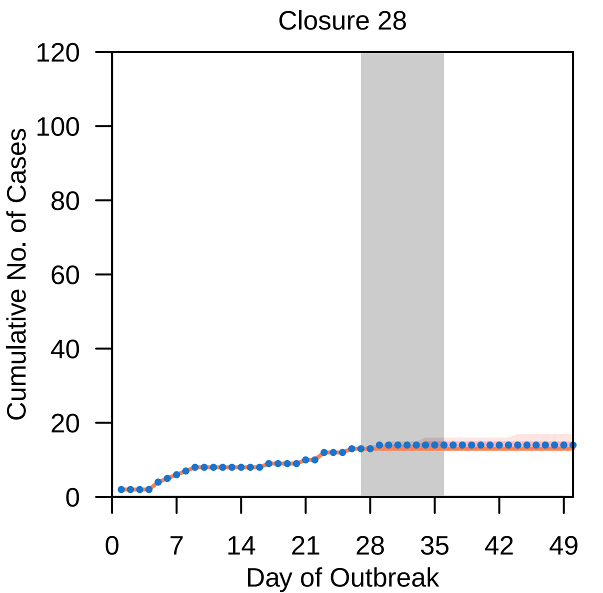
Closure 28
Cumulative No. of Cases
120
100
80
60
40
20
0
0
7
14
21
28
35
42
49
Day of Outbreak

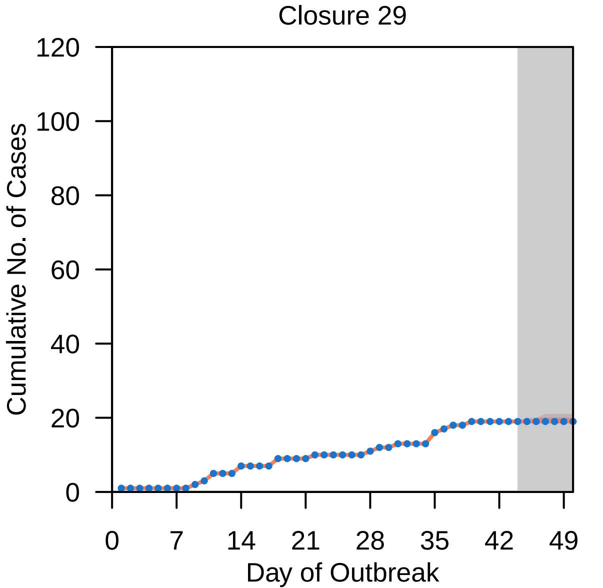

Closure 29
Cumulative No. of Cases
120
100
80
60
40
20
0
0
7
14
21
28
35
42
49
Day of Outbreak

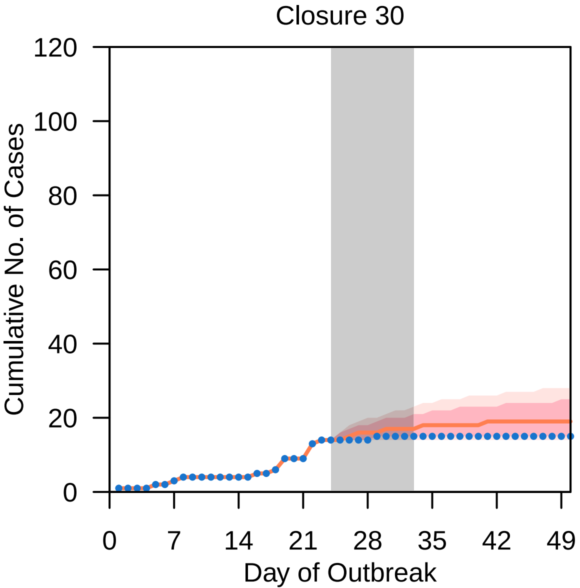

Closure 30
Cumulative No. of Cases
Day of Outbreak
0
20
40
60
80
100
120
0
7
14
21
28
35
42
49

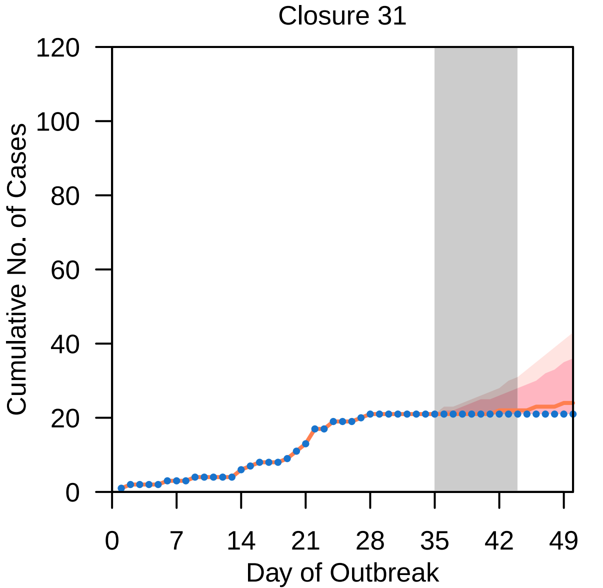
Closure 31
Cumulative No. of Cases
0
20
40
60
80
100
120
0
7
14
21
28
35
42
49
Day of Outbreak

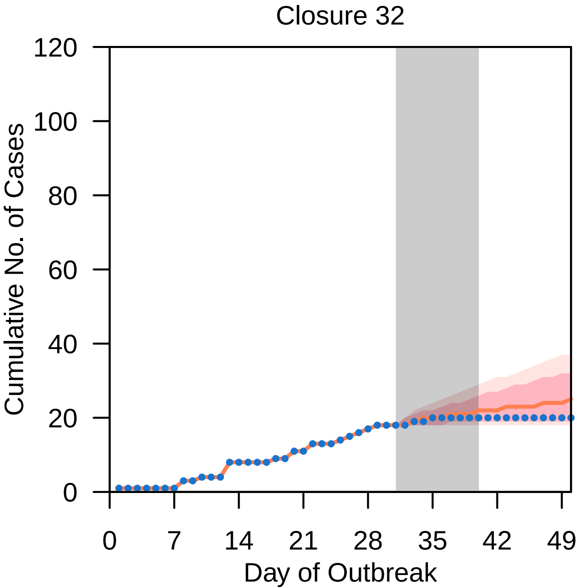

Closure 32
Cumulative No. of Cases
0
20
40
60
80
100
120
0
7
14
21
28
35
42
49
Day of Outbreak

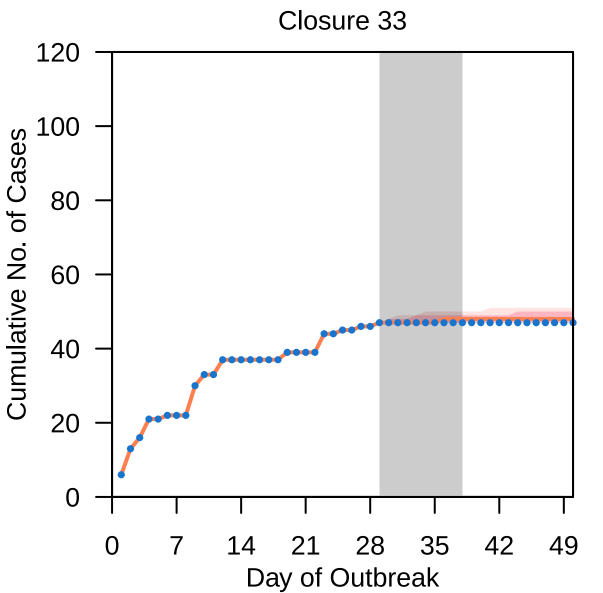
Closure 33
Cumulative No. of Cases
0
20
40
60
80
100
120
0
7
14
21
28
35
42
49
Day of Outbreak

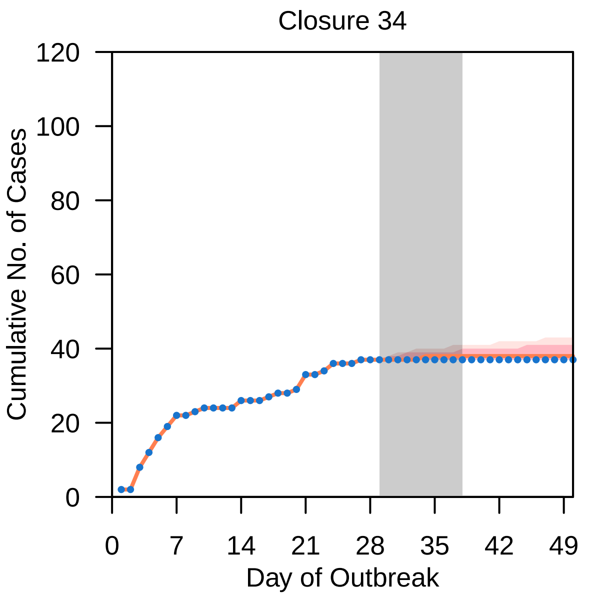

Closure 34
Cumulative No. of Cases
Day of Outbreak
0
20
40
60
80
100
120
0
7
14
21
28
35
42
49

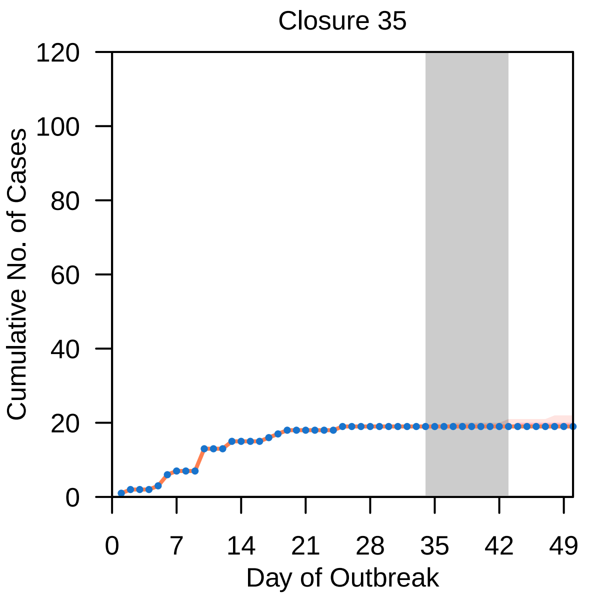

Closure 35
Cumulative No. of Cases
0
20
40
60
80
100
120
0
7
14
21
28
35
42
49
Day of Outbreak

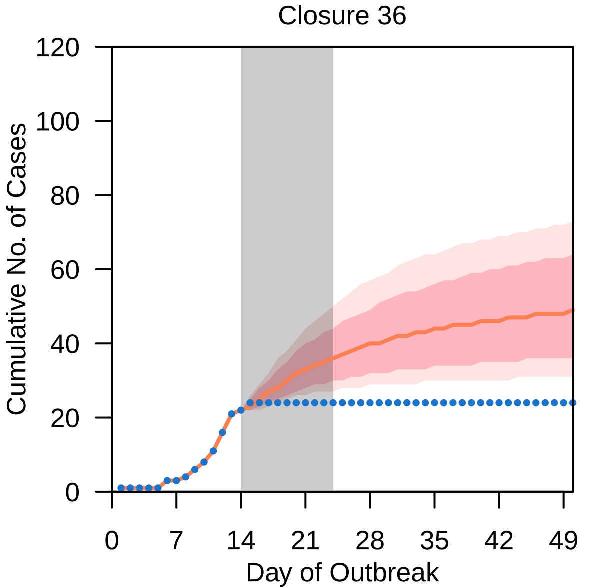

Closure 36
Cumulative No. of Cases
0
20
40
60
80
100
120
0
7
14
21
28
35
42
49
Day of Outbreak

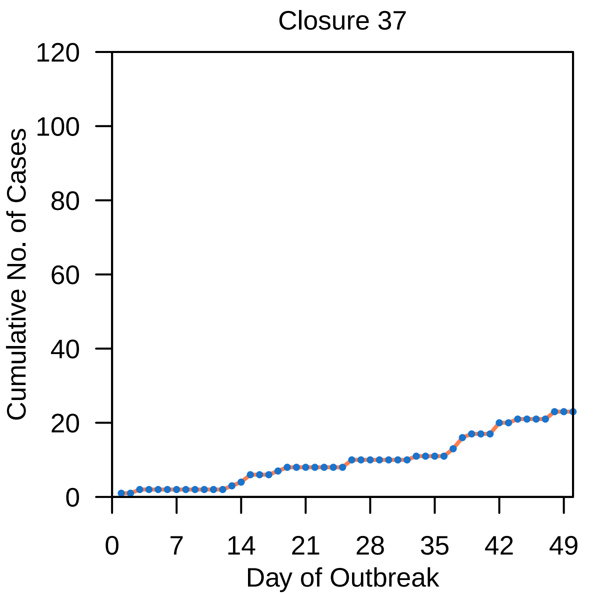

Closure 37
Cumulative No. of Cases
120
100
80
60
40
20
0
0
7
14
21
28
35
42
49
Day of Outbreak

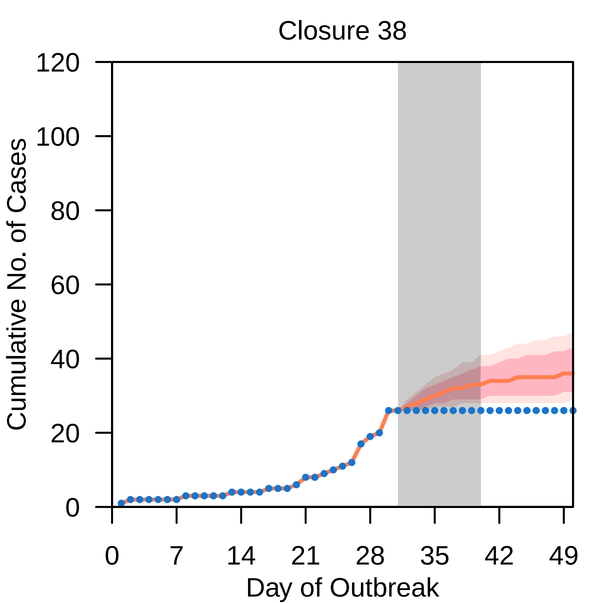
Closure 38
Cumulative No. of Cases
Day of Outbreak
0
20
40
60
80
100
120
0
7
14
21
28
35
42
49

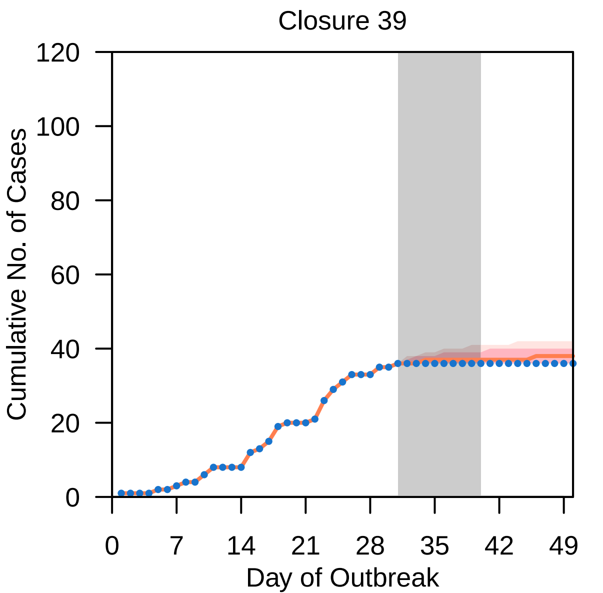

Closure 39
Cumulative No. of Cases
0
20
40
60
80
100
120
0
7
14
21
28
35
42
49
Day of Outbreak

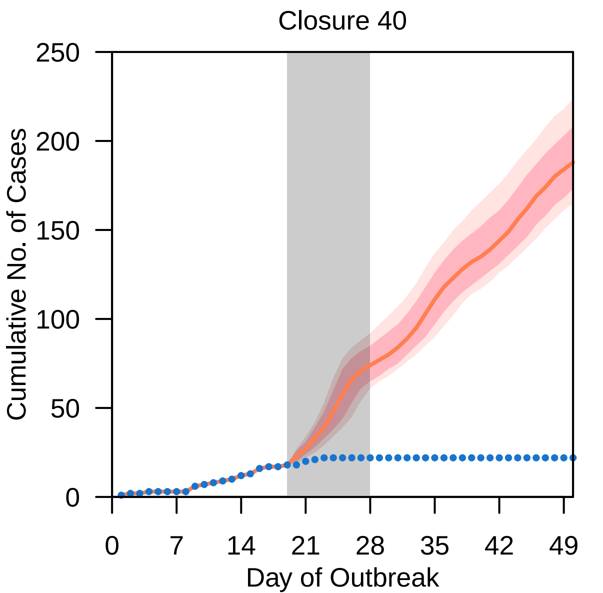

Closure 40
Cumulative No. of Cases
0
50
100
150
200
250
0
7
14
21
28
35
42
49
Day of Outbreak

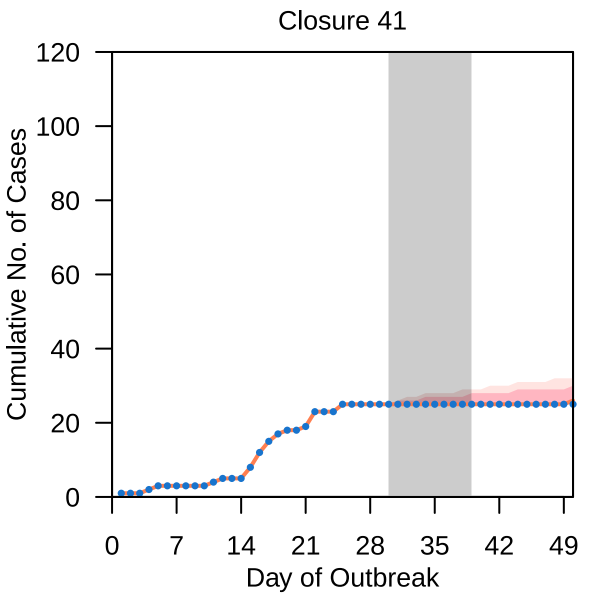

Closure 41
Cumulative No. of Cases
0
20
40
60
80
100
120
Day of Outbreak
0
7
14
21
28
35
42
49

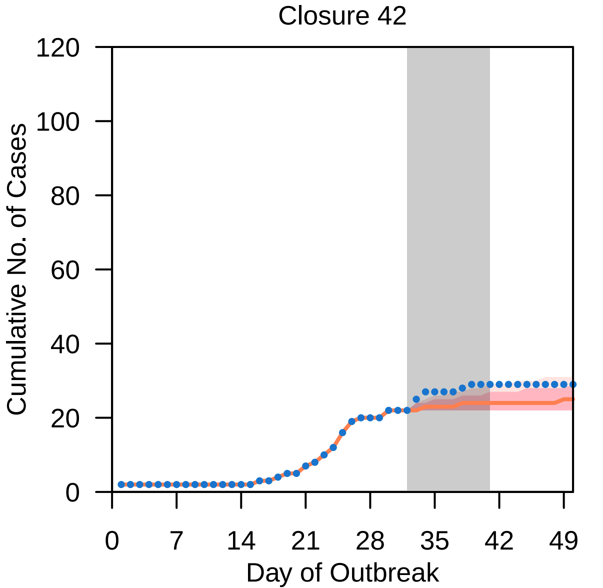

Closure 42
Cumulative No. of Cases
120
100
80
60
40
20
0
0
7
14
21
28
35
42
49
Day of Outbreak

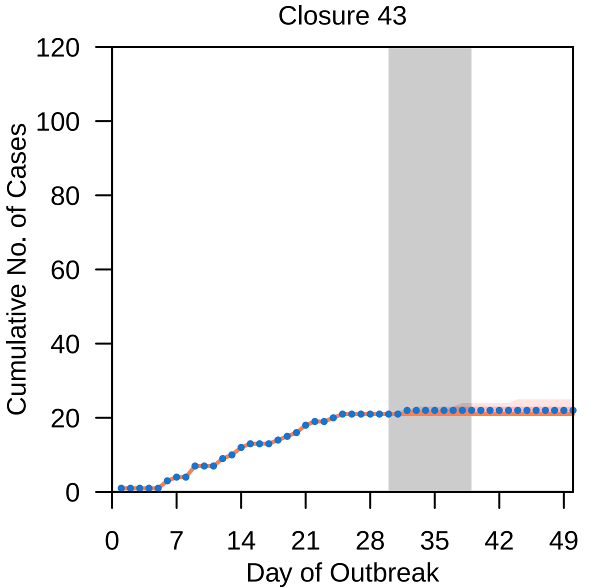

Closure 43
Cumulative No. of Cases
Day of Outbreak
0
20
40
60
80
100
120
0
7
14
21
28
35
42
49

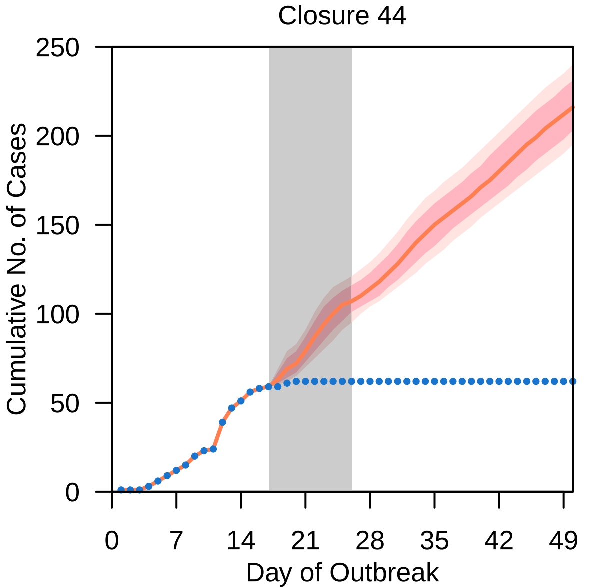

Closure 44
Cumulative No. of Cases
250
200
150
100
50
0
0
7
14
21
28
35
42
49
Day of Outbreak

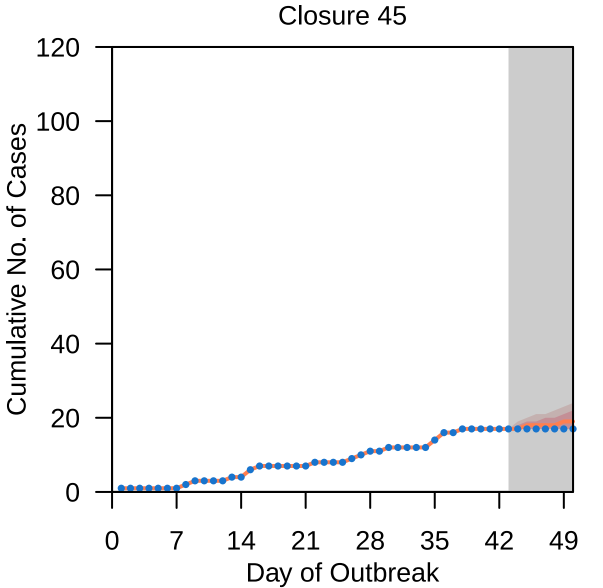

Closure 45
Cumulative No. of Cases
0
20
40
60
80
100
120
0
7
14
21
28
35
42
49
Day of Outbreak

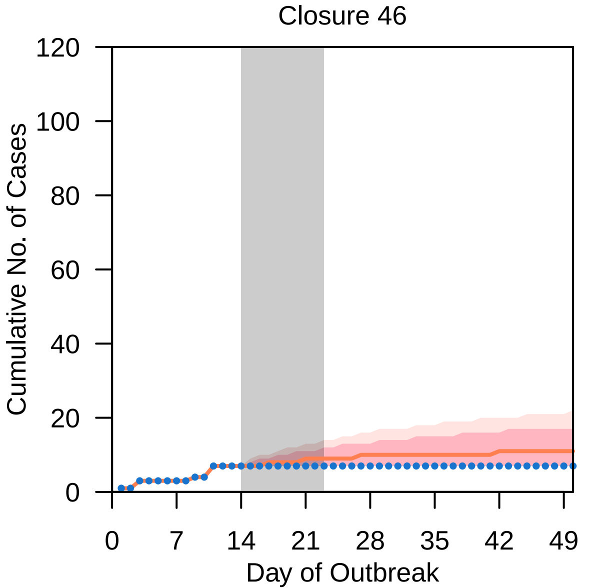

Closure 46
Cumulative No. of Cases
Day of Outbreak
0
20
40
60
80
100
120
0
7
14
21
28
35
42
49

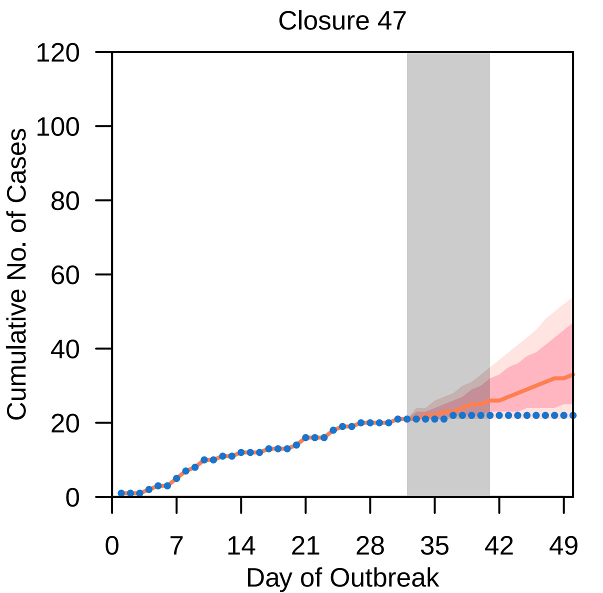

Closure 47
Cumulative No. of Cases
0
20
40
60
80
100
120
0
7
14
21
28
35
42
49
Day of Outbreak

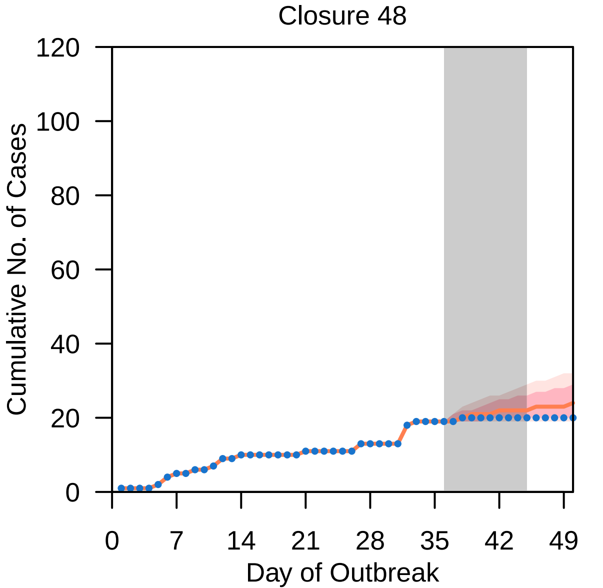

Closure 48
Cumulative No. of Cases
120
100
80
60
40
20
0
0
7
14
21
28
35
42
49
Day of Outbreak

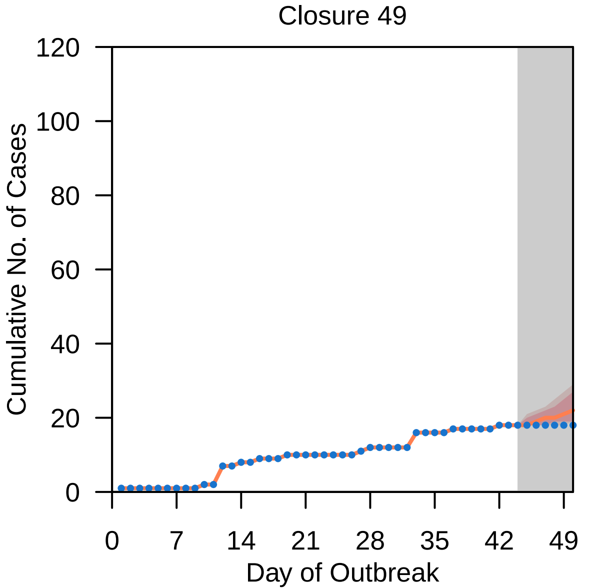

Closure 49
Cumulative No. of Cases
0
20
40
60
80
100
120
0
7
14
21
28
35
42
49
Day of Outbreak

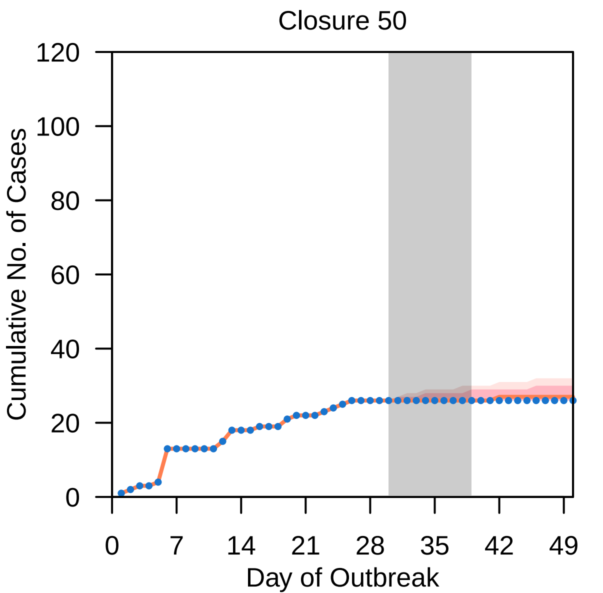

Closure 50
Cumulative No. of Cases
0
20
40
60
80
100
120
0
7
14
21
28
35
42
49
Day of Outbreak

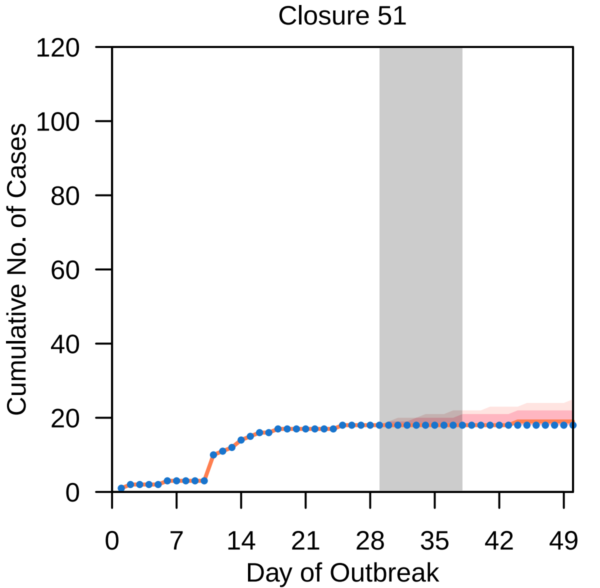
Closure 51
Cumulative No. of Cases
0
20
40
60
80
100
120
0
7
14
21
28
35
42
49
Day of Outbreak

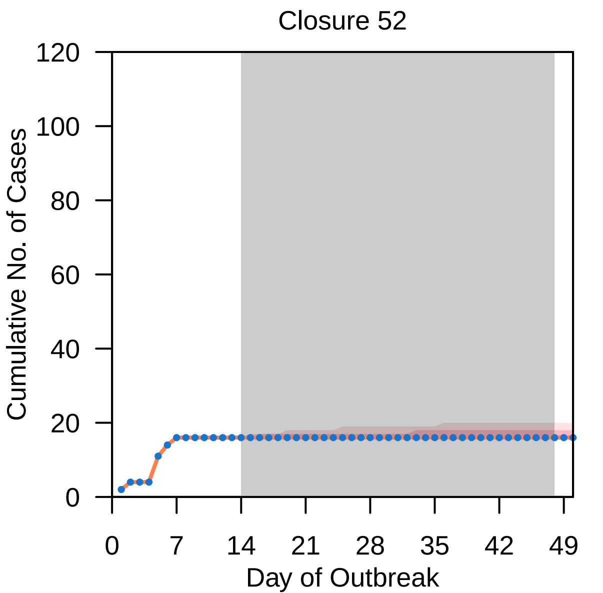
Closure 52
Cumulative No. of Cases
0
20
40
60
80
100
120
0
7
14
21
28
35
42
49
Day of Outbreak

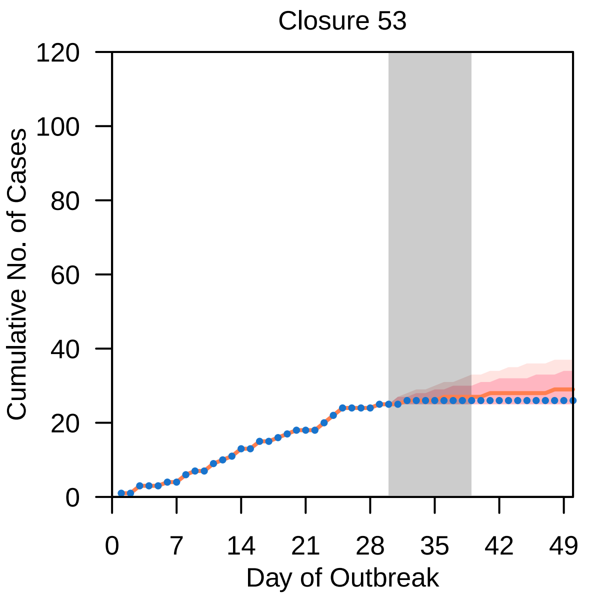

Closure 53
Cumulative No. of Cases
0
20
40
60
80
100
120
0
7
14
21
28
35
42
49
Day of Outbreak

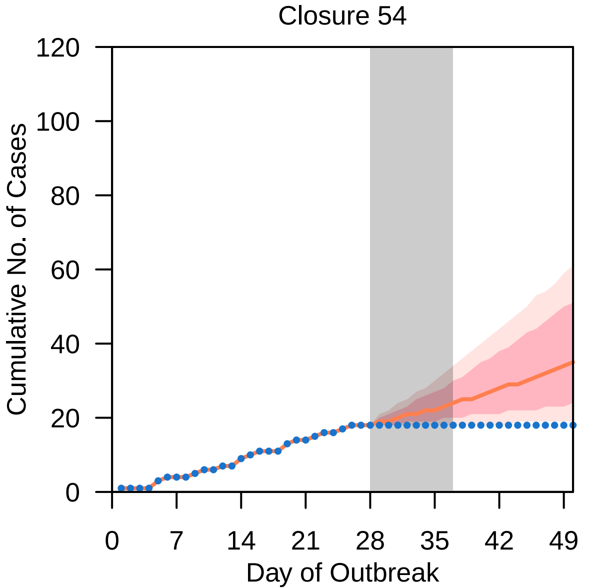

Closure 54
Cumulative No. of Cases
Day of Outbreak
0
20
40
60
80
100
120
0
7
14
21
28
35
42
49

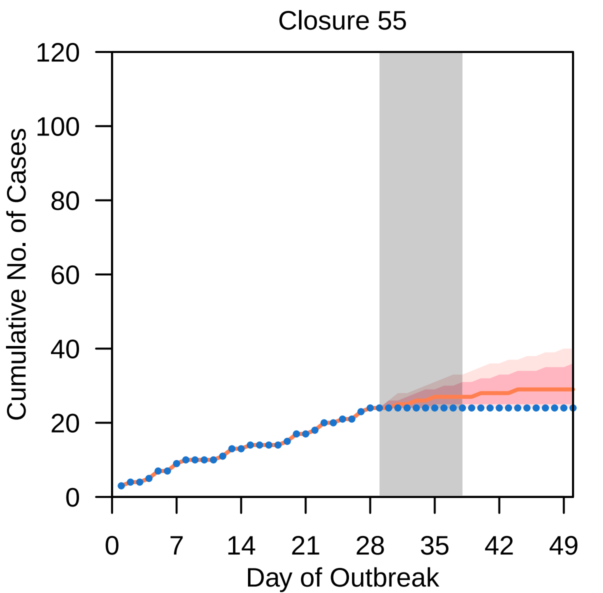

Closure 55
Cumulative No. of Cases
120
100
80
60
40
20
0
0
7
14
21
28
35
42
49
Day of Outbreak

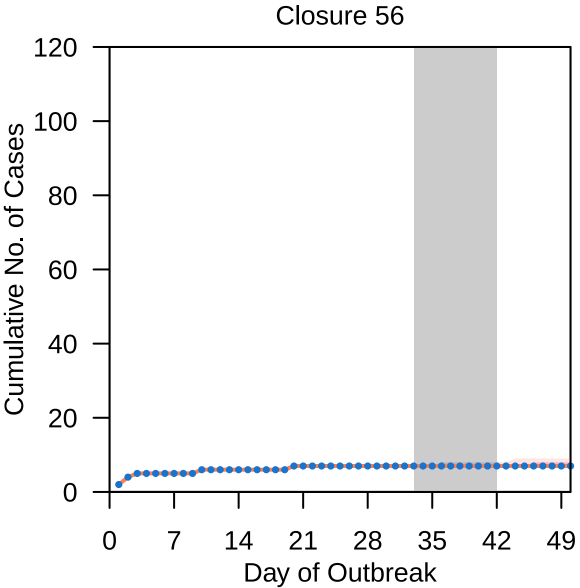

Closure 56
Cumulative No. of Cases
0
20
40
60
80
100
120
0
7
14
21
28
35
42
49
Day of Outbreak

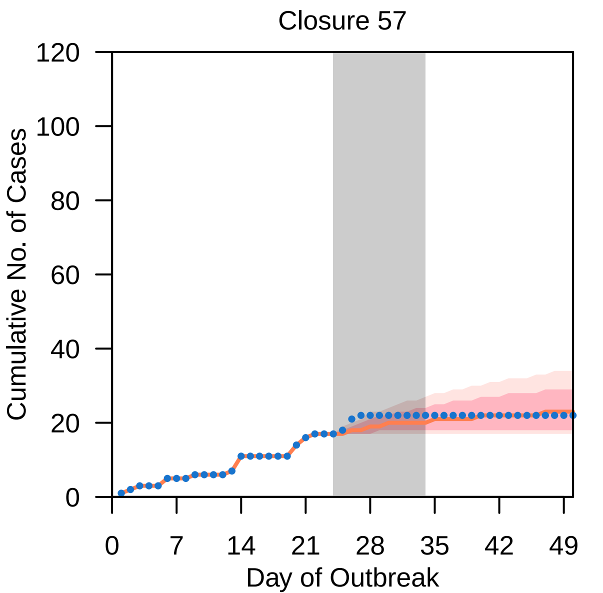

Closure 57
Cumulative No. of Cases
0
20
40
60
80
100
120
0
7
14
21
28
35
42
49
Day of Outbreak

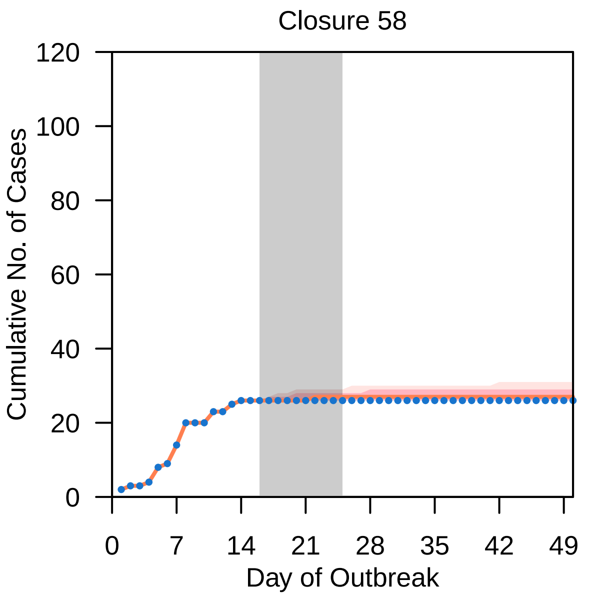
Closure 58
Cumulative No. of Cases
120
100
80
60
40
20
0
0
7
14
21
28
35
42
49
Day of Outbreak

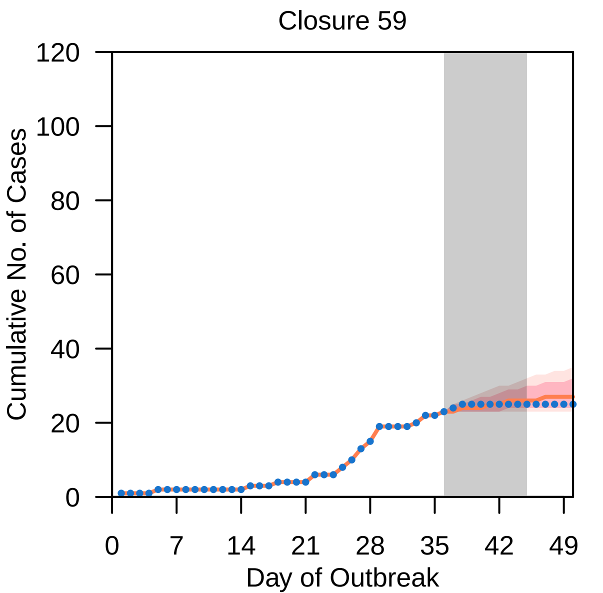

Closure 59
Cumulative No. of Cases
0
20
40
60
80
100
120
0
7
14
21
28
35
42
49
Day of Outbreak

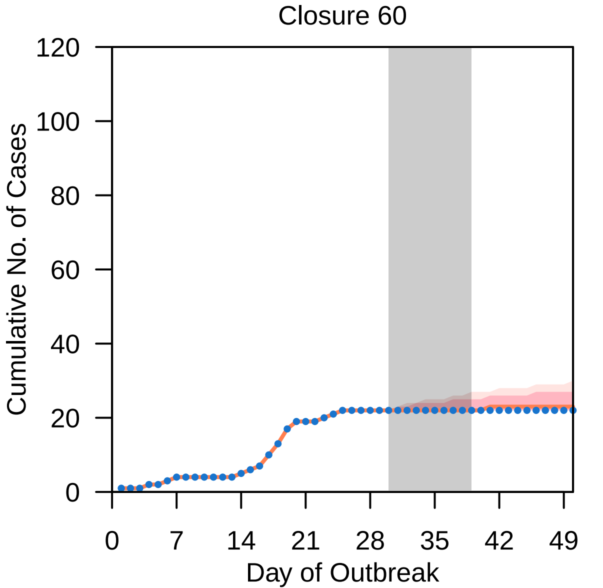
Closure 60
Cumulative No. of Cases
0
20
40
60
80
100
120
0
7
14
21
28
35
42
49
Day of Outbreak

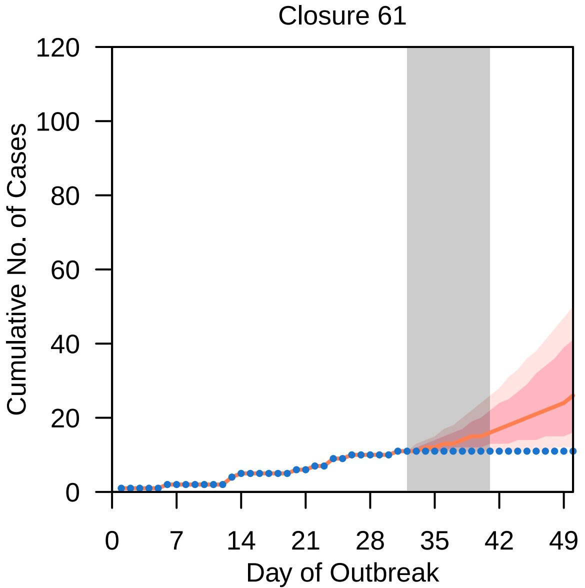

Closure 61
Cumulative No. of Cases
Day of Outbreak
0
20
40
60
80
100
120
0
7
14
21
28
35
42
49

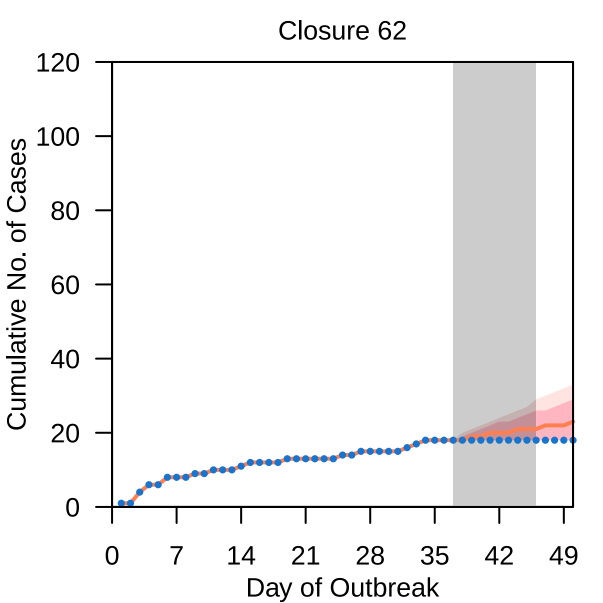

Closure 62
Cumulative No. of Cases
Day of Outbreak
0
20
40
60
80
100
120
0
7
14
21
28
35
42
49

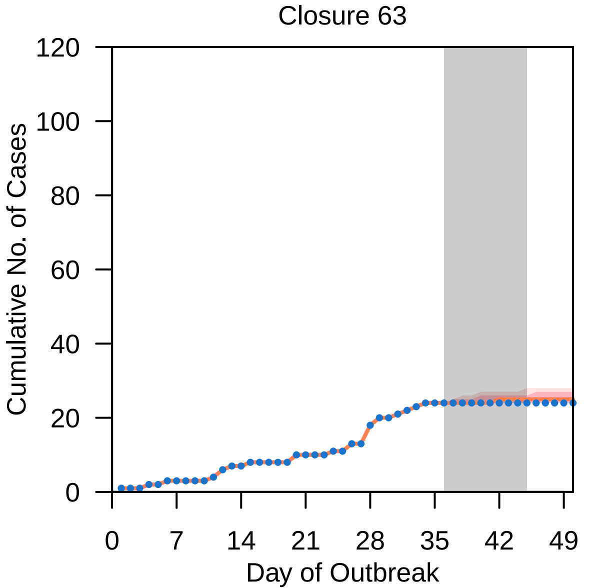

Closure 63
Cumulative No. of Cases
Day of Outbreak
0
20
40
60
80
100
120
0
7
14
21
28
35
42
49

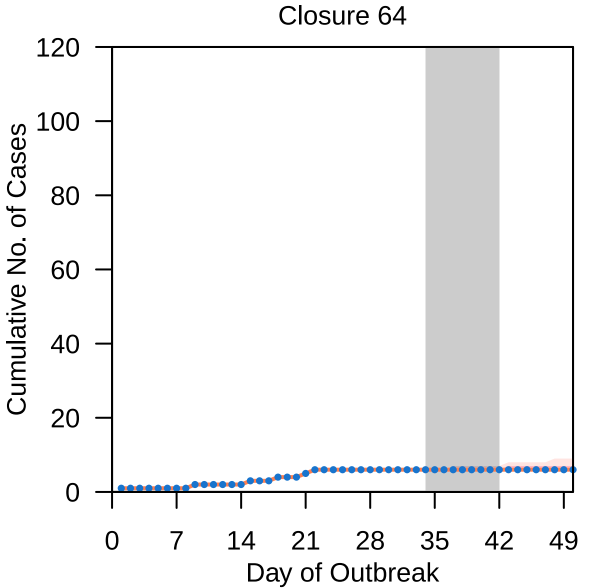

Closure 64
Cumulative No. of Cases
0
20
40
60
80
100
120
0
7
14
21
28
35
42
49
Day of Outbreak

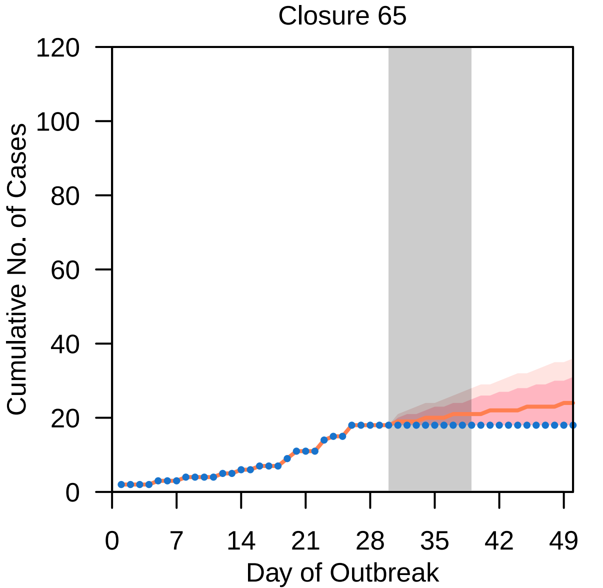

Closure 65
Cumulative No. of Cases
Day of Outbreak
0
20
40
60
80
100
120
0
7
14
21
28
35
42
49

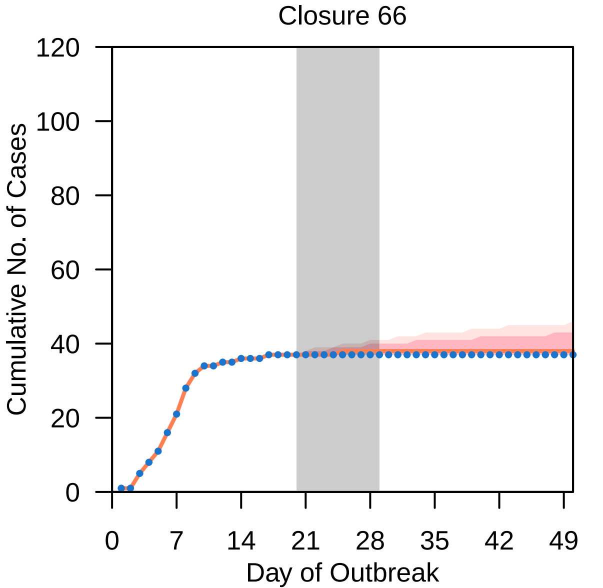

Closure 66
Cumulative No. of Cases
Day of Outbreak
0
20
40
60
80
100
120
0
7
14
21
28
35
42
49

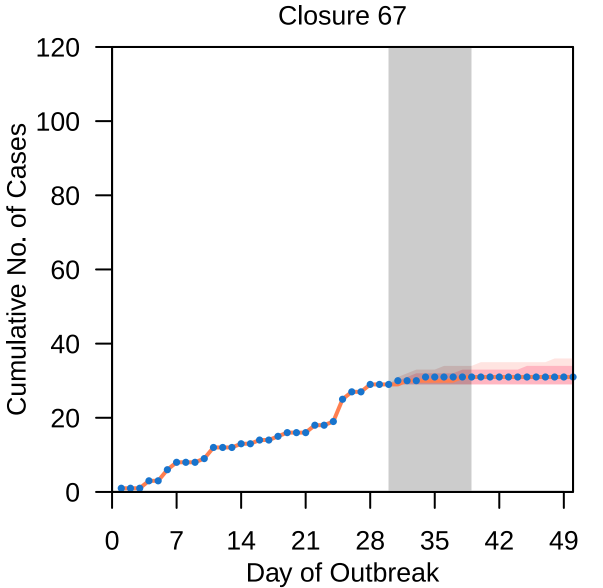
Closure 67
Cumulative No. of Cases
Day of Outbreak
0
20
40
60
80
100
120
0
7
14
21
28
35
42
49

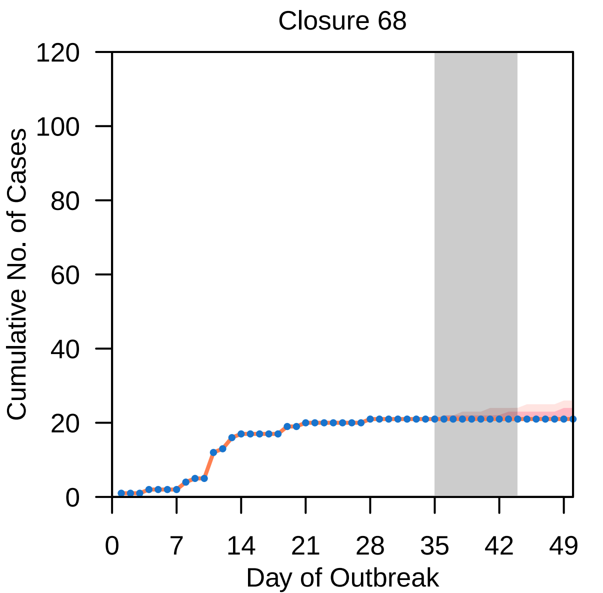

Closure 68
Cumulative No. of Cases
0
20
40
60
80
100
120
0
7
14
21
28
35
42
49
Day of Outbreak

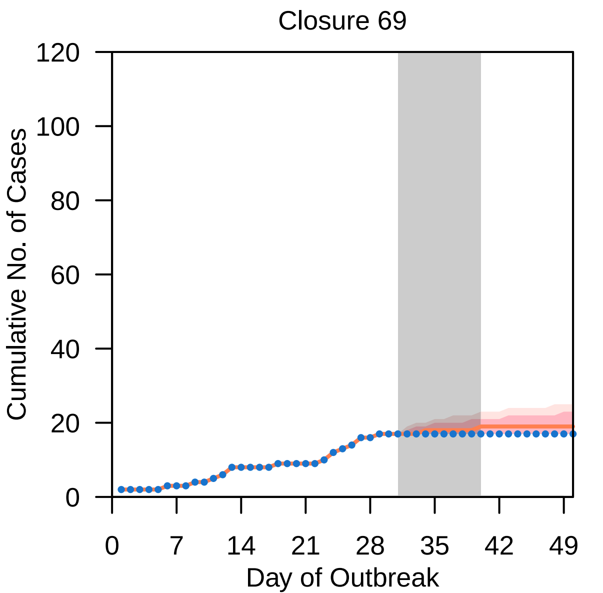

Closure 69
120
100
80
60
40
20
0
Cumulative No. of Cases
0
7
14
21
28
35
42
49
Day of Outbreak

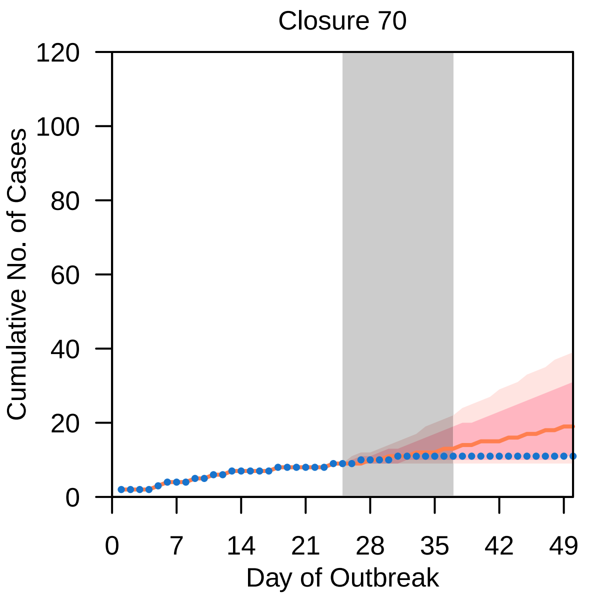
Closure 70
Cumulative No. of Cases
0
20
40
60
80
100
120
0
7
14
21
28
35
42
49
Day of Outbreak

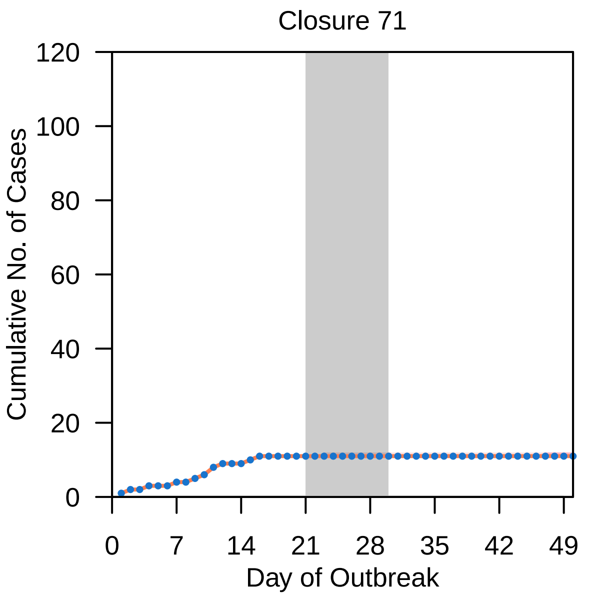
Closure 71
Cumulative No. of Cases
120
100
80
60
40
20
0
0
7
14
21
28
35
42
49
Day of Outbreak

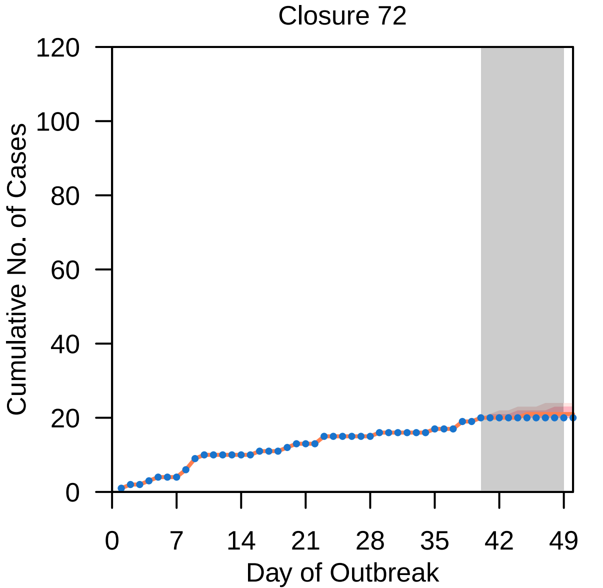

Closure 72
Cumulative No. of Cases
0
20
40
60
80
100
120
0
7
14
21
28
35
42
49
Day of Outbreak

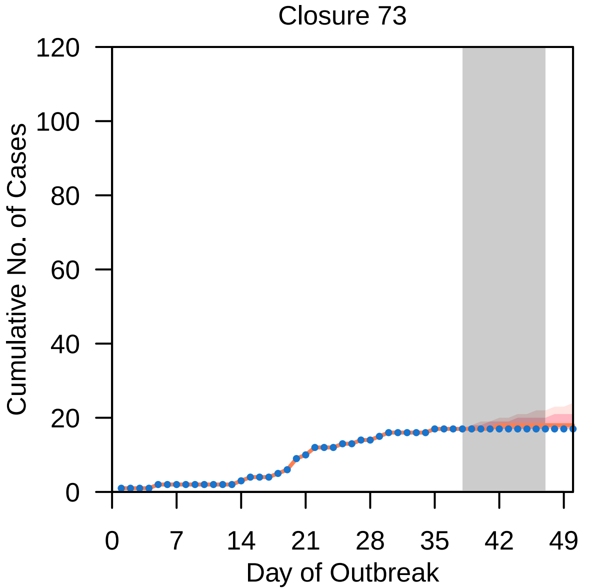
Closure 73
Cumulative No. of Cases
Day of Outbreak
0
20
40
60
80
100
120
0
7
14
21
28
35
42
49

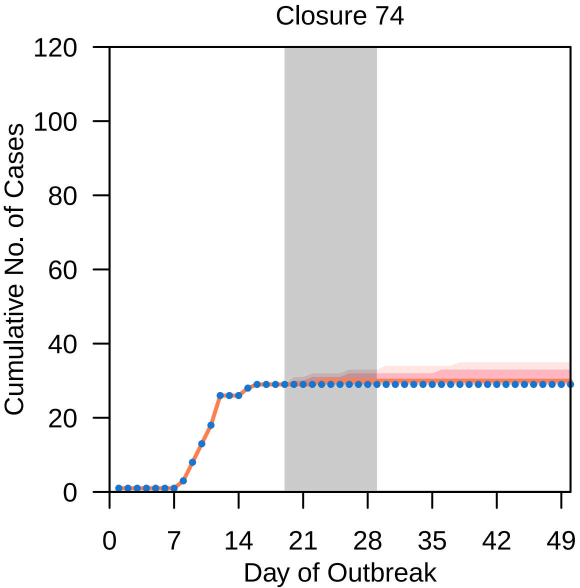
Closure 74
Cumulative No. of Cases
0
20
40
60
80
100
120
0
7
14
21
28
35
42
49
Day of Outbreak

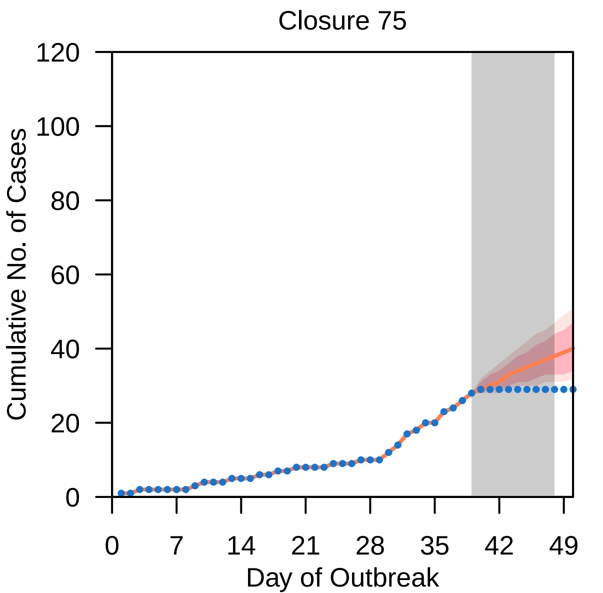

Closure 75
Cumulative No. of Cases
Day of Outbreak
0
20
40
60
80
100
120
0
7
14
21
28
35
42
49

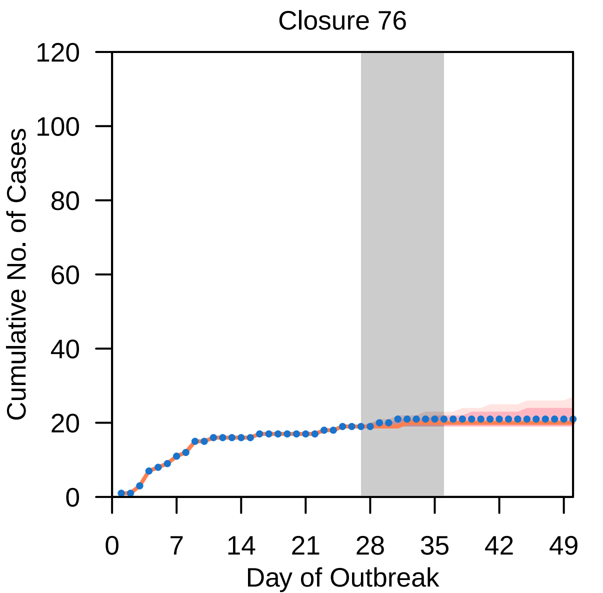
Closure 76
Cumulative No. of Cases
0
20
40
60
80
100
120
0
7
14
21
28
35
42
49
Day of Outbreak

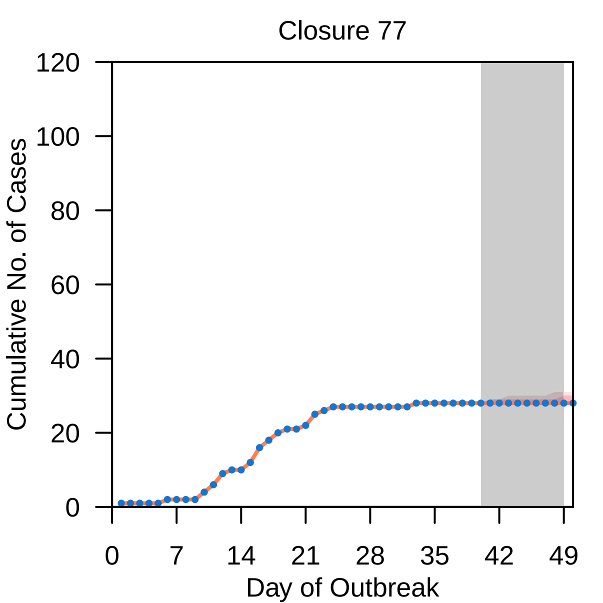

Closure 77
Cumulative No. of Cases
Day of Outbreak
0
20
40
60
80
100
120
0
7
14
21
28
35
42
49

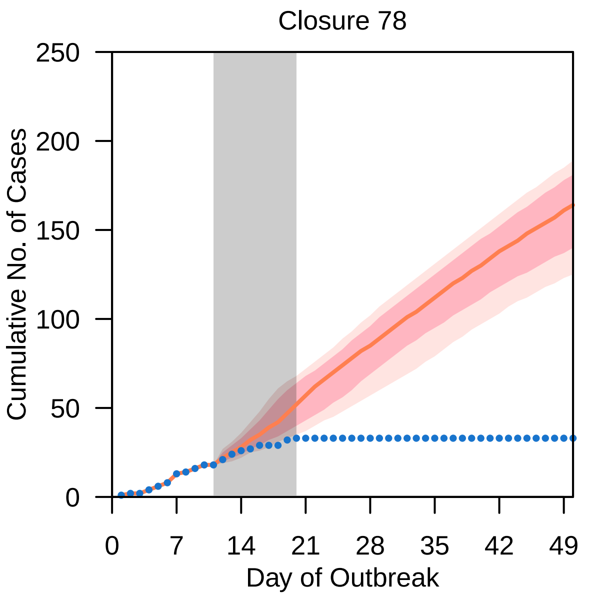

Closure 78
Cumulative No. of Cases
250
200
150
100
50
0
0
7
14
21
28
35
42
49
Day of Outbreak

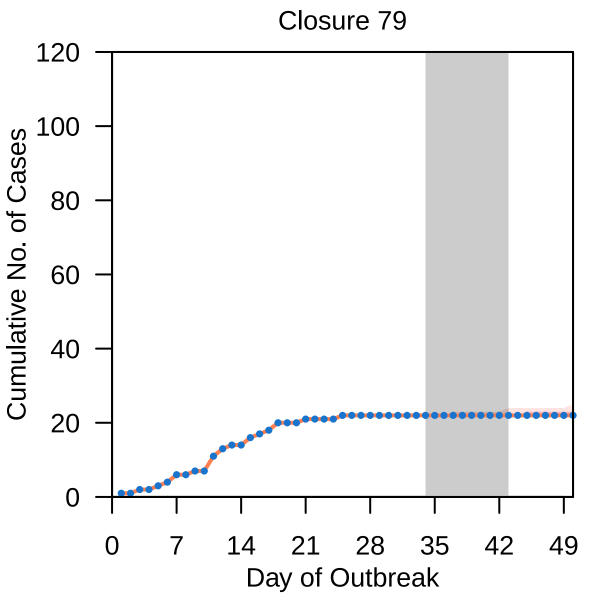

Closure 79
Cumulative No. of Cases
0
20
40
60
80
100
120
0
7
14
21
28
35
42
49
Day of Outbreak

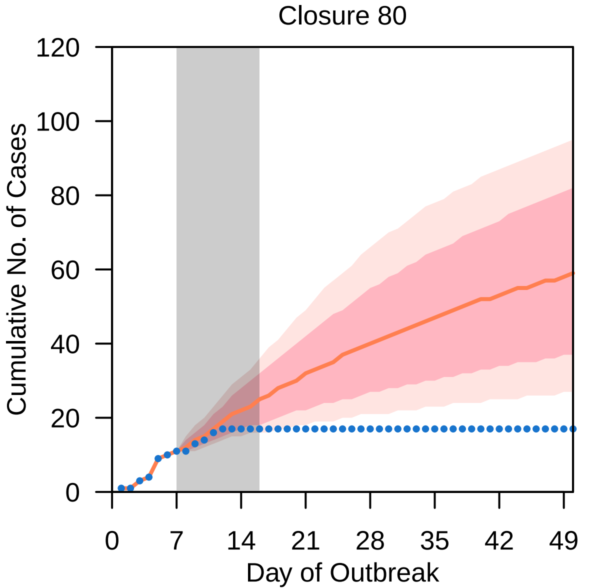

Closure 80
Cumulative No. of Cases
Day of Outbreak
0
20
40
60
80
100
120
0
7
14
21
28
35
42
49

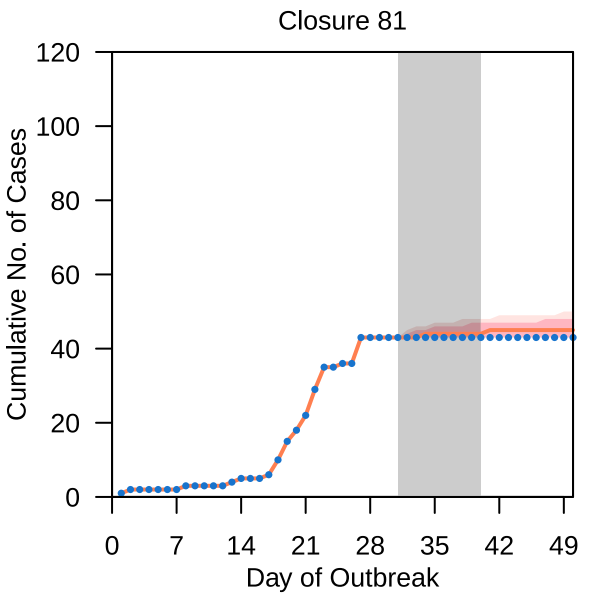

Closure 81
Cumulative No. of Cases
Day of Outbreak
0
20
40
60
80
100
120
0
7
14
21
28
35
42
49

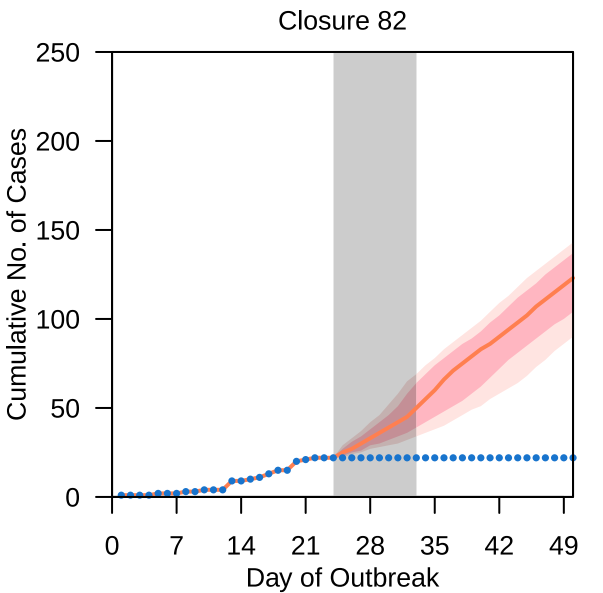

Closure 82
250
200
150
100
50
0
Cumulative No. of Cases
0
7
14
21
28
35
42
49
Day of Outbreak

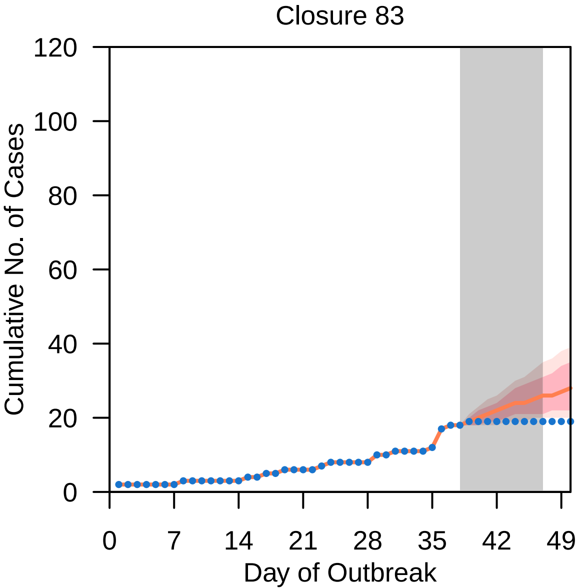

Closure 83
Cumulative No. of Cases
0
20
40
60
80
100
120
0
7
14
21
28
35
42
49
Day of Outbreak

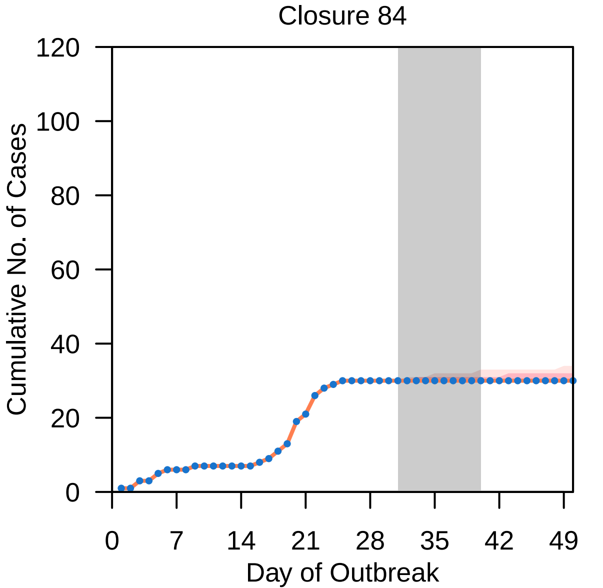

Closure 84
Cumulative No. of Cases
Day of Outbreak
0
20
40
60
80
100
120
0
7
14
21
28
35
42
49

Closure 85

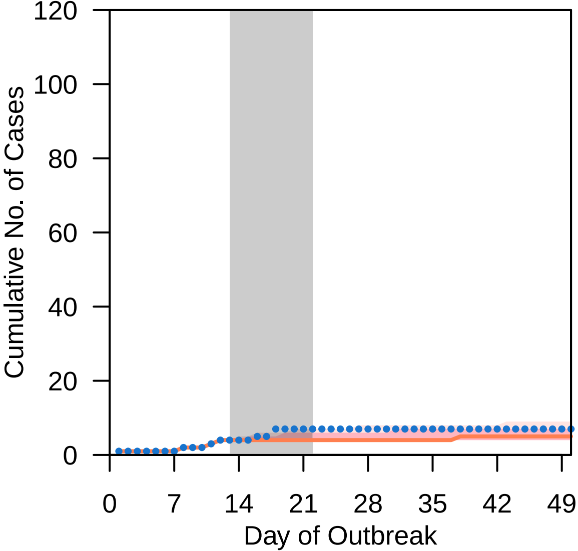
Cumulative No. of Cases
0
20
40
60
80
100
120
0
7
14
21
28
35
42
49
Day of Outbreak

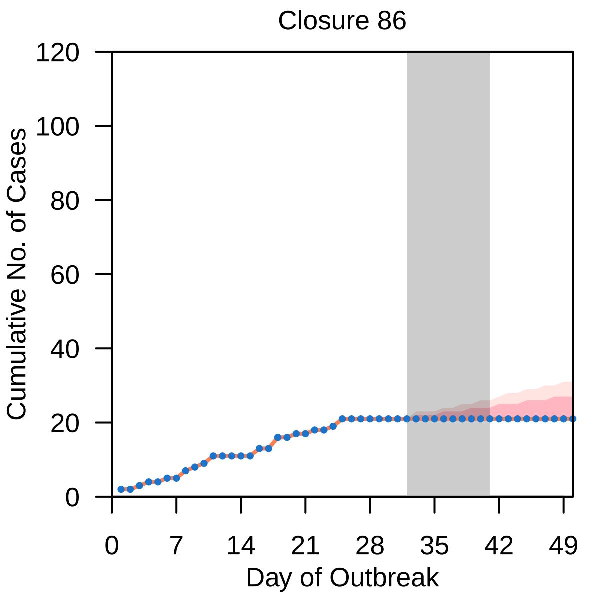

Closure 86
Cumulative No. of Cases
Day of Outbreak
0
20
40
60
80
100
120
0
7
14
21
28
35
42
49

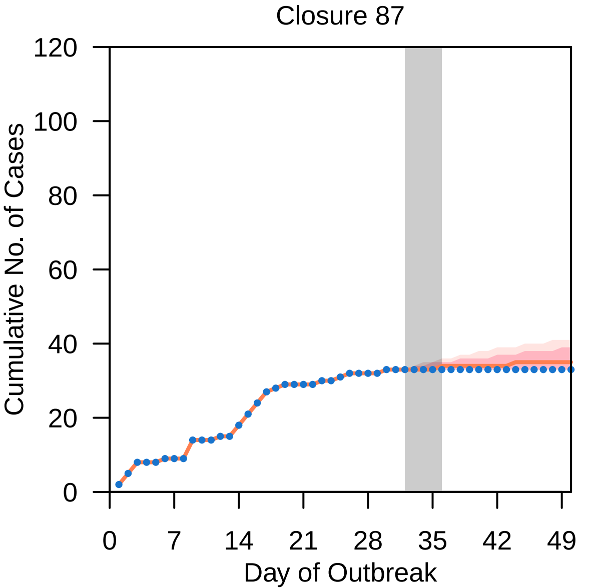
Closure 87
Cumulative No. of Cases
0
20
40
60
80
100
120
0
7
14
21
28
35
42
49
Day of Outbreak

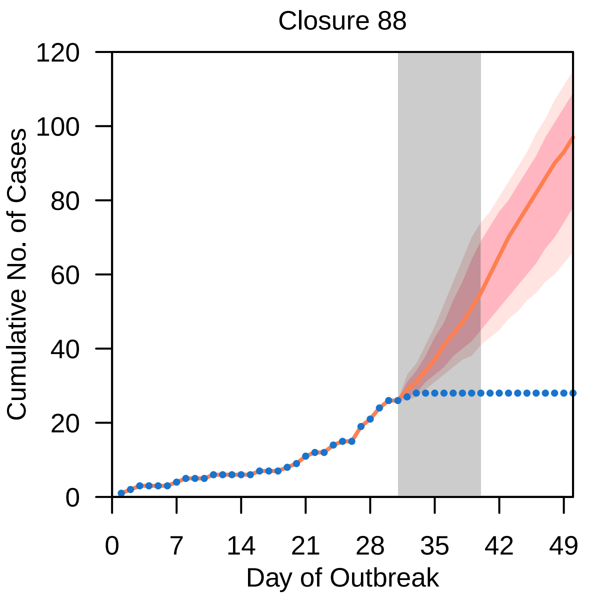

Closure 88
Cumulative No. of Cases
Day of Outbreak
0
20
40
60
80
100
120
0
7
14
21
28
35
42
49

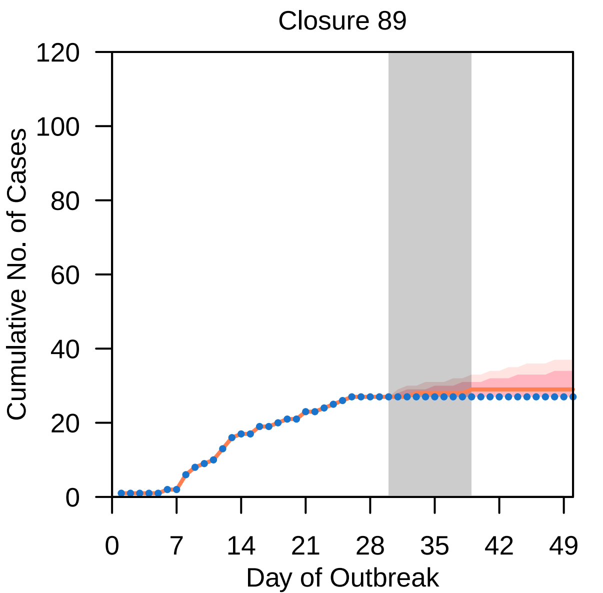

Closure 89
Cumulative No. of Cases
0
20
40
60
80
100
120
0
7
14
21
28
35
42
49
Day of Outbreak

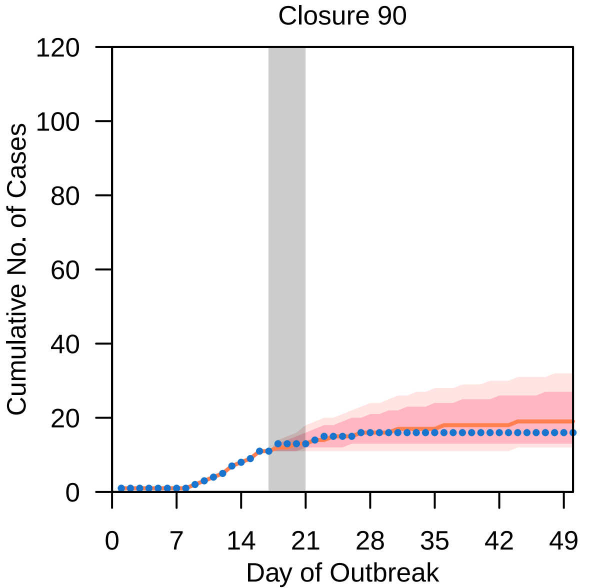
Closure 90
120
100
80
60
40
20
0
Cumulative No. of Cases
0
7
14
21
28
35
42
49
Day of Outbreak

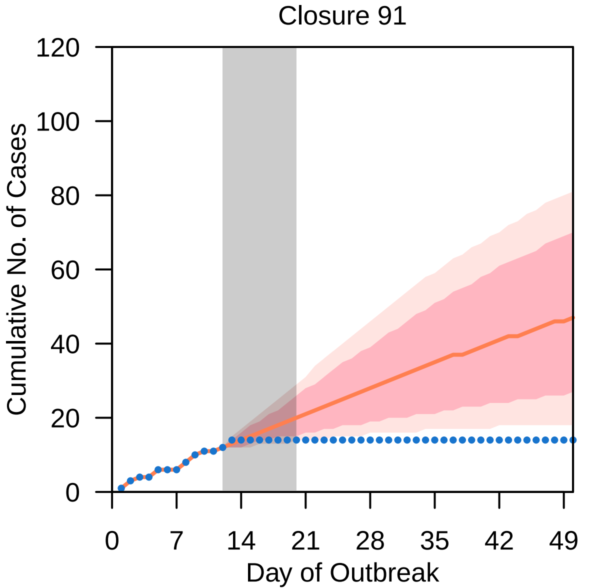

Closure 91
Cumulative No. of Cases
0
20
40
60
80
100
120
0
7
14
21
28
35
42
49
Day of Outbreak

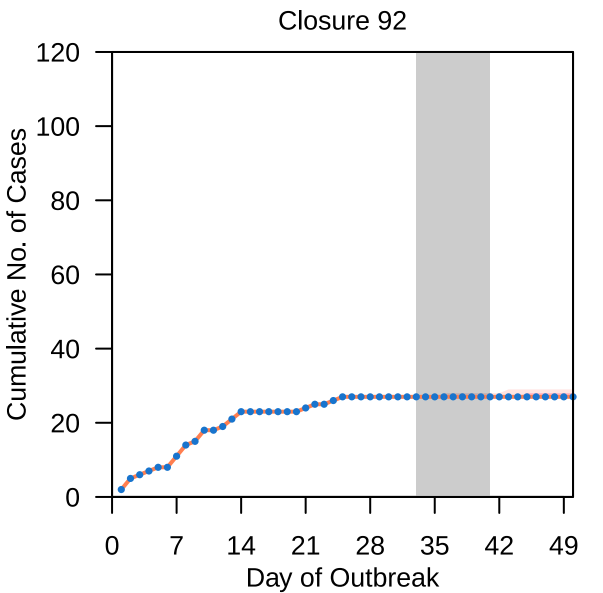
Closure 92
Cumulative No. of Cases
0
20
40
60
80
100
120
0
7
14
21
28
35
42
49
Day of Outbreak

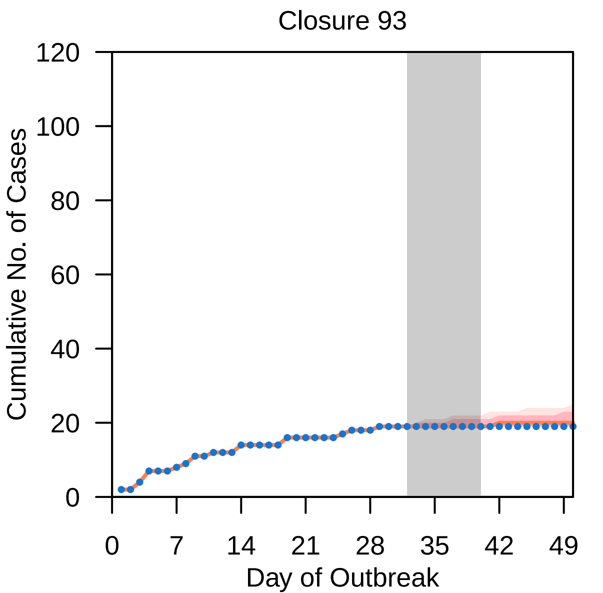

Closure 93
Cumulative No. of Cases
0
20
40
60
80
100
120
0
7
14
21
28
35
42
49
Day of Outbreak

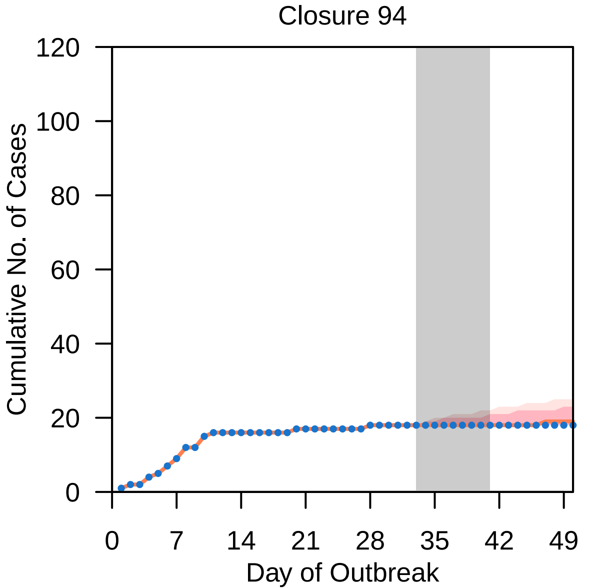

Closure 94
Cumulative No. of Cases
Day of Outbreak
0
20
40
60
80
100
120
0
7
14
21
28
35
42
49

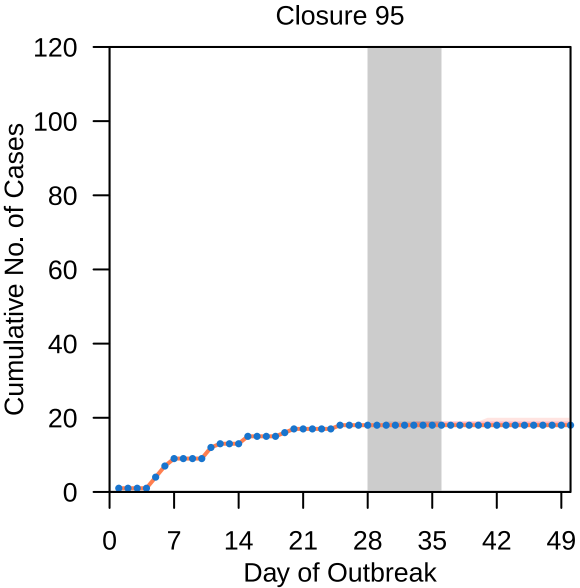

Closure 95
Cumulative No. of Cases
120
100
80
60
40
20
0
0
7
14
21
28
35
42
49
Day of Outbreak

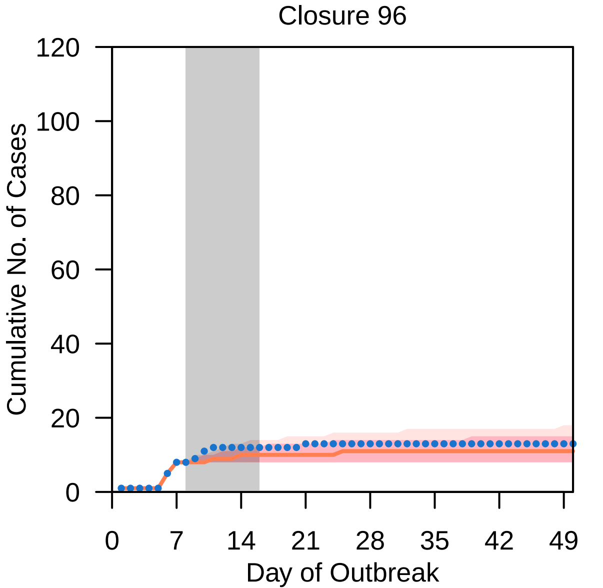

Closure 96
120
100
80
60
40
20
0
Cumulative No. of Cases
0
7
14
21
28
35
42
49
Day of Outbreak

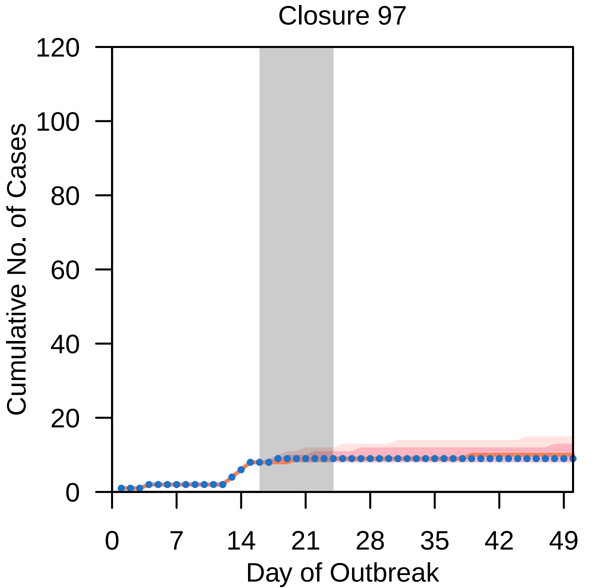
Closure 97
Cumulative No. of Cases
0
20
40
60
80
100
120
0
7
14
21
28
35
42
49
Day of Outbreak

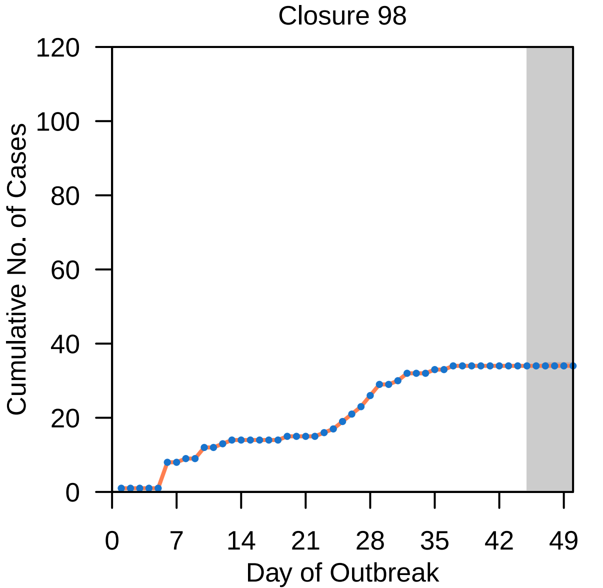
Closure 98
Cumulative No. of Cases
120
100
80
60
40
20
0
0
7
14
21
28
35
42
49
Day of Outbreak

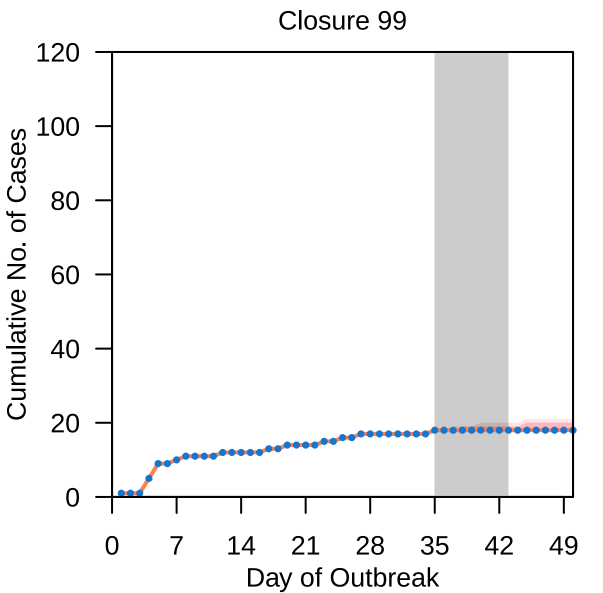
Closure 99
Cumulative No. of Cases
0
20
40
60
80
100
120
0
7
14
21
28
35
42
49
Day of Outbreak

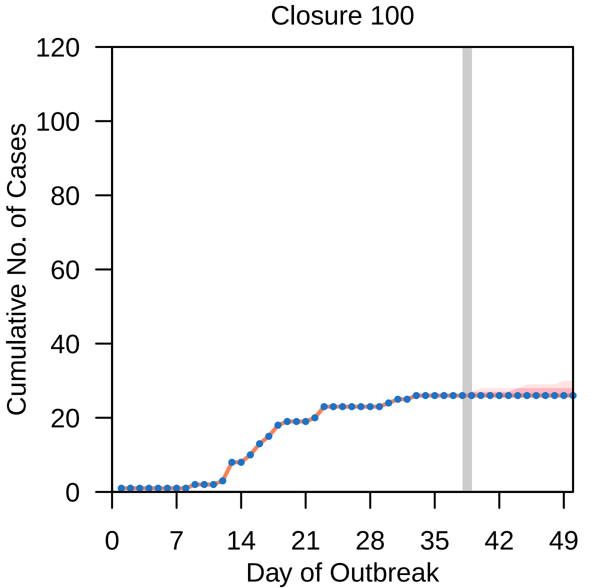

Closure 100
Cumulative No. of Cases
Day of Outbreak
0
20
40
60
80
100
120
0
7
14
21
28
35
42
49
