# Supplementary figures and images for: The Effect of School Closure on Hand, Foot, and Mouth Disease Transmission in Singapore: A Modeling Approach
Source: Am J Trop Med Hyg. 2018 Oct 22;99(6):1625–32. doi: 10.4269/ajtmh.18-0099 (PMC6283473; doi:10.4269/ajtmh.18-0099)

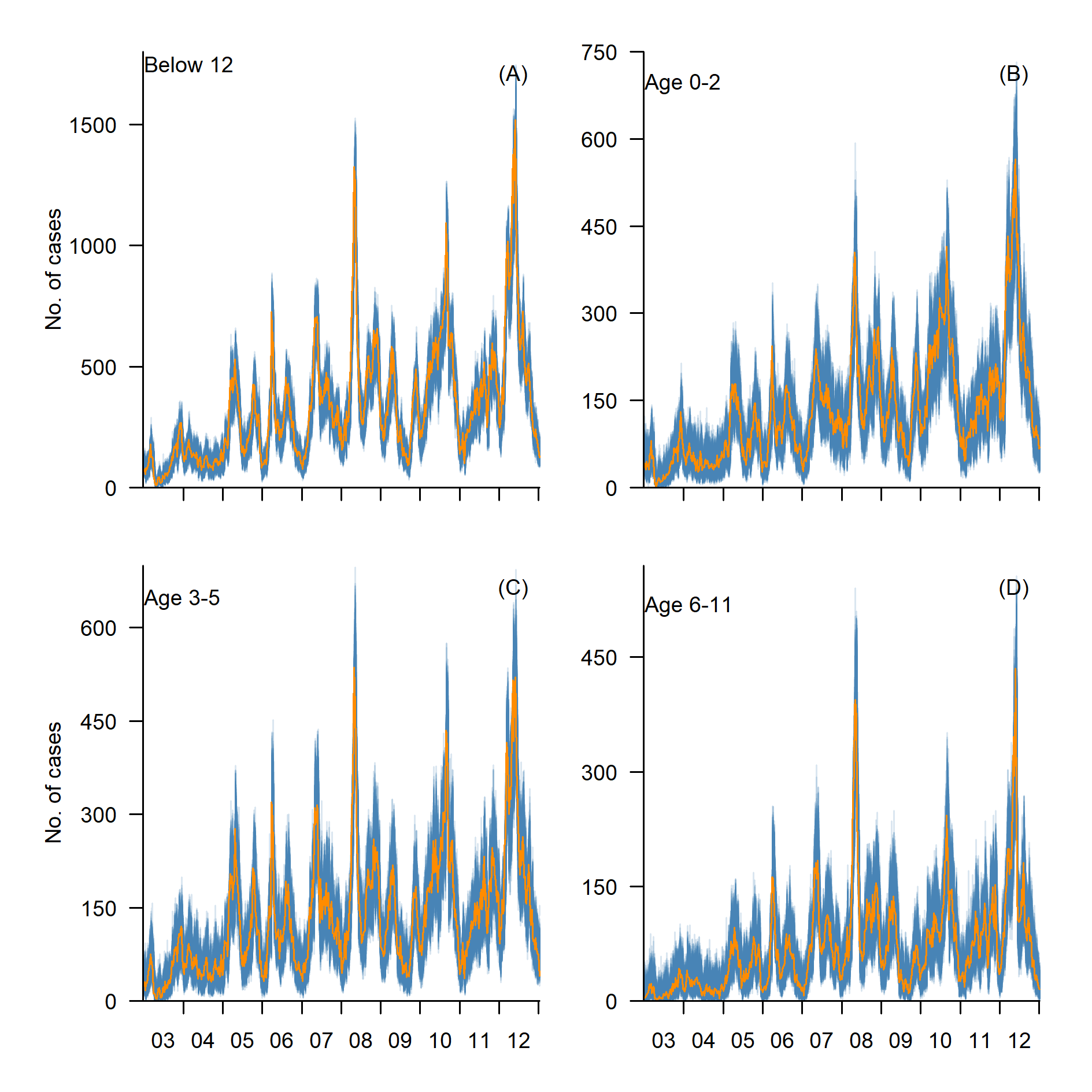

Supplement: Supplementary file 4 [file tpmd180099.SD4.png]

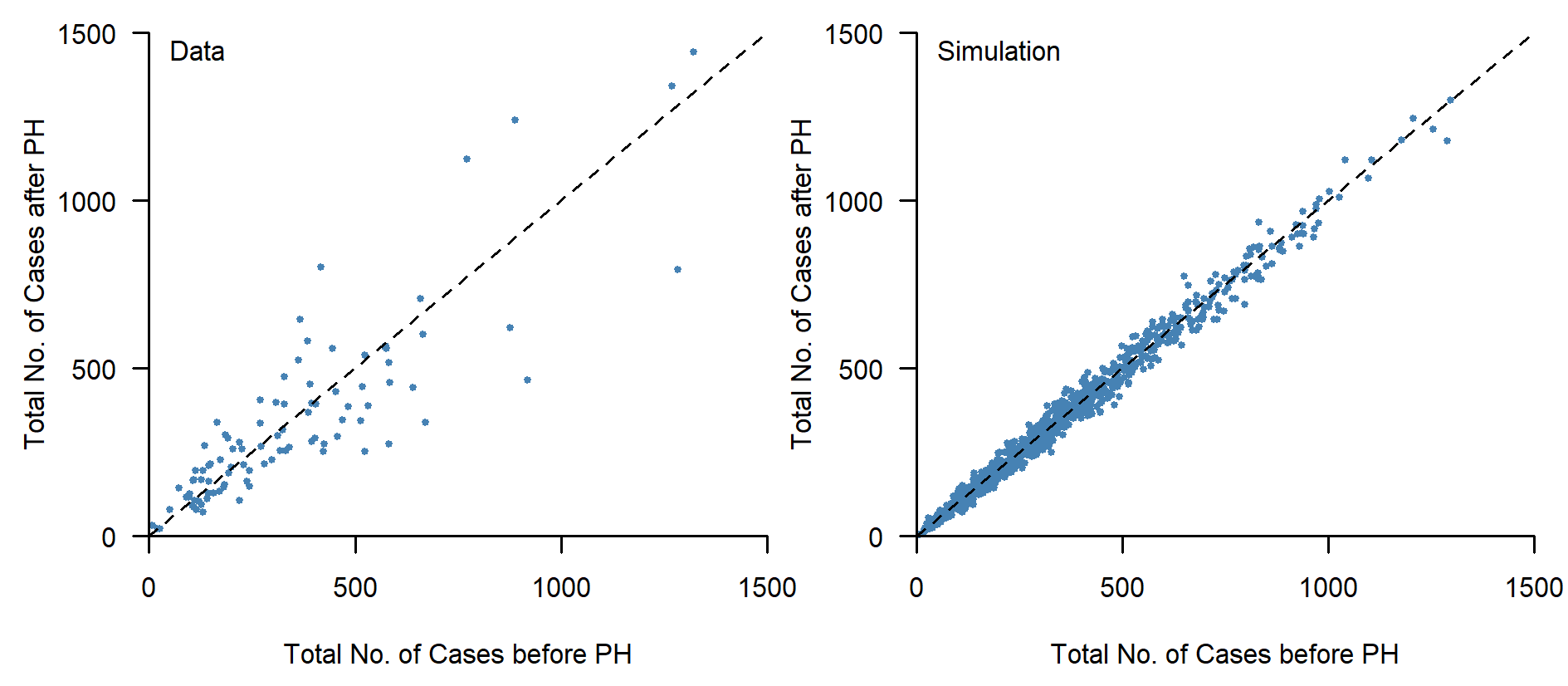

Supplement: Supplementary file 5 [file tpmd180099.SD5.png]

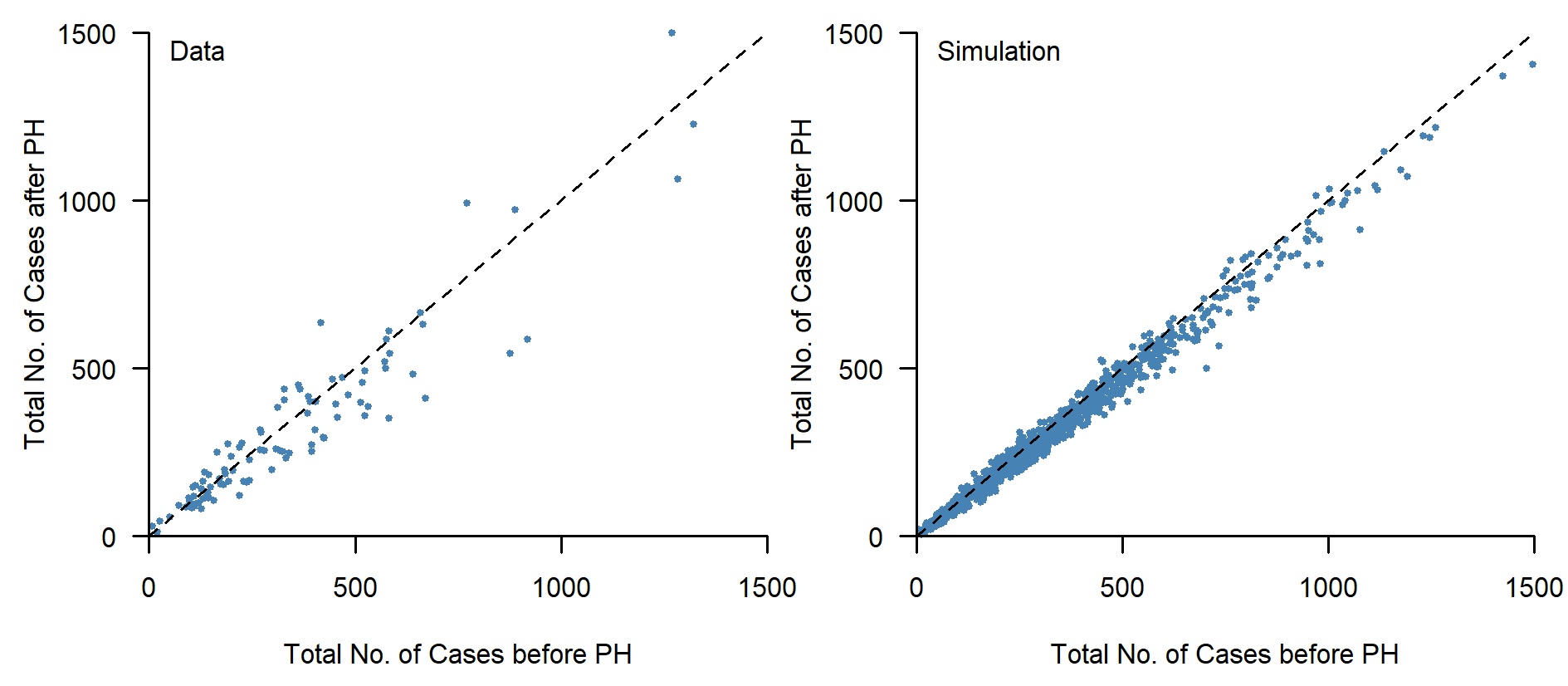

Supplement: Supplementary file 6 [file tpmd180099.SD6.png]

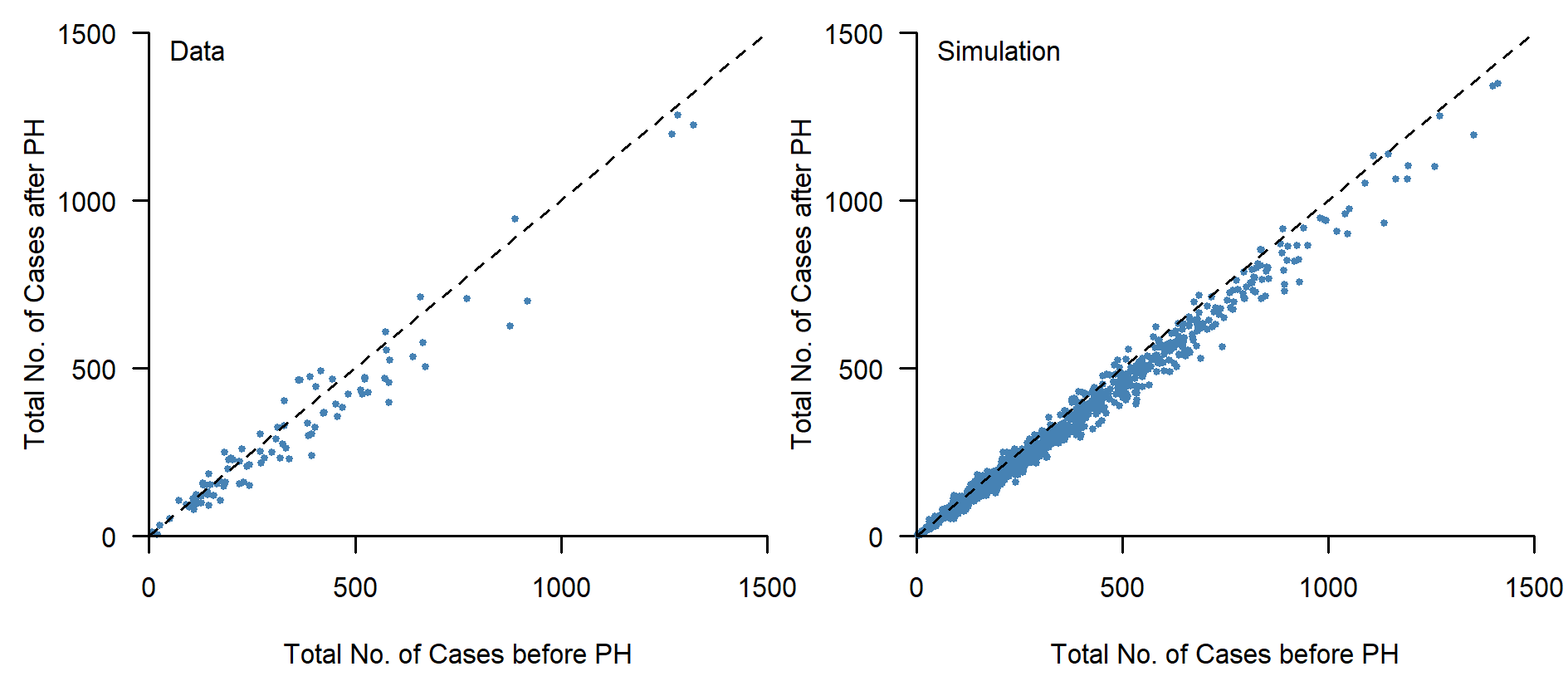

Supplement: Supplementary file 7 [file tpmd180099.SD7.png]

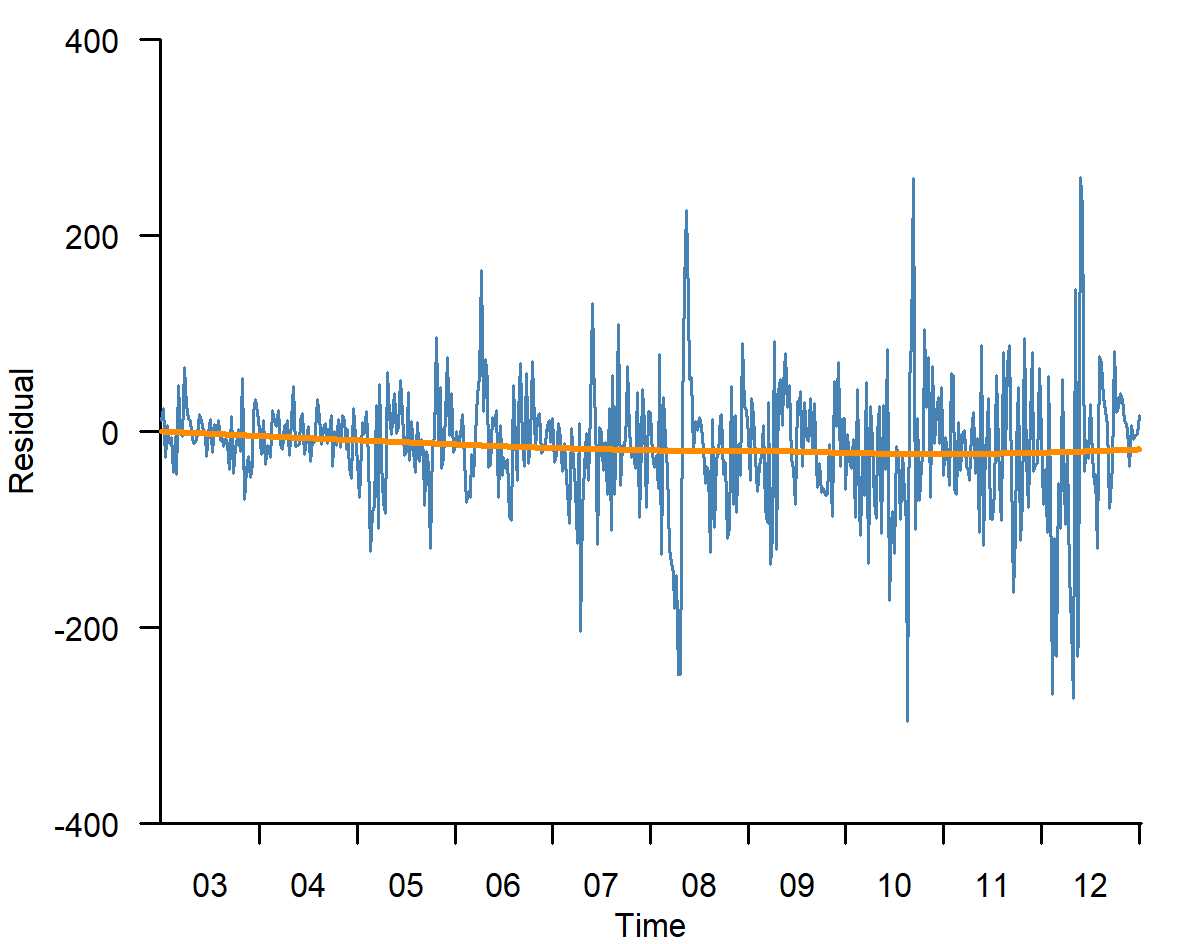

Supplement: Supplementary file 8 [file tpmd180099.SD8.png]
